# Gabapentin for Pain Management after Major Surgery: A Placebo-controlled, Double-blinded, Randomized Clinical Trial (the GAP Study)

**DOI:** 10.1097/ALN.0000000000005655

**Published:** 2025-07-15

**Authors:** Sarah Baos, Mandy Lui, Terrie Walker-Smith, Maria Pufulete, David Messenger, Reyad Abbadi, Tim Batchelor, Gianluca Casali, Mark Edwards, Nick Goddard, Mohammed Abu Hilal, Aiman Alzetani, Marius Vaida, Petr Martinovsky, Palinikumar Saravanan, Tim Cook, Rajiv Malhotra, Anna Simpson, Ross Little, Sarah Wordsworth, Elizabeth Stokes, Jingjing Jiang, Barnaby Reeves, Lucy Culliford, Laura Collett, Rachel Maishman, Nilesh Chauhan, Liz McCullagh, Holly McKeon, Samantha Abbs, Jenny Lamb, Anna Gilbert, Chloe Hughes, David Wynick, Gianni Angelini, Mike Grocott, Ben Gibbison, Chris A. Rogers

**Affiliations:** 1 Bristol Trials Centre, University of Bristol, Bristol, United Kingdom.; 2 Bristol Trials Centre, University of Bristol, Bristol, United Kingdom.; 3 Bristol Trials Centre, University of Bristol, Bristol, United Kingdom.; 4 Bristol Medical School, University of Bristol, Bristol, United Kingdom.; 5 Department of Surgery, University Hospitals Bristol and Weston NHS Foundation Trust, Bristol, United Kingdom.; 6 Department of Surgery, University Hospitals Bristol and Weston NHS Foundation Trust, Bristol, United Kingdom.; 7 Department of Surgery, University Hospitals Bristol and Weston NHS Foundation Trust, Bristol, United Kingdom; 8 Department of Surgery, University Hospitals Bristol and Weston NHS Foundation Trust, Bristol, United Kingdom.; 9 Department of Anaesthesia, University of Southampton, Southampton, United Kingdom.; 10 Department of Anaesthesia, University Hospital Southampton NHS Foundation Trust, Southampton, United Kingdom; 11 Department of Surgery, University Hospital Southampton NHS Foundation Trust, Southampton, United Kingdom; 12 Department of Surgery, University Hospital Southampton NHS Foundation Trust, Southampton, United Kingdom.; 13 Department of Anaesthesia, Somerset Hospitals Foundation NHS Trust, Taunton, United Kingdom.; 14 Department of Anaesthesia, University Hospitals Blackpool NHS Trust, Blackpool, United Kingdom.; 15 Department of Anaesthesia, University Hospitals Blackpool NHS Trust, Blackpool, United Kingdom.; 16 Department of Anaesthesia, Royal United Hospitals, Bath NHS Trust, Bath, United Kingdom.; 17 Department of Anaesthesia, University Hospitals Liverpool NHS Trust, Liverpool, United Kingdom.; 18 Department of Anaesthesia, University Hospitals Bristol and Weston NHS Foundation Trust, Bristol, United Kingdom.; 19 Department of Anaesthesia, University Hospitals Liverpool NHS Trust, Liverpool, United Kingdom; 20 Nuffield Deaprtment of Population Health, University of Oxford, Oxford, United Kingdom.; 21 Nuffield Deaprtment of Population Health, University of Oxford, Oxford, United Kingdom.; 22 Nuffield Deaprtment of Population Health, University of Oxford, Oxford, United Kingdom.; 23 Bristol Trials Centre, University of Bristol, Bristol, United Kingdom.; 24 Bristol Trials Centre, University of Bristol, Bristol, United Kingdom.; 25 Bristol Trials Centre, University of Bristol, Bristol, United Kingdom.; 26 Bristol Trials Centre, University of Bristol, Bristol, United Kingdom.; 27 Department of Anaesthesia, University Hospitals Bristol and Weston NHS Foundation Trust, Bristol, United Kingdom; 28 Department of Pharmacy, University Hospitals Bristol and Weston NHS Foundation Trust, Bristol, United Kingdom.; 29 Bristol Trials Centre, University of Bristol, Bristol, United Kingdom.; 30 Bristol Trials Centre, University of Bristol, Bristol, United Kingdom.; 31 Bristol Trials Centre, University of Bristol, Bristol, United Kingdom.; 32 Bristol Trials Centre, University of Bristol, Bristol, United Kingdom.; 33 Bristol Trials Centre, University of Bristol, Bristol, United Kingdom.; 34 University of Bristol and University Hospitals Bristol and Weston NHS Foundation Trust, Bristol, United Kingdom.; 35 Department of Cardiac Surgery, University of Bristol and University Hospitals Bristol and Weston NHS Foundation Trust, Bristol, United Kingdom.; 36 Department of Anaesthesia, University of Southampton, Upper Shirley, United Kingdom.; 37 Bristol Medical School, University of Bristol and Department of Anaesthesia, University Hospitals Bristol and Weston NHS Foundation Trust, Bristol, United Kingdom.; 38 Bristol Trials Centre, University of Bristol, Bristol, United Kingdom.

## Abstract

**Background:**

Gabapentin is an anticonvulsant medication with approval for use in neuropathic pain and epileptic disorders. It is frequently added to multimodal analgesic regimens during and after surgery to reduce opioid use while controlling pain effectively. There is little evidence to show its effectiveness in major surgery.

**Methods:**

In this multicenter, double-blinded randomized controlled trial, adults undergoing major cardiac, thoracic, or abdominal surgery were randomized to receive either gabapentin (600 mg before surgery, 300 mg twice daily for 2 days after surgery) or placebo. The primary outcome was length of hospital stay. Secondary outcomes included acute and chronic pain, total opioid use, adverse health events, and health-related quality of life. Patients were followed up daily in-hospital until discharge and then at 4 weeks and 4 months after surgery.

**Results:**

A total of 1,196 participants were randomized (500 underwent cardiac, 346 thoracic, and 350 abdominal surgery); 596 were allocated to placebo, and 600 were allocated to gabapentin. Median length of hospital stay was similar in the two groups (gabapentin, 5.94 [interquartile range (IQR), 4.08 to 8.04] days; placebo, 6.15 [IQR, 4.22 to 8.97] days; hazard ratio, 1.07; 95% CI, 0.95 to 1.20; *P* = 0.26). Overall, 384 participants experienced one or more serious adverse events (gabapentin, 189 of 596 [31.7%]; placebo, 195 of 599 [32.6%]), with some variation across surgical specialties.

**Conclusions:**

Among patients undergoing major cardiac, thoracic, and abdominal surgery, adding gabapentin to multimodal analgesic regimes did not alter the length of hospital stay or the number of serious adverse events.

## Abstract 2

In this large, multicenter, double-blinded randomized controlled trial involving adults undergoing major cardiac, thoracic, or abdominal surgery, the addition of gabapentin to multimodal analgesic regimens did not result in a clinically meaningful reduction in hospital length of stay, opioid consumption, acute pain, or quality of life. Gabapentin was associated with a higher incidence of pain at 4 months. These findings suggest that gabapentin should not be used routinely as part of the analgesic regimen in this surgical population.

## Graphical abstract

**Figure:**
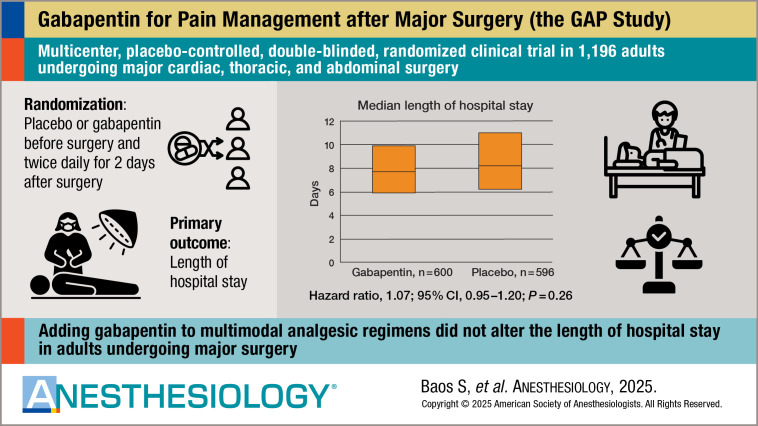

## Editor’s Perspective

**What We Already Know about This Topic**

- Gabapentin has been proposed as an important component of multimodal analgesia for managing acute postoperative pain
- Guidelines vary in their recommendations regarding the use of gabapentin in this setting

**What This Article Tells Us That Is New**

- In this large, multicenter, double-blinded randomized controlled trial involving adults undergoing major cardiac, thoracic, or abdominal surgery, the addition of gabapentin to multimodal analgesic regimens did not result in a clinically meaningful reduction in hospital length of stay, opioid consumption, acute pain, or quality of life
- Gabapentin was associated with a higher incidence of pain at 4 months
- These findings suggest that gabapentin should not be used routinely as part of the analgesic regimen in this surgical population

Gabapentin is an anticonvulsant medication with U.S. and United Kingdom regulatory approval to treat partial seizures and neuropathic pain. It reduces voltage-gated calcium channel activity in the central neurons and therefore reduces neuronal firing and neurotransmitter release.^1–3^ It is widely used “off license” in the perioperative setting as an adjunct to opioid analgesia, and its use in this setting has risen substantially in many countries.^4–6^ Opioids are the mainstay for managing moderate-to-severe pain after major surgery, but they have poor efficacy for movement-associated pain, and up to 80% of patients experience side effects including confusion, nausea, vomiting, itching, constipation, and respiratory depression.^7^ The rationale for using gabapentin is that it reduces opioid use and hence opioid-related adverse effects and promotes rapid early recovery and discharge. However, there have been concerns about the trade-off between the potential adverse effects of gabapentinoids (*e.g.*, risk of abuse and respiratory depression) and their clinical benefits.^8–13^

More than 280^14^ randomized controlled trials have compared gabapentin with placebo in different surgical populations. Most are small and highly heterogeneous, both statistically and clinically. Gabapentin can reduce opiate use by around 20% in the first 24 h after surgery.^14^ However, there is inadequate information regarding the number and impact of adverse events and quality of life, preventing policy decisions from being made.^15^ This has led to varying guidance: gabapentin is included as a “strong recommendation” as a component of multimodal analgesia for the management of postoperative pain in the United States^16^ but not in Europe.^17^ In the GAP Study, we tested the hypothesis that gabapentin reduces opioid use after surgery and speeds up recovery, therefore reducing postoperative hospital stay compared to standard multimodal analgesia (usual care).

## Materials and Methods

### Trial Design and Oversight

The GAP Study was a multicenter, parallel-group, placebo-controlled, pragmatic randomized controlled trial to compare the effectiveness, cost-effectiveness, and safety of gabapentin as an adjunct to standard multimodal analgesia. Participants, clinical care teams, and research teams were blinded to the treatment allocation. The trial protocol has been published previously^18^ and was approved by a National Health Service (NHS) Research Ethics Committee (Sheffield, United Kingdom), the United Kingdom Health Research Authority, and the Medicines and Healthcare Products Regulatory Authority. It was registered with the ISRCTN (ISRCTN63614165). All participants provided written informed consent.

### Patients

Adults aged 18 yr or older undergoing nonemergency cardiac, thoracic, or abdominal surgery were screened. Patients were expected to require a postoperative hospital stay of at least 2 days and be able to swallow during the intervention delivery period. Patients who were already taking antiepileptic medication (including gabapentinoids), who had a known allergy to gabapentin or had renal impairment (an estimated glomerular filtration rate of less than 30 ml · min^−1^ · 1.73^−1^), or who weighed less than 50 kg were excluded.^18^

### Trial Procedures

Participants were allocated in a 1:1 ratio to gabapentin or placebo using a secure internet-based randomization system. Randomization was stratified by surgical specialty and site to ensure approximately equal allocation to gabapentin and placebo in each specialty and site. Allocations were permuted blocks of varying sizes, *i.e.*, blocks of four, six, or eight.

The gabapentin group received 600 mg gabapentin preoperatively (as close to surgery as possible) and 600 mg/day (300 mg twice daily) postoperatively for 2 days, once able to swallow (*i.e.*, after extubation). The placebo group were given identical capsules at the same dosing intervals. The dose and timing of the treatment were informed by the findings and recommendations from the most recent systematic review available at the time the study was designed.^19^ Dosing windows were classified as 6 h either side of the prescribed time point. Other analgesia prescribed (*i.e.*, the standard multimodal regimen used) was at the discretion of the treating clinician.

### Outcomes

Patients were followed up daily while in the hospital and then at 4 weeks and 4 months after the surgery. The primary outcome was length of hospital stay, defined as time from end of surgery to hospital discharge. Secondary outcomes were (1) opioid consumption from surgery until hospital discharge and from discharge until 4 months, where all values are converted to intravenous (IV) morphine equivalents; (2) acute pain assessed using the numerical rating scale (NRS) completed at 1, 4, and 12 h postoperatively and then twice daily until discharge; (3) chronic pain measured at baseline, 4 weeks, and 4 months using the Brief Pain Inventory^20^; (4) adverse health events (any unfavorable or unintended health event) recorded from randomization to discharge and serious adverse events (SAEs, which resulted in death or prolonged hospitalization, were life-threatening, or resulted in persistent or significant disability/incapacity) from randomization until 4 months postoperatively; and (5) health-related quality of life measured using the five-level EQ-5D^21^ and Short Form 12 (SF-12)^22^ questionnaires completed at baseline, 4 weeks, and 4 months. Resource use data were also collected to support the cost-effectiveness analyses (reported separately).

### Statistical Analysis and Sample Size

The planned sample size was 1,500 participants (750 per group), with a minimum 376 participants per surgical specialty, which provided 90% power to detect a 12.5% difference in the proportion of participants discharged by the median specialty-specific length of hospital stay (*i.e.*, 50% in the placebo group *versus* 62.5% in the gabapentin group). The sample size was reduced to a minimum of 340 participants per surgical specialty (1,020 participants) after recruitment difficulties due to the COVID-19 pandemic. This provided 80% power to detect the target 12.5% difference, allowing for an observed noncompliance rate of 27%.

Analyses were by intention to treat. The primary outcome was compared between groups using Cox proportional hazards regression, stratified by specialty and site. In-hospital deaths were censored at the specialty-specific maximum observed time to discharge for survivors. Withdrawals before discharge were censored at withdrawal. Model assumptions were assessed graphically (see the Supplemental Digital Content for further details, https://links.lww.com/ALN/E128).

Secondary outcome models included baseline values (where measured), specialty, treatment group, and the specialty by treatment group interaction as fixed effects. Longitudinal models also included time, time by treatment group, and specialty by time by treatment group as fixed effects, with site and participant fitted as random effects. For NRS scores, the fixed effect for time was modeled using fractional polynomial functions, and time (at the participant level) was also included as a random effect. Linear mixed models were used to compare NRS and quality-of-life scores, and a two-part mixed model was used for the Brief Pain Inventory; logistic regression compares occurrence of pain and log-linear regression for the pain score, when pain was present. Opioid consumption to discharge and that from discharge to 4 months were compared between groups using log-linear and linear models, respectively. The incidence of one or more SAEs was compared using generalized linear models to obtain risk differences and risk ratios. Results for the whole study (*i.e.*, all specialties combined) are presented when a treatment group by specialty interaction was not indicated. Similarly, for longitudinal outcomes, an overall treatment difference is given if differences over time were not indicated.

Subgroup analyses of the primary outcome by sex, minimally invasive *versus* open surgery, and randomization before or after the start of COVID-19 pandemic were performed by adding subgroup and a subgroup by treatment group interaction to the model. Sensitivity analyses of the primary outcome excluded ineligible participants and participants from one site where there were concerns over data quality. The placebo is the reference group for all analyses. The results are presented as treatment effects with 95% CI without adjustment. All statistical analyses were performed using Stata software, version 17.0 (StataCorp, USA). Further analytical details are given in the Supplemental Digital Content.

## Results

### Participants

**Fig. 1.**
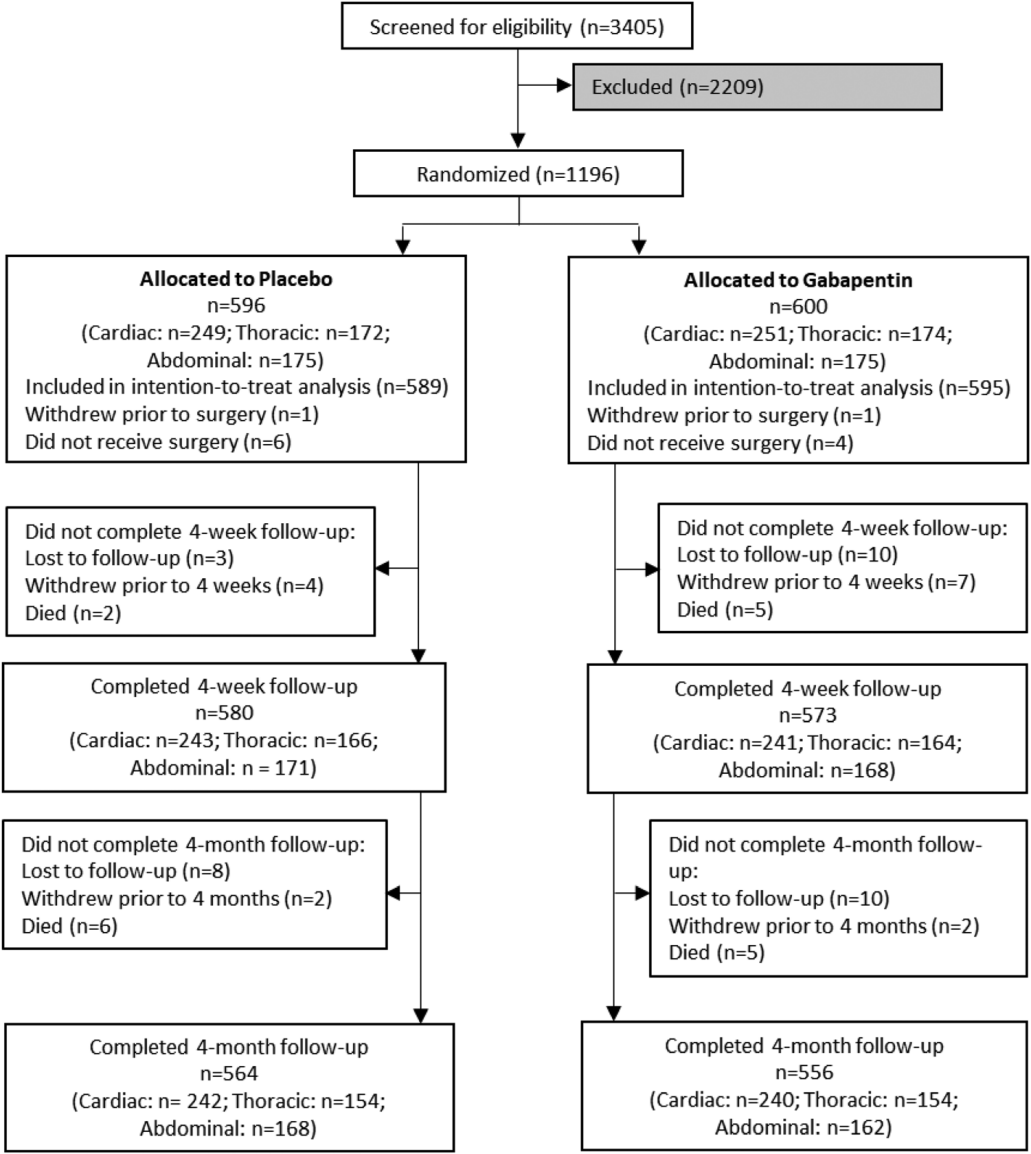
Participant flow through the trial.

Between April 2018 and May 2022, 3,405 patients were assessed for eligibility in seven United Kingdom NHS hospitals, of whom 2,209 were excluded. Reasons for exclusion can be found in the Supplemental Digital Content. Therefore, 1,196 participants were randomized into the study (596 allocated to placebo and 600 to gabapentin; fig. 1). Follow-up data at 4 weeks and 4 months were available for 1,153 of 1,196 (96.4%) and 1,120 of 1,196 (93.6%) randomized participants, respectively. Baseline characteristics were well balanced across the groups; the median age was 68 (interquartile range [IQR], 60 to 74) yr, 794 of 1,195 (66.4%) patients were male, 1,174 of 1,193 (98.4%) were of white/Caucasian ethnicity, and median body mass index was 27.3 (24.4 to 30.9) kg/m^2^ (table 1; supplemental table 1, https://links.lww.com/ALN/E128).

**Table 1.** Recipient Characteristics

Characteristic	Cardiac (n = 499)		Thoracic (n = 346)		Abdominal (n = 350)		Overall (n = 1,195)	
	Randomized to Placebo (n = 249)	Randomized to Gabapentin (n = 250)	Randomized to Placebo (n = 172)	Randomized to Gabapentin (n = 174)	Randomized to Placebo (n = 175)	Randomized to Gabapentin (n = 175)	Randomized to Placebo (n = 596)	Randomized to Gabapentin (n = 599)
Demographics								
Age, yr*	70.0 (62, 76)	69.0 (61, 74)	69.0 (60, 75)	67.0 (59, 74)	66 (57, 73)	66 (57, 72)	69 (60, 75)	68 (59, 74)
Male sex	190/249 (76.3%)	196/250 (78.4%)	95/172 (55.2%)	94/174 (54%)	103/175 (58.9%)	116/175 (66.3%)	388/596 (65.1%)	406/599 (67.8%)
White/Caucasian	242/248 (97.6%)	242/249 (97.2%)	171/172 (99.4%)	173/174 (99.4%)	172/175 (98.3%)	174/175 (99.4%)	585/595 (98.3%)	589/598 (98.5%)
Asian/Asian British	3/248 (1.2%)	2/249 (0.8%)	1/172 (0.6%)	0/174 (0.0%)	0/175 (0.0%)	0/175 (0.0%)	4/595 (0.7%)	2/598 (0.3%)
Black/Black British	1/248 (0.4%)	0/249 (0.0%)	0/172 (0.0%)	0/174 (0.0%)	2/175 (1.1%)	1/175 (0.6%)	3/595 (0.5%)	1/598 (0.2%)
Mixed/multiple/other ethnic group	2/248 (0.8%)	5/249 (2.0%)	0/172 (0.0%)	1/174 (0.6%)	1/175 (0.6%)	0/175 (0.0%)	3/595 (0.5%)	6/598 (1.0%)
Body mass index†	27.4 (24.3,31.3)	27.8 (25.3,30.8)	26.8 (24.1,30.8)	26.2 (23.1,29.7)	27.8 (24.9,31.5)	27.3 (24.6,30.7)	27.4 (24.4, 31.2)	27.1 (24.5, 30.5)
ASA status								
I	5/245 (2.04%)	1/249 (0.4%)	5/171 (2.9%)	7/174 (4.0%)	8/174 (4.6%)	5/175 (2.9%)	18/590 (3.1%)	13/598 (2.2%)
II	22/245 (9.00%)	26/249 (10.4%)	106/171 (62.0%)	104/174 (59.8%)	125/174 (71.8%)	125/175 (71.4%)	253/590 (42.9%)	255/598 (42.6%)
III	203/245 (82.9%)	210/249 (84.3%)	60/171 (35.1%)	63/174 (36.2%)	40/174 (23.0%)	45/175 (25.7%)	303/590 (51.4%)	318/598 (53.2%)
IV	15/245 (6.1%)	12/249 (4.8%)	0/171	0/174	1/174 (0.6%)	0/175	16/590 (2.7%)	12/598 (2.0%)
Medical history								
Nondiabetic	203/248 (81.9%)	204/249 (81.9%)	147/172 (85.5%)	154/173 (89%)	151/175 (86.3%)	151/175 (86.3%)	501/595 (84.2%)	509/597 (85.3%)
DM with oral medication	26/248 (10.5%)	28/249 (11.2%)	15/172 (8.7%)	10/173 (5.8%)	9/175 (5.1%)	12/175 (6.9%)	50/595 (8.4%)	50/597 (8.4%)
DM with injected medication	9/248 (3.6%)	8/249 (3.2%)	6/172 (3.5%)	6/173 (3.5%)	5/175 (2.9%)	9/175 (5.1%)	20/595 (3.4%)	23/597 (3.9%)
Diet-controlled DM	10/248 (4.0%)	9/249 (3.6%)	4/172 (2.3%)	3/173 (1.7%)	10/175 (5.7%)	3/175 (1.7%)	24/595 (4.0%)	15/597 (2.5%)
Nonsmoker	124/248 (50%)	124/249 (49.8%)	54/172 (31.4%)	52/174 (29.9%)	98/175 (56%)	92/175 (52.6%)	276/595 (46.4%)	268/598 (44.8%)
Ex smoker for >1 month	105/248 (42.3%)	97/249 (39%)	91/172 (52.9%)	83/174 (47.7%)	62/175 (35.4%)	69/175 (39.4%)	258/595 (43.4%)	249/598 (41.6%)
Current smoker	19/248 (7.7%)	28/249 (11.2%)	27/172 (15.7%)	39/174 (22.4%)	15/175 (8.6%)	14/175 (8%)	61/595 (10.3%)	81/598 (13.5%)
Medication at baseline								
Any analgesia	120/248 (48.4%)	132/249 (53.0%)	65/172 (37.8%)	70/174 (40.2%)	65/175 (37.1%)	45/175 (25.7%)	250/595 (42.0%)	247/598 (41.3%)
Opioids‡	12/120 (10.0%)	20/132 (15.2%)	34/65 (52.3%)	25/70 (35.7%)	21/65 (32.3%)	16/45 (35.6%)	67/250 (26.8%)	61/247 (24.7%)
Antidepressants	29/248 (11.7%)	26/249 (10.4%)	20/172 (11.6%)	34/174 (19.5%)	19/175 (10.9%)	19/175 (10.9%)	68/595 (11.4%)	79/598 (13.2%)
Surgery received								
Lower GI surgery					137/175 (78.3%)	132/175 (75.4%)	137/596 (11.5%)	132/599 (11.0%)
Upper GI surgery					38/175 (21.7%)	43/175 (24.6%)	38/596 (3.2%)	43/599 (3.6%)
Open surgery	247/247 (100%)	248/248 (100%)	64/169 (37.9%)	60/173 (34.7%)	93/173 (53.8%)	101/174 (58.0%)	404/589 (68.6%)	409/595 (68.7%)
Minimal access surgery			105/169 (62.1%)	113/173 (65.3%)	80/173 (46.2%)	73/174 (42.0%)	185/589 (31.4%)	186/595 (31.3%)

The data are n/N (%) or median and interquartile range.

* Missing (placebo, gabapentin): cardiac (1, 0).

† Missing (placebo, gabapentin): cardiac (1, 1).

‡ Codeine, tramadol, fentanyl, morphine (short acting or prolonged release), oxycodone (short acting or prolonged release), dihydrocodeine, or buprenorphine.

ASA, American Society of Anesthesiologists; DM, diabetes mellitus; GI, gastrointestinal.

One participant withdrew consent for their data to be used. The analysis population was therefore 1,195 participants. In total, 957 of 1,195 (80.1%) participants received all trial medication per protocol (487 of 596 [81.7%] in the placebo group and 470 of 599 [78.5%] in the gabapentin group). The most common protocol deviation was participants receiving fewer than the prescribed six capsules of trial medication or receiving medication outside of the dosing window (99 of 596 [16.6%] placebo and 124 of 599 [20.7%] gabapentin). In total, 27 participants withdrew after randomization: 13 participant decisions after surgery (1 participant moved to a nonparticipating institution, and 12 withdrew from follow-up), 2 due to clinicians deeming the participant no longer eligible, and 12 did not undergo surgery in the trial.

### Length of Hospital Stay

**Fig. 2.**
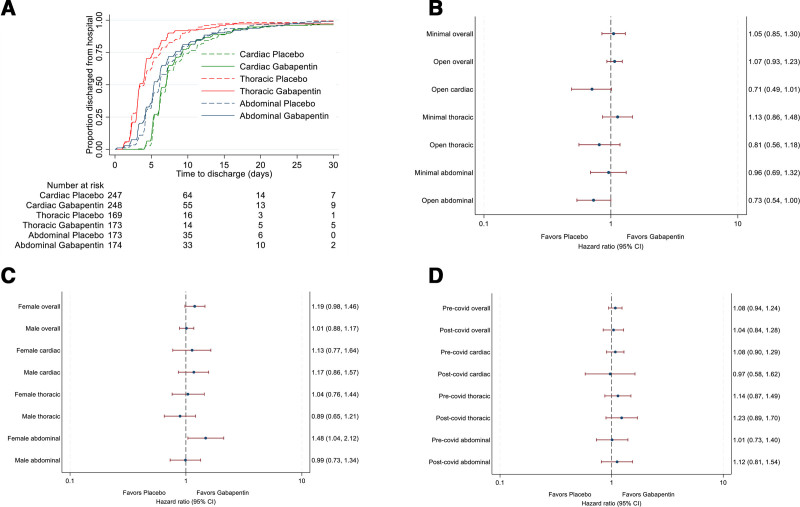
Primary outcome was the time from surgery to discharge from hospital. (*A*) Time to discharge by treatment group and surgical specialty. (*B* to *D*) show hazard ratios with 95% CI for time to discharge for the gabapentin group *versus* the placebo group by subgroup. (*B*) Open and minimally invasive surgery. (*C*) Male and female recipients. (*D*) Surgery before and after the COVID-19 pandemic.

Six participants died before discharge, four in the cardiac specialty (one placebo and three gabapentin) and two in the thoracic specialty (both gabapentin). Those in the placebo group stayed a median 6.15 (IQR, 4.22 to 8.97) days, and those in the gabapentin group stayed a median 5.94 (IQR, 4.08 to 8.04) days postoperatively (hazard ratio, 1.07; 95% CI, 0.95 to 1.20; *P* = 0.26; fig. 2A). The hazard ratio for hospital discharge was similar across the three surgical specialties (*P* = 0.94). The target of a 12.5% difference in the proportion discharged within 5 days (cardiac and abdominal specialties) or 3 days (thoracic specialty) between the groups was not met in any specialty (table 2). The sensitivity analyses did not affect the conclusions (supplemental table 2, https://links.lww.com/ALN/E128), and no subgroup differences were identified (fig. 2, B to D; supplemental table 3, https://links.lww.com/ALN/E128).

**Table 2.** Time to Hospital Discharge after Surgery

Time from Surgery to Hospital Discharge, days	Randomized to Placebo	Randomized to Gabapentin	Hazard Ratio (95% CI)	*P* Value
All participants, No.	589	593		
Median (IQR)	6.15 (4.22–8.97)	5.94 (4.08–8.04)	1.07 (0.95–1.20)	0.26
Cardiac, No.	247	248		
Median (IQR)	7.04 (5.38–10.24)	6.97 (5.27–9.20)	1.07 (0.89–1.28)	
Discharged within 5 days^*^	26.3%	27.0%		
Thoracic, No.	169	171		
Median (IQR)	3.99 (2.31–6.21)	3.41 (2.93–5.25)	1.09 (0.88–1.36)	
Discharged within 3 days^*^	48.5%	50.9%		
Abdominal, No.	173	174		
Median (IQR)	6.15 (4.21–9.17)	5.35 (4.06–8.29)	1.03 (0.83–1.29)	
Discharged within 5 days^*^	44.2%	52.3%		
Treatment by specialty interaction				0.94

Hazard ratio for time to discharge from hospital after surgery.

* Median length of stay assumed when the study was designed.

IQR, interquartile range.

### Opioid Consumption

**Fig. 3.**
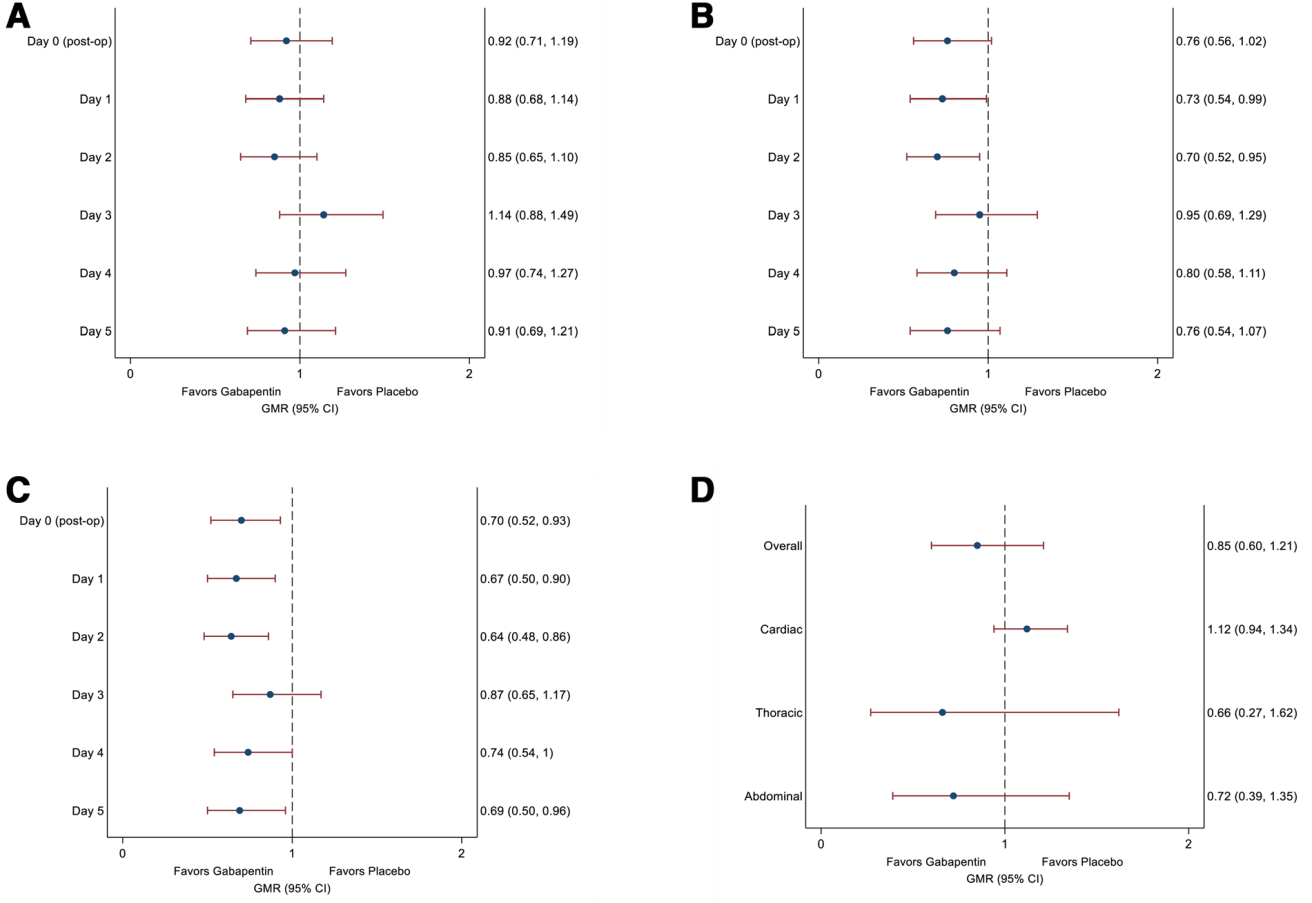
Opioid consumption after surgery to discharge and during follow-up. (*A* to *C*) Geometric mean ratios with 95% CI for opioid consumption in the first 5 days after surgery for the gabapentin group *versus* the placebo group by surgical specialty. (*A*) Cardiac. (*B*) Thoracic. (*C*) Abdominal. (*D*) Opioid consumption during follow-up in the different specialties. GMR, geometric mean ratio.

In participants undergoing cardiac surgery, there was no difference in the use of opioids, either immediately postoperatively or during follow-up. In patients undergoing thoracic surgery, participants in the gabapentin group used less opioid medication than those in the placebo group on the day of surgery and for the first 2 postoperative days (day 1: geometric mean 9.4 mg *vs.* 13.4 mg IV morphine equivalents; ratio, 0.73; 95% CI, 0.54 to 0.99) but not thereafter. Except for day 3, participants undergoing abdominal surgery used less opioid medication postoperatively (day 1: 8.5 mg *vs.* 13.8 mg IV morphine equivalents; ratio, 0.67; 95% CI, 0.50 to 0.90) but not after hospital discharge (fig. 3; supplemental table 4, https://links.lww.com/ALN/E128). A summary of all analgesics and adjuvants used by study participants is contained in supplemental table 6 (https://links.lww.com/ALN/E128).

### Pain

The maximum differences in pain measured using the NRS were within the first 24 h after surgery. The gabapentin group had pain scores of −0.81 (95% CI, −1.12 to −0.51) points lower at rest and –0.82 (95% CI, −1.20 to −0.44) points lower on movement at 1 h after the surgery. This difference reduced toward zero thereafter. At 120 h after the surgery, mean differences were −0.040 (95% CI, −0.19 to 0.11) points at rest and 0.032 (95% CI, −0.15 to 0.22) points on movement. The pattern was the same across the three surgical specialties (supplemental table 5, https://links.lww.com/ALN/E128).

The number of participants reporting pain after hospital discharge was higher in the gabapentin group compared to the placebo group at both 4 weeks (63.4% *vs.* 53.3%) and 4 months (40.6% *vs.* 33.2%) after the surgery. However, where pain was reported, the severity of the pain was similar in the two groups (geometric mean ratio, 0.99. 95% CI. 0.90 to 1.08; supplemental table 6, https://links.lww.com/ALN/E128).

### Quality of Life

The gabapentin group had similar five-level EQ-5D utility scores to the placebo group at 4 weeks and 4 months (mean difference, −0.014; 95% CI, −0.033 to 0.005) and a −0.87 (95% CI, −1.71 to −0.04) point lower SF-12 physical component score. For the SF-12 mental, the component the mean difference was 0.74 (95% CI, −0.39 to 1.87) points at 4 weeks and −0.55 (95% CI, −1.61 to 0.51) at 4 months (supplemental table 7, https://links.lww.com/ALN/E128).

### Safety

Overall, 1,453 adverse events were reported in 433 participants in the placebo group compared to 1,488 adverse events in 420 participants in the gabapentin group. In addition to these adverse events, 414 SAEs in 189 (31.7%) participants were reported in the placebo group, and 505 SAEs in 195 (32.5%) participants were reported in the gabapentin group. Three SAEs (loss of consciousness, respiratory depression, and vomiting) in the gabapentin group were classified as possible serious adverse reactions to the intervention. All resolved without sequelae. The remaining SAEs were classified as “not related” (565, 61.5%) or “unlikely to be related” (350, 38.1%). There were 18 deaths: 8 in the placebo group and 10 in the gabapentin group. Details of all adverse events are available in supplemental tables 8 and 9 (https://links.lww.com/ALN/E128).

## Discussion

### Statement of Principal Findings

The GAP Study has shown that among patients undergoing major surgery, the addition of gabapentin (600 mg preoperatively and 300 mg twice a day postoperatively for 2 days) to multimodal analgesic regimes did not reduce hospital length of stay or improve quality of life after surgery. Participants in the gabapentin group used one quarter to one third less opioid medication in-hospital and reported slightly less pain in the first 24 h after surgery, although the reduction in pain was well below the minimal clinically important difference. The small reductions in opioid use did not translate into fewer adverse events. There was no difference in opioid consumption after discharge. Participants who took gabapentin had a higher incidence of pain at 4 months, but where pain was reported, at a similar severity to the placebo group.

### Interpretation in the Context of Existing/Other Evidence

The most recent comprehensive meta-analysis,^14^ including 281 trials (24,682 participants), showed no clinically meaningful benefit of gabapentinoids on acute or chronic pain after surgery. Only 17 of 281 (2,463 participants) trials in this meta-analysis examined length of hospital stay. Length of stay is important, as it is reflective of all harms and benefits in the peri- and postoperative period, which are important to both patients and healthcare providers.^23^ While many studies of gabapentin in the perioperative period (including the GAP Study) report statistically significant differences in pain scores or opioid use, very few show clinically important differences in pain (10-mm difference on a 100-mm visual analog score^24^) or time to cessation of pain.^25^ The mean NRS scores in the GAP Study were lower in the gabapentin group at all in-hospital time points. However, at no time point and in no specialty, at rest or on movement, was the mean difference in NRS score more than 1 point on a 10-point scale. Gabapentin also did not improve the incidence of or the experience of longer-term pain.

The GAP Study showed reductions of around one quarter to one third in the use of opioids for those undergoing thoracic and abdominal surgery in-hospital. These reductions were most marked during the period of intervention (*i.e.*, for the first 2 days after surgery). However, these reductions were modest when viewed as absolute reductions in opioid use (maximum observed difference: abdominal surgery, day 2 postoperatively: placebo median 21.8 [IQR, 9.9 to 40.3] mg IV morphine equivalents *versus* gabapentin 14.5 (IQR, 4.4 to 32.1) mg IV morphine equivalents). The most cited risks of gabapentin in the postoperative period are somnolence and respiratory depression, particularly when combined with opioids. No serious adverse events of somnolence or respiratory depression were reported. Somnolence occurred in 4 participants (0.7%) randomized to placebo and 11 participants (1.8%) randomized to gabapentin during the trial. Respiratory depression was reported in 3 participants overall: 1 in the placebo group (0.2%) and 2 in the gabapentin group (0.3%). The number of adverse events in the neurologic and respiratory Medical Dictionary for Regulatory Activities classes was broadly similar (and inconsistently distributed) between the gabapentin and placebo groups.

### Strengths and Weaknesses of the Study

The major strengths of this study are that it was pragmatic, at low risk of bias, and integrated in the existing usual care pathways for major surgery across a number of centers. It was also conducted in three major surgical specialties, ensuring that findings are generalizable to all major body cavity surgery. No other study of gabapentin in the perioperative setting has included such a wide variety of surgery types.^14,25^ Most previous studies were limited to a single specialty and, in many cases, a single operation (*e.g.*, hysterectomy, single joint replacement). Although more participants were recruited from the cardiac (500) than the thoracic (346) and abdominal (350) surgery specialties, the numbers of patients from the latter two specialties met the minimum number needed to achieve 80% power to detect the target difference in hospital length of stay in each specialty. Therefore, this imbalance in recruitment has no impact on the conclusions.

The GAP Study did not test the application of gabapentin to other major non–body cavity surgery (*e.g.*, joint replacement) or nonmajor (*e.g.*, day care) surgery. Care pathways and analgesic regimens for these other types of surgery are different, and therefore we cannot fully assess the impact of the addition of gabapentin to them. However, given the minimal impact of gabapentin on pain scores within the GAP Study, we would not anticipate that postoperative pain would be significantly improved in other settings as indicated by the most recent meta-analysis.^14^

Other limitations of the trial include the nonvariable dose of gabapentin and the restricted period of the intervention. Therefore, we cannot assess the impact of a higher gabapentin dose on pain or the impact of a reduced dose on adverse effects in vulnerable populations such as the elderly and frail. However, since the NRS at rest were less than 2 of 10 (below the acceptable pain score at rest of 3^24^), and pain scores on movement were less than 4 of 10 from 48 h after surgery, the impact of prolonged treatment with gabapentin beyond the time period assessed is likely to be limited.

### Implications for Clinicians or Policymakers

Guidance for use of gabapentin in the perioperative setting varies: Gabapentin is included as a “strong recommendation” as a component of multimodal analgesia for the management of postoperative pain in the United States,^16^ but not in Europe.^17^ The United Kingdom National Institute for Health and Care Excellence issued a “recommendation for research” for the place of gabapentin in the perioperative setting.^15^ The findings of this study, taken together with previous research,^14^ suggest that gabapentin should not be part of standard perioperative analgesic regimens for unselected patients undergoing major body cavity surgery as it provides little benefit for either patients or care providers.

### Unanswered Questions and Future Research

The GAP Study was not designed to test the place of gabapentin as “rescue” therapy for those whose pain is not controllable using conventional multimodal analgesia. The place of gabapentin in those with preexisting and persistent postsurgical pain must also be answered. Therefore, there is potential for studies to investigate the place of gabapentin in this setting. However, the differences in NRS between gabapentin and placebo at rest and on movement were small and clinically insignificant. It is therefore unlikely that it will be effective at controlling pain after major surgery, even as a rescue therapy.

### Conclusions

Among participants undergoing major cardiac, thoracic, and abdominal surgery, the addition of gabapentin to multimodal analgesic regimes did not result in a clinically important change in hospital length of stay, opioid use, acute pain, nor quality of life. Participants who took gabapentin had a higher incidence of pain at 4 months.

### Data Sharing

After publication, anonymized individual participant data will be made available upon request to the corresponding author for secondary research, conditional on assurance from the secondary researcher that the proposed use of the data is compliant with the Medical Research Council Policy on Data Sharing regarding scientific quality, ethical requirements, and value for money. Only data from participants who have consented for their data to be shared with other researchers will be provided.

### Research Support

Supported by the United Kingdom National Institute for Health Research Heath Technology Assessment (reference No. 15/101/16).

### Competing Interests

Dr. Batchelor has received speaker fees/advisory board fees from JnJ, Medtronic, and Bristol Myers Squibb. Dr. Edwards has received an honorariam for a lecture from Edwards Lifesciences. Dr. Grocott declares the following conflicts of interest: Edwards Lifesciences (consultancy/medical advisory board). Dr. Casali left employment at the institution at which the study was being conducted during the trial; he is now employed by JnJ Med Tech. The other authors declare no competing interests.

## Supplemental Digital Content

GAP Study protocol, https://links.lww.com/ALN/E126

GAP Study statistical analysis plan (SAP), https://links.lww.com/ALN/E127

GAP Study supplemental tables and acknowledgments, https://links.lww.com/ALN/E128

## Supplementary Material
