# Supplementary material for: Gabapentin for Pain Management after Major Surgery: A Placebo-controlled, Double-blinded, Randomized Clinical Trial (the GAP Study)
Source: Anesthesiology. 2025 Jul 15;143(4):851–61. doi: 10.1097/ALN.0000000000005655 (PMC12416896; doi:10.1097/ALN.0000000000005655)
Supplement: Supplementary file 1 [file aln-143-851-s001.pdf]

Effectiveness, cost effectiveness and safety of gabapentin versus placebo as an adjunct to multimodal pain regimens in surgical patients: A placebo controlled randomised controlled trial with blinding (The GAP study)

Funder ref: 15/101/16
REC ref: 17/YH/0381
Sponsorship ref: SU/2016/6033
IRAS ref: 225986
ISRCTN ref: ISRCTN63614165
EUDRACT ref: 2017-002078-38

Details of Sponsor

University Hospitals Bristol and Weston NHS Foundation Trust
Research and Innovation
Level 3, UH Bristol Education and Research Centre
Upper Maudlin Street
Bristol BS2 8AE

Tel: 0117 342 0233
Fax: 0117 342 0239

Lead applicant, Chief investigator & Research Team Contact Details

Lead applicant and non-clinical lead
Professor Chris Rogers
Co-Director
Bristol Trials Centre (Clinical Trials and Evaluation Unit) (BTC(CTEU))
University of Bristol
Bristol Royal Infirmary, Level 7 (Zone A)
Marlborough Street
Bristol BS2 8HW

Tel: 0117 342 2507
Email: chris.rogers@bristol.ac.uk

Chief investigator (CI) and clinical lead
Dr Ben Gibbison
Consultant in Cardiac Anaesthesia and Intensive Care
University Hospitals Bristol and Weston NHS Trust (UHBW)
Bristol Royal Infirmary
Marlborough Street
Bristol BS2 8HW

Tel: 07931568135
E-mail: mdbjjg@bristol.ac.uk

Methodologist
Dr Maria Pufulete
BTC(CTEU)
University of Bristol
Bristol Royal Infirmary, Level 7 (Zone A)
Marlborough Street
Bristol BS2 8HW

Tel: 0117 342 2526
Email: maria.pufulete@bristol.ac.uk

Senior trial manager
Dr Lucy Culliford
BTC(CTEU)
University of Bristol
Bristol Royal Infirmary, Level 7 (Zone A)
Marlborough Street
Bristol BS2 8HW

Tel: 0117 342 2374
Email: lucy.culliford@bristol.ac.uk

© University of Bristol, 2017

Methodologist
Professor Barnaby Reeves
Co-Director
BTC(CTEU)
University of Bristol
Bristol Royal Infirmary, Level 7 (Zone A)
Marlborough Street
Bristol BS2 8HW

Tel: 0117 342 2507
Email: barney.reeves@bristol.ac.uk

Neuropathic pain specialist
Professor David Wynick
Professor of Molecular Medicine and Consultant in Pain Medicine
Department of Translational Health Sciences
Bristol Medical School, University of Bristol

Joint Director of Research, NBT & UHBW
Research & Innovation Department
Level 3, Education and Research Centre
UHBW NHS Foundation Trust, Upper Maudlin Street, Bristol, BS2 8AE

Tel: 0117 342 0584
Email: D.Wynick@bristol.ac.uk

Pain management lead
Dr Nilesh Chauhan
Department of Anaesthesia
UHBW NHS Trust
Bristol Royal Infirmary
Marlborough Street
Bristol BS2 8HW

Tel: 0117 342 5091
Email: nilesh.chauhan@uhbw.nhs.uk

Cardiac surgeon
Professor Gianni Angelini
Bristol Heart Institute
UHBW NHS Trust
Bristol Royal Infirmary
Marlborough Street
Bristol BS2 8HW

Tel: 0117 342 3145
Email: G.D.Angelini@bristol.ac.uk

Hepatobiliary surgeon
Mr Reyad Al-Ghnaniem Abbadi
Department of Surgery
UHBW NHS Trust
Bristol Royal Infirmary
Marlborough Street
Bristol BS2 8HW

Tel: 0117 342 4595
Email: reyad@doctors.org.uk

Consultant anaesthetist
Dr Nick Goddard
University Hospital Southampton NHS Foundation Trust
Tremona Road
Southampton SO16 6YD

Tel: 023 8120 6135
Email: Nicholas.Goddard@uhs.nhs.uk

Consultant anaesthetist
Dr Mat Molyneux
UHBW NHS Trust
Bristol Royal Infirmary
Marlborough Street
Bristol BS2 8HW

Tel: 0117 342 2890
Email: mat.molyneux@uhbw.nhs.uk

Thoracic surgeon
Mr Aiman Alzetani
University Hospital Southampton NHS Foundation Trust
Tremona Road
Southampton SO16 6YD

Tel: 023 8120 5951
Email: Aiman.Alzetani@uhs.nhs.uk

Anaesthetist and PPI lead
Dr Mark Edwards
Department of Anaesthesia & Critical Care
University Hospital Southampton NHS Foundation Trust
Tremona Road
Southampton SO16 6YD

Tel: 023 8120 6135
Email: Mark.Edwards2@uhs.nhs.uk

Anaesthetist and critical care specialist
Professor Mike Grocott
Clinical and Experimental Sciences,
Faculty of Medicine
University of Southampton
University Road
Southampton SO17 1BJ

Tel: 023 8120 8449
Email: mike.grocott@soton.ac.uk

GAP study manager
Dr Sarah Baos
BTC(CTEU)
University of Bristol
Bristol Royal Infirmary, Level 7 (Zone A)
Marlborough Street
Bristol BS2 8HW

Tel: 0117 342 4195
Email: gap-study@bristol.ac.uk

Health economist
Prof Sarah Wordsworth
Nuffield Department of Population Health
University of Oxford
Richard Doll Building
Old Road Campus
Oxford OX3 7LF

Tel: 01865 289 268
Email: sarah.wordsworth@dph.ox.ac.uk

Table of contents

Glossary / abbreviations 6
1. Trial summary 7
2. Background 7
2.1 Peri-operative pain management 7
2.2 Evidence for use of gabapentin in the peri-operative setting 8
3. Rationale for the study 8
4. Aims and objectives 8
5. Plan of investigation 10
5.1 Trial schema 10
5.2 Trial design 11
5.3 Setting 11
5.4 Key design features to minimise bias 11
5.5 Trial population 12
5.6 Trial interventions 13
5.7 Primary and secondary outcomes 16
5.8 Sample size calculation 17
6. Trial methods 18
6.1 Description of randomisation 18
6.2 Code breaking 18
6.3 Blinding 18
6.4 Research procedures 19
6.5 Duration of treatment period 20
6.6 Definition of end of trial 20
6.7 Data collection 20
6.8 Source data 22
6.9 Planned recruitment rate 22
6.10 Participant recruitment 23
6.11 Discontinuation/withdrawal of participants 24
6.12 Frequency and duration of follow up 24
6.13 Likely rate of loss to follow-up 24
6.14 Expenses 25
7. Statistical analyses 25
7.1 Plan of analysis 25
7.2 Subgroup analyses 25
7.3 Frequency of analyses 25
7.4 Criteria for the termination of the trial 26
7.5 Economic evaluation 26
8. Trial management 26
8.1 Trial Oversight 26
8.2 Day-to-day management 26
8.3 Monitoring of sites 27
8.4 Trial Steering Committee and Data Monitoring and Safety Committee 27
9. Safety reporting 27
9.1 Definitions 27
9.2 Overview 28
9.3 Expected adverse events associated with the study medication 30
9.4 Anticipated events associated with surgery or adjuvant chemotherapy/radiotherapy 33
9.5 Period for recording serious adverse events 33

10. Ethical considerations ... **37**
10.1 Review by an NHS Research Ethics Committee ... 37
10.2 Risks and anticipated benefits ... 37
10.3 Informing potential study participants of possible benefits and known risks ... 37
10.4 Obtaining informed consent from participants ... 37
10.5 Co-enrolment ... 37
11. Research governance ... **38**
11.1 Sponsor approval ... 38
11.2 NHS approval ... 38
11.3 Investigators' responsibilities ... 38
11.4 Monitoring by sponsor ... 38
11.5 Indemnity ... 38
11.6 Clinical Trial Authorisation ... 39
12. Data protection and participant confidentiality ... **39**
12.1 Data protection ... 39
12.2 Data handling and storage for participant electronic consent (e-consent) data ... 39
12.3 Data handling, storage and sharing of main study data ... 39
13. Dissemination of findings ... **40**
14. References ... **41**
15. Amendments to protocol ... **43**

Glossary / abbreviations

AE	Adverse event
AR	Adverse reaction
ARDS	Acute respiratory distress syndrome
BPI	Brief pain inventory
BTC(CTEU)	Bristol Trials Centre (Clinical Trials and Evaluation Unit)
CI	Chief Investigator
CNS	Central nervous system
CPAP	Continuous positive airway pressure – a method of respiratory ventilation providing oxygen for any period of time post extubation
CRF	Case report form
CTA	Clinical trial authorisation
CT-IMP	Clinical trial of an investigational medicinal product
DMSC	Data monitoring and safety committee
DRESS	Drug rash with eosinophilia and systemic symptoms
eGFR	Estimated glomerular filtration rate: derived from gender, age, ethnicity and serum creatinine
EQ-5D-5L	EuroQol 5 dimension 5 level questionnaire
HRA	Health Research Authority
HRQoL	Health-related quality of life
HTA	Health technology assessment
GCP	Good clinical practice
ICU	Intensive Care Unit
ITT	Intention to treat
MHRA	Medicines and healthcare products regulatory agency
NIHR	National Institute for Health Research
NRS	Numerical rating score
NSAIDS	Non-steroidal anti-inflammatory drugs
PCA	Patient controlled analgesia
PI	Principal Investigator
PIL	Patient information leaflet
QALYs	quality adjusted life years
RCT	Randomised controlled trial
REC	Research ethics committee
REDCap	Research Electronic Data Capture
RSI	Reference safety information
SAE	Serious adverse event
SF-12	Short-form-12
SmPC	Summary of product characteristics
SOP	Standard operating procedure
SSA	Site Specific Assessment
SSAR	Suspected serious adverse reaction
SUSAR	Suspected unexpected serious adverse reaction
TIA	Transient ischemic attack
TMG	Trial management group
TSC	Trial steering committee
UHBW	University Hospitals Bristol and Weston NHS Foundation Trust

1. Trial summary

Gabapentin is a medicine used to treat epilepsy and pain caused by dysfunctional nerves. Recently, doctors have begun using gabapentin to treat pain after an operation, with the intention of reducing the amount of other drugs needed while maintaining good pain relief. Opioid drugs (for example morphine and fentanyl) are the most commonly used drugs to control pain after surgery, but doctors want to try to reduce the amount of opioid drugs because they cause side effects, often delaying discharge from hospital and leading to slower recovery. There is uncertainty about whether adding gabapentin to the usual drug regimen (which includes opioid drugs) will result in good pain relief, fewer side effects overall and faster recovery after surgery.

We want to conduct a randomised controlled trial (RCT), in which patients undergoing different types of surgery (heart, lungs or abdominal) are allocated by chance to receive gabapentin or an identical looking dummy pill (known as a placebo) in addition to the usual painkillers for each type of surgery. We will give patients gabapentin or placebo one hour before surgery and for two days after surgery. Every other aspect of care will stay the same. We will then compare patients who received gabapentin with those who received the placebo to determine whether gabapentin reduces the amount of time patients stay in hospital after the operation, the amount of opioid medication they take, and to assess how gabapentin influences pain in hospital and four months after surgery. We will also perform a health economic analysis to determine whether using gabapentin provides better health outcomes for patients undergoing surgery and is cost-effective for the NHS relative to current standard care.

We have chosen to conduct the study in patients undergoing different types of major surgery, rather than just one. We are using this approach because we want to ensure that the results can be applied to patients undergoing different operations and who receive different types of care. We will conduct the trial in two phases. In phase 1, we will determine whether we can recruit patients from the three different types of surgery and whether patients are able to take gabapentin or placebo as instructed. If successful, we will move on to phase 2 and continue recruiting until we recruit about 1180 patients (approximately 340 as a minimum from each type of surgery).

2. Background

2.1 Peri-operative pain management

In the UK each year, about 4.7 million patients undergo surgery [1]. Many of these patients experience pain after surgery and about 10% experience severe pain [2, 3]. Inadequate pain management increases length of hospital stay [4], and contributes to the development of chronic or persistent post-surgical pain [5, 6], which impacts on quality of life [7]. Current multimodal analgesic regimens include paracetamol, non-steroidal anti-inflammatory drugs (NSAIDS) and opioids.

Opioids are the key analgesic agents for managing moderate to severe pain. However, they have poor efficacy in movement-associated pain and side effects including confusion, nausea, vomiting, itching, constipation and respiratory depression. Opioid side effects increase the length of hospital stay, delay overall recovery and impact on quality of life [7]. Reliance on opioids after surgery also increases the risk of opioid dependence and long-term use [8, 9].

2.2 Evidence for use of gabapentin in the peri-operative setting

Doctors add gabapentin to multimodal analgesic regimens to try and reduce opioid use while still controlling pain efficiently after surgery, although there is large variation in practice across the UK. Gabapentin is used “off label” in the peri-operative setting, since it is currently only licensed to treat epileptic convulsions and neuropathic pain. Gabapentin is thought to work by binding to calcium channels and reducing calcium influx into nerve cells; this mode of action is responsible for its anti-epileptic, anxiolytic, sedative and analgesic effects.

There are over 130 RCTs that have investigated gabapentin versus placebo in different surgical populations. Most of these RCTs are small (<200 patients, median 80) and highly heterogeneous (statistically and clinically). None has assessed the impact on length of hospital stay or quality of life. These RCTs have been included in 18 systematic reviews that aimed to assess the effectiveness of gabapentin vs. placebo in the peri-operative period. Eleven of these reviews included all surgical populations (one of these was restricted to studies administering a single dose of gabapentin [10]) and nine reviews included only single surgical populations (2 head and neck, 3 musculoskeletal , 1 spinal lumbar surgery, 2 gynaecology, 1 cardiac) [11-20]. All reviews included RCTs irrespective of when gabapentin was administered (before surgery, after surgery, or both). All reviews reached the same conclusions – that gabapentin reduced opioid consumption and post-operative pain scores at 24 hours ($P<0.001$) but none has assessed the impact on quality of life. The most recent systematic review was published since this study started [20] and assessed the impact of gabapentin on length of hospital stay in 8 trials which provided very low to moderate quality evidence and found no statistically significant difference in the length of hospital stay between the gabapentin and control group. It is difficult to reach conclusions about the optimal dose and duration of treatment because of the heterogeneity of the trials, although the systematic reviews suggest at least 600mg as a starting dose pre-operatively and no less than 300mg/d post-operatively to show a reduction in opioid use. There are no new or ongoing RCTs investigating gabapentin and its impact on post-surgery pain and length of hospital stay that we are aware of.

3. Rationale for the study

Optimal analgesia is critical for both patients and healthcare systems. Optimal analgesia improves patient experience and allows patients to get out of bed faster. This leads to more rapid discharge and thus improved efficiency and flow to the healthcare system. Reducing opioid use after surgery is a priority for both doctors and patients and is one of the central tenets of enhanced recovery [21]. However, the current evidence base is not robust enough to allow for definitive evidence-based national guidelines on the use of gabapentin in the peri-operative setting. The study research team consulted several patient groups (including patients with different conditions and types of surgery) to get feedback about the importance of the proposed study. There was unanimous support for the study; most patients had experienced negative side effects from opioid use (some severe) and all welcomed any pain medication that would reduce the amount of morphine patients need after surgery. Furthermore, the study team conducted a UK-wide survey of acute pain units and individual consultant anaesthetists, which showed large variation in the prescribing practice of gabapentin-type drugs.

4. Aims and objectives

The GAP study will compare the effectiveness, cost-effectiveness and safety of gabapentin as an adjunct to standard multimodal analgesia versus placebo for the management of pain following three types of major surgery (cardiac, thoracic and abdominal). The hypothesis to be tested is that gabapentin reduces opioid use after surgery and speeds up recovery, thereby reducing post-operative hospital stay compared to standard multimodal analgesia (usual care). Specific objectives are to estimate:

A. The difference between groups in the average length of hospital stay following surgery.

B. The difference between groups with respect to a range of secondary outcomes including assessment of efficacy (total opioid use, pain), measures of safety (adverse health events) and health-related quality of life (HRQoL) in the four months following randomisation.

C. The cost effectiveness of gabapentin compared to usual care.

5. Plan of investigation

5.1 Trial schema

Figure 1 Trial schema

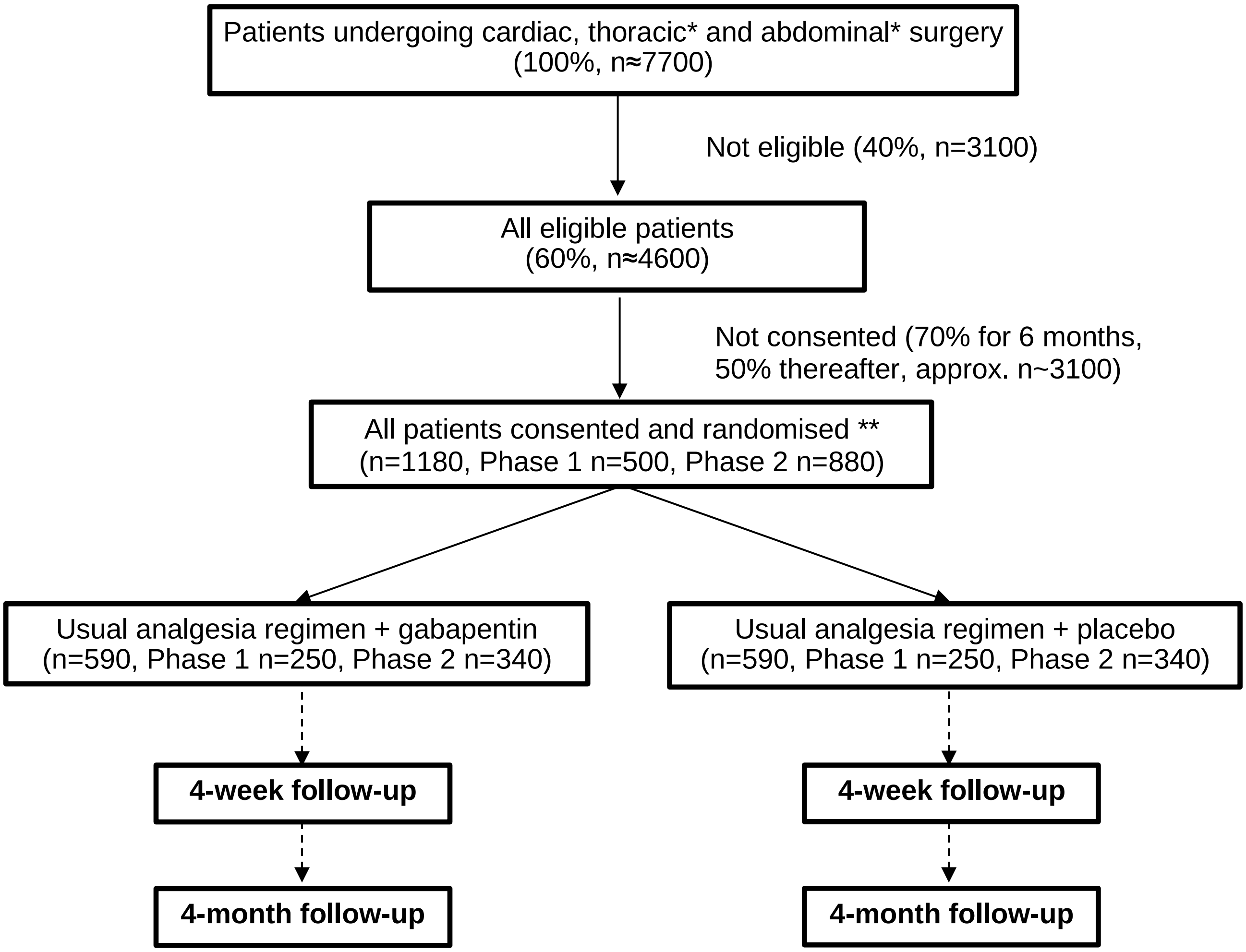

* Thoracic and abdominal surgery includes open or minimal access surgery

** Study designed with an internal pilot (progression from Phase 1 to Phase 2 dependent on achieving adequate recruitment).

5.2 Trial design

Multi-centre, parallel group, placebo-controlled, pragmatic RCT in which participants, clinical care teams and all members of the research team are blinded to allocation.

Phase 1: Set-up and recruit in three surgical specialties in two centres with integrated monitoring and feedback to maximise recruitment and adherence with the study medication.

Phase 2: Continue recruitment (opening additional centres/specialties if required), using the optimum methods of recruitment and adherence established in Phase 1 for a further 40 months and following all participants (including those recruited during Phase 1) up to 4 months.

Progression will be contingent on meeting the criteria defined in section 6.9.3.

5.3 Setting

Participants will be recruited from three surgical specialties in multiple NHS secondary care centres. A clinical lead will be identified for each specialty and principal investigator (PI) will be appointed in each centre.

5.4 Key design features to minimise bias

(a) **Bias arising from the randomisation process (selection/allocation bias)** (systematic differences between baseline characteristics of the groups that are compared) will be prevented by concealed randomisation. The allocation will be stratified by centre and specialty to minimise confounding due to these factors (see section 6.1).

(b) **Bias due to deviations from intended interventions (performance bias)** (systematic differences between groups in the care that is provided, or in exposure to factors other than the interventions of interest) will be minimised by blinding all participants, clinicians and other hospital staff caring for participants and members of the research team (apart from the study statistician) to participants' allocation. The success of blinding will be assessed (participants and their care team may become unblinded due to side effects). Performance bias will also be minimised by administering the medication (gabapentin or placebo) and other procedures undertaken during the trial according to standard protocols and by pre-defining all procedures for participant follow-up and applying the procedures to all participants in the same way. Adherence to all aspects of the protocol will be monitored (for further details see section 6.3 and 8.2).

(c) **Bias in measurement of the outcome (detection bias)** (systematic differences between groups in how outcomes are determined) will be minimised by blinding all individuals assessing outcomes, assessing the success of blinding and providing clear unambiguous definitions for each outcome measure (see section 5.7).

(d) **Bias due to missing outcome data (attrition bias)** (systematic differences between groups in withdrawals from a study) will be minimised by i) maintaining contact with participants throughout the duration of the trial to maximise the proportion of participants for whom all outcome data are available and the proportion of participants who receive the medication to which they were allocated, ii) implementing measures to promote adherence (e.g. stickers on participant records to remind the care team that participants are in the trial) and iii) documenting non-adherence to the allocated treatment (see

section 5.6.3). The data will also be analysed by intention to treat. In estimating the target sample size, we have not allowed for loss to follow-up as the primary outcome is time to hospital discharge and the follow-up period is short (4 months). However, we will pay particular attention to keeping in touch with participants and maximising retention up to 4 months.

(e) Bias in selection of the reported result (reporting bias) will be minimised by having pre-specified outcomes (see section 5.7) and a pre-specified analysis plan (section 7).

5.5 Trial population

5.5.1 Inclusion criteria

Participant may enter study if ALL of the following apply:

1. Over 18 years of age;
2. Undergoing non-emergency surgery:
   - Cardiac (surgery on the heart and great vessels carried out via midline sternotomy);
   - Thoracic surgery (open or minimal access surgery on the lungs and surrounding tissues);
   - Abdominal (open or minimal access surgery within the abdominal cavity);
3. Expected to stay in hospital at least until day 2 after surgery (day 0 is day of surgery);
4. Expected to be able to swallow during the time of the study intervention.

5.5.2 Exclusion criteria

Participants may not enter study (i.e. may not be randomised) if ANY of the following apply:

1. Taking anti-epileptic medication(s);
2. Allergy to gabapentin;
3. Already taking gabapentin or gabapentanoids;
4. Rare hereditary problems of galactose intolerance, the Lapp lactase deficiency or glucose galactose malabsorption;
5. Planned epidural analgesia;
6. Intended use of any gabapentanoids in the peri-operative analgesic protocol other than the study medication (this includes but is not restricted to: pregabalin, enacarbil gabapentin, 4-methylpregabalin and phenibut);
7. Known renal impairment (for such patients, estimated glomerular filtration rate (eGFR) <30ml/min/1.73^2);
8. Weight <50kg;
9. Inability to provide written informed consent to participate in the trial;
10. Unwilling to participate in follow-up;
11. Prisoners;
12. Enrolled in another clinical trial and: a) the patient is currently taking an investigational medicinal product as part of the other trial; or b) co-enrolment is not permitted by the other trial; or c) co-enrolment would be burdensome for the patient.

5.6 Trial interventions

The trial intervention is gabapentin 600 mg given preoperatively (ideally with the patient's premedication) and 600 mg/day (300 mg in the morning and 300 mg in the evening) given postoperatively for 2 days when clinically able to swallow following extubation (if applicable, see below) within the multimodal analgesic regimens specified by local analgesic protocols. Expert opinion and data from a systematic review (incorporating over 100 studies) was sought when establishing the study drug regimen. The control is a placebo which will be taken at the same time points as the active tablet within the multimodal analgesic regimens specified by local analgesic protocols.

If the pre-operative dose is administered and then the **surgery is postponed by more than 12 hours**, a second pre-operative dose of 600 mg of gabapentin should be given pre-operatively (ideally with the patient's premedication) for the re-scheduled surgery (see section 5.6.1).

If a post-operative dose of study medication is **missed by less than 6 hours**, patients should be given the missed dose and continue to the next scheduled dose as per the protocol.

If a dose of study medication is **missed by more than 6 hours**, patients should continue to the next scheduled dose and should not be given the missed dose of study medication.

For patients **intubated for longer than 48 hours** after the end of the operation, none of the post-operative study medication should be administered.

5.6.1 Study drug

The study drug, gabapentin, is licensed to treat epileptic convulsions and neuropathic pain. In this trial, it will be used ''off label'' as part of the multimodal analgesic regimen.

The study drug will be made available as gabapentin 300 mg hard capsule, which will be over encapsulated to achieve blinding. The placebo will use the same capsule to maintain the blinding.

Study medication will be stored and dispensed by the trial site's pharmacy department in accordance with Good Clinical Practice, Good Manufacturing Practice and pharmacy department SOPs. The encapsulated study medication will be packaged into a numbered pack, prepared by the study pharmacy. Study participants will be allocated a unique study medication pack number. Each pack will contain 6 capsules of study medication (either gabapentin or placebo). If surgery is either postponed by more than 12 hours, or cancelled and re-arranged for a later date, after administration of the pre-operative 600 mg dose, a second study medication pack, with a new unique study medication pack number, will be allocated to the study participant. This second study medication pack will contain 6 capsules of the same study medication as the first pack (i.e. both packs will contain gabapentin or both will contain placebo). The first study medication pack containing 4 unused capsules will be returned to the local pharmacy for accountability and disposal by approved means.

The manufacture, quality assurance, labelling and packaging is described in the IMP dossier.

5.6.2 Storage

The medicinal product should be stored at room temperature not above 25ºC.

5.6.3 Compliance

Compliance will be monitored by recording administration of the study drug on the case report form (CRF) and study database, including the pack number so that it will be possible to check that the correct dose treatment has been given. Used study drug packs will be returned to the local pharmacy for accountability.

5.6.4 Reference safety information

The Reference Safety Information (RSI) for the study is the study protocol (see section 9 for the expected adverse events associated with gabapentin). The list of expected adverse events is based on the Summary of Product Characteristics (SmPC) for gabapentin (see section 9.3). If the SmPC is updated during the course of the study, the study team will monitor and review the changes and consider the impact on the study and revise the RSI as required, in consultation with the Sponsor. Any changes to the RSI will be submitted to the Medicines and Healthcare Products Regulatory Agency (MHRA) for approval before being implemented.

5.6.5 Concomitant medication

The following interactions with gabapentin have been observed and should be taken into consideration when enrolling a patient and during the period of study drug administration.

Opioids: There are spontaneous and literature case reports of respiratory depression and/or sedation associated with gabapentin and opioid use. Patients who require concomitant treatment with opioids should be carefully observed for signs of central nervous system (CNS) depression, such as somnolence, sedation and respiratory depression and the dose of opioid should be reduced appropriately. This applies particularly to elderly patients.

Antacids containing aluminium and magnesium: These products reduce gabapentin bioavailability up to 24%; therefore, it is recommended that antacid medication is not taken in the two hours prior to planned administration of the study medication.

Cimetidine: A slight decrease in renal excretion of gabapentin is observed when it is co-administered with cimetidine but this is not expected to be of clinical importance.

Naproxen: Co-administration of naproxen sodium capsules (250 mg) with gabapentin (125 mg) appears to increase the amount of gabapentin absorbed by 12% to 15%. These doses are lower than the therapeutic doses for both drugs. The magnitude of interaction within the recommended dose ranges of either drug is not known. This is unlikely to be an issue in the trial since naproxen is not administered peri-operatively as part of analgesic protocols.

No interaction between gabapentin and phenobarbital, phenytoin, valproic acid, or carbamazepine has been observed.

5.6.6 Special warnings and precautions for use

The information below is detailed in the SmPC for gabapentin [22]. However, the events listed below have only been observed in patients who take gabapentin for the management of epilepsy and peripheral neuropathic pain (i.e. long term use), therefore it is unlikely that these

events will occur in the GAP study population who are administered gabapentin for a short period of time.

Suicidal ideation and behaviour: Suicidal ideation and behaviour have been reported in patients treated with antiepileptic agents in several indications. A meta-analysis of randomised placebo-controlled trials of antiepileptic drugs has also shown a small increased risk of suicidal ideation and behaviour. The mechanism of this risk is not known and the available data do not exclude the possibility of an increased risk for gabapentin. Therefore, patients should be monitored for signs of suicidal ideation and behaviours and appropriate treatment should be considered. Patients (and caregivers of patients) will be advised to seek medical advice should signs of suicidal ideation or behaviour emerge.

Anaphylaxis: Gabapentin can cause anaphylaxis. Signs and symptoms in reported cases have included difficulty breathing, swelling of the lips, throat, and tongue, and hypotension requiring emergency treatment. Study medication will be discontinued and medical care provided if patients experience signs or symptoms of anaphylaxis.

Acute pancreatitis: Although acute pancreatitis has been reported in patients treated with gabapentin for epilepsy, causality with gabapentin is not clear. In the unlikely event that a patient develops acute pancreatitis during the trial, discontinuation of the study medication will be considered.

Seizures: Gabapentin is not considered effective against primary generalized seizures such as absences and may aggravate these seizures in some patients. Therefore, gabapentin should be used with caution in patients with mixed seizures including absences. This is not relevant to our trial since patients with epilepsy (who are on anti-epileptic medications) will be excluded. Gabapentin treatment has been associated with dizziness and somnolence, which could increase the occurrence of accidental injury (fall). There have also been post-marketing reports of loss of consciousness, confusion and mental impairment. Patients in the trial will be monitored closely for these side effects.

Use in elderly patients (over 65 years of age): No systematic studies in patients 65 years or older have been conducted with gabapentin. In one double-blind study in patients with neuropathic pain, somnolence, peripheral oedema and asthenia occurred in a somewhat higher percentage in patients aged 65 years or above, than in younger patients. Apart from these findings, clinical investigations in this age group do not indicate an adverse event profile different from that observed in younger patients.

Drug Rash with Eosinophilia and Systemic Symptoms (DRESS): Severe, life threatening, systemic hypersensitivity reactions such as drug rash with eosinophilia and systemic symptoms have been reported in patients taking antiepileptic drugs including gabapentin. It is important to note that early manifestations of hypersensitivity, such as fever or lymphadenopathy, may be present even though rash is not evident. If such signs or symptoms are present, the patient will be evaluated immediately and study medication will be discontinued if an alternative aetiology for the signs or symptoms cannot be established.

Respiratory depression

Gabapentin has been associated with severe respiratory depression. Patients with compromised respiratory function, respiratory or neurological disease, renal impairment, concomitant use of CNS depressants and the elderly might be at higher risk of experiencing this severe adverse reaction. Dose adjustments might be necessary in these patients.

5.6.7 Peri-operative analgesic protocols

The peri-operative analgesic protocols will follow local policy. Use of any gabapentanoids other than the study medication is prohibited, this includes but is not restricted to: pregabalin, enacarbil gabapentin, 4-methylpregabalin and phenibut. We will collect data on the analgesia used for each patient.

5.6.8 Surgery

All surgical procedures will be carried out according to local protocols and at the discretion of the surgeons involved.

5.6.9 Clinical care of participants

Participants will only receive the study drug whilst they are an in-patient. Participants will be subject to high levels of clinical care as part of their peri-operative management. In the operating theatre they are continuously monitored by one anaesthetist. Whilst on the Intensive Care Unit (ICU) they will receive continuous vital sign monitoring via routinely placed indwelling intravascular catheters at a nursing ratio of 1 nurse to every 1 or 2 patients, as required by clinical dependency. On the normal ward they will be subject to a minimum of 4 hourly vital sign monitoring and more frequently when clinically required (up to every 5 minutes in the post-anaesthetic care unit). Monitoring will not be introduced specifically for the trial as this would not be as intensive or rigorous as the routine care participants will already be receiving, and would undermine the pragmatic nature of this study.

5.6.10 Concomitant interventions

Concomitant interventions (co-interventions) are interventions that naturally accompany the surgical procedure, and can include pre-operative, peri-operative (e.g. general anaesthesia protocols) and post-operative (e.g. management in intensive care and high dependency units) components. All co-interventions will be carried out according to local protocols. Although we will collect information on standard protocols for peri-operative care, it is unlikely that co-interventions will introduce the risk of performance bias as the trial is blinded.

5.7 Primary and secondary outcomes

5.7.1 Primary outcome

The primary outcome is the time from start of surgery to hospital discharge. Post-operative hospital stay has been chosen because it will reflect the anticipated earlier recovery with gabapentin.

5.7.2 Secondary outcomes

Secondary outcomes have been selected to assess the efficacy and safety of gabapentin and include:

(a) Opioid consumption in the period from surgery until hospital discharge;
(b) Opioid consumption from discharge until 4 months;

(c) Acute post-operative pain assessed using the numerical rating scale (NRS) completed at 1 hr, 4 hr, 12 hr post-surgery and then twice daily to discharge;

(d) Adverse health events from randomisation to discharge and serious adverse events up to 4 months;

(e) HRQoL measured using the EQ-5D 5 level questionnaire and Short-form (SF) 12 completed at baseline and at follow-up at approximately 4 weeks and 4 months;

(f) Resource use to 4 months (measured during the hospital stay, at 4 weeks and 4 months) (see section 7.5);

(g) Pain measured at baseline, at 4 weeks and at 4 months using the brief pain inventory (BPI).

5.8 Sample size calculation

The study hypothesis is that, on average, the post-operative hospital stay for participants' allocated gabapentin will be shorter than the post-operative hospital stay for participants allocated the placebo medication. The sample size has been chosen to test this hypothesis. In estimating the sample size, we have considered the proportion of patients discharged at the current median time (5 days for cardiac and abdominal surgery, 3 days for thoracic surgery). The target difference is expressed in terms of the increase in the proportion of patients discharged by this time that would persuade clinicians to change practice. The clinicians on the study team have informed these discussions. The sample size required to achieve 80% and 90% power, assuming 5% statistical significance and a two-sided test, for target differences of 15%, 12.5% and 10% are shown in Table 1. The calculations assume a constant hazard ratio for the time to discharge (shown in Table 1) and allow for 5% censoring (i.e. deaths prior to discharge).

The study size was set at 1500 in total, with a minimum of 376 per surgical stratum. This would provide at least 90% power to detect a difference of 12.5% in each stratum and 80% power to detect a difference of 10% in any surgical strata recruiting in excess of 430 participants.

Following minimal recruitment due to the COVID-19 pandemic in 2020/2021 the sample size was reviewed by the Trial Steering Committee and the funder. At this stage in the trial recruitment to the cardiac stratum was complete (500 participants randomised) but the thoracic and abdominal strata were incomplete. It was decided that the power of the study should be reduced to 80%, i.e. a minimum of 280 participants per stratum, which after adjusting for the observed non-compliance rate of 27% in the thoracic and abdominal specialties, was increased to 340 participants, giving a revised total sample of 1180 participants (500 cardiac, 340 thoracic, 340 abdominal).

Table 1 Proportion being discharged by current median time to discharge

Proportion being discharged by current median time to discharge		Hazard ratio	Sample size (total per stratum) Power	
Placebo	Gabapentin		90%	80%
0.50	0.65	1.51	262	196
0.50	0.625	1.41	**376**	280
0.50	0.60	1.32	574	430

6. Trial methods

6.1 Description of randomisation

Randomisation will be carried out as close to the planned operation as possible, after eligibility has been confirmed and consent given. Randomisation will be performed by an authorised member of the local research team using a secure internet-based randomisation system ensuring allocation concealment. Participants will be allocated in a 1:1 ratio to either i) gabapentin or ii) placebo. The allocation will be computer-generated. The random allocation to gabapentin or placebo will be stratified by centre and specialty, so that each specialty at each centre will have approximately equal numbers of participants allocated to placebo and gabapentin. Only a unique pack number will be revealed by the randomisation system to maintain blinding.

6.2 Code breaking

Code breaking may be necessary in an emergency (e.g. suspected serious adverse reaction, SSAR, see section 9.1 for definitions) when knowledge of what medications the participant has received will affect how they will be treated.

During office hours, the Bristol Trials Centre (Clinical Trials and Evaluation Unit) (BTC (CTEU)) should be contacted for requests to unblind. Out of office hours the clinical lead should be contacted. Unblinding should be only be performed in the event of a serious adverse event (SAE) where knowledge of the study treatment will influence ongoing clinical care. Unblinding should be approved by the clinical lead (or in the event of the clinical lead being unavailable, the study pain management lead) for the trial. If neither are available unblinding can occur in an emergency situation by contacting the Bristol pharmacy on-call pharmacist using their on-call system. Any request for unblinding will be fully documented, including information about who requested the unblinding and the reason for unblinding. Unblinding rates will be monitored throughout the trial.

6.3 Blinding

Participants, their clinical care team (i.e. their surgeon, anaesthetist, and those responsible for their post-operative care) and the research nurse(s) responsible for participant follow-up, will not be informed of the allocation. The randomisation system will provide a unique code that will be used to identify the study medication to be given.

The capsules containing gabapentin or placebo do not have a particularly strong or unusual smell or taste, so we do not anticipate unblinding will occur due to the characteristics of the drug.

Gabapentin capsules will be over-encapsulated so that the capsules for active drug and placebo will look identical. We are aware that gabapentin may induce side-effects in some patients that may inadvertently unblind participants. However, given that the side effects of gabapentin (e.g. drowsiness, dizziness and difficulty concentrating) are similar to those of morphine, and that patients are likely to view side effects as resulting from their whole experience (surgery and post-operative care) it is unlikely that any patient will definitively be able to attribute a specific side effect to gabapentin.

The patient information leaflet (PIL) and the process of informed consent will explain the uncertainty around the potential beneficial effects of gabapentin over a placebo. Therefore, in the event of inadvertent unblinding of a participant, the participant should not have a strong expectation that one or other method should lead to a more favourable outcome.

Participants will be made aware before entering the study that they will not be told which treatment they will receive. Doctors will prescribe the 'study medication' rather than specifically gabapentin or placebo (see section 6.1). The unique code provided by the randomisation system will provide the medication as specified according to the pre-determined randomisation list drawn up by the study statistician prior to recruitment. The allocations will only be known by pharmacy and the study statistician and will not be disclosed to any other member of the research team.

If clinically indicated (i.e. in the event of a SSAR, the management of which might be altered by knowledge of the allocation) the treatment allocation will be unblinded (see section 6.2).

6.4 Research procedures

6.4.1 Research assessments

Participants will have a baseline assessment prior to randomisation. Participants will be followed post-operatively to discharge, at approximately 4 weeks and at 4 months post randomisation. Total peri-operative cumulative opioid consumption in morphine equivalents will be recorded (http://fpm.anzca.edu.au/documents/opioid-dose-equivalence.pdf).

Baseline HRQoL and pain questionnaires will be administered prior to randomisation. At approximately 4 weeks and 4 months the participant will be followed up via completion of a postal or online questionnaire and/or by telephone (research team will contact patients at mutually agreed times) to ascertain serious adverse events, resource use, quality of life, and ongoing pain.

6.4.2 Assessment of patient reported outcomes

Generic HRQoL will assess the profile of surgical patients in the early post-operative phase. The extensively validated SF-12 and EQ-5D-5L will assess generic aspects of health (https://www.optum.com/optum-outcomes.html and http://www.euroqol.org/home.html). The EQ-5D-5L will be used in the analysis of quality adjusted life years (QALYs).

Persistent post-surgical pain affects up to 50% of thoracotomy, cardiac and abdominal surgery patients [8, 23, 24]. There is uncertainty about the effects of gabapentin on persistent post-surgical pain. For this reason, we propose to follow up all patients for 4 months to assess this effect and to capture any post-discharge adverse events. Pain pre- and post-operatively will be assessed using the NRS and the BPI.

Reasons for non-completion of any assessment will be recorded and coded, where possible. Missing items or errors on the questionnaire will be dealt with according to the scoring manual or via imputation methods. Adherence rates will be reported in results, including the numbers of patients who have withdrawn from the study, have been lost to follow up or died. Causes of death for patients who die will be recorded.

6.5 Duration of treatment period

Participants will receive (within the multimodal analgesic regimen for each surgical group in each centre):

(a) 600 mg gabapentin (or placebo) approximately one hour before surgery;

(b) 600 mg/day (300mg in the morning and 300mg in the evening) gabapentin (or placebo) postoperatively for 2 days.

6.6 Definition of end of trial

The trial will end for a participant after they have completed follow up at 4-month post randomisation. The end of the trial as a whole will be after all trial participants have completed follow up, all data queries have been resolved, the database locked and the analysis completed.

6.7 Data collection

Each patient will be assigned a unique study number. All data recorded on paper relating to the participant will be located in CRF folders, which will be stored securely. Staff with authorisation to make changes to the study records, including the study database, will be listed on the study delegation log maintained at each specialty/centre. The baseline data will be collected after consent. Consenting patients will be contacted by an authorised member of the local research team (as specified in the delegation log) who will answer any questions, confirm the patient's eligibility and take written informed consent if the patient decides to participate. Patients who choose to consent using electronic consent methods will provide their email address to the local research team to receive a link to the electronic consent form. Eligibility must be confirmed by a clinician. In accordance with the Sponsor's Standard Operating Procedure on 'Extended Roles of Non-medical Clinicians (SOP-022)', study eligibility may also be confirmed by non-medical clinicians (e.g. advanced nurse practitioners or clinical nurse specialists) who are appropriately trained and qualified. Data collection will include the following elements:

(a) A screening log of all non-emergency patients referred for cardiac, thoracic and abdominal surgery, and those who are approached for the trial (including the date when they are given the PIL);

(b) Patients approached and assessed against the eligibility criteria and, if ineligible, reasons for ineligibility;

(c) Consent information collected prior to randomisation in all participating patients.

(d) Baseline information (e.g. history and planned operation and response to health status questionnaires) collected in all participating patients;

(e) Data relating to the participant's surgery and hospital stay (including opioid consumption and adverse events) collected in all participating patients;

(f) Study medication prescribed for all participating patients;

(g) Data on acute post-operative pain (measured via NRS), persistent post-surgical pain (measured via the BPI), adverse events and health status questionnaires collected during the hospital stay, at 4 weeks and at 4 months for all participating patients.

To minimise bias, outcome measures are defined as far as possible on the basis of objective criteria and all personnel carrying out outcome assessment will be blinded.

Table 2 Data collection

Data item	Pre-randomisation	Pre-surgery	Intra-operative	Post-surgery (until discharge)	Discharge	4 weeks	4 months
Socio-demographic details	✓						
Co-morbidities	✓						
Routine clinical measures	✓				✓		
Resource use schedule					✓	✓	✓
SF-12	✓					✓	✓
EQ-5D-5L	✓					✓	✓
NRS pain score	✓*			✓*	✓		
Study medication		✓		✓**			
Opioid use	✓		✓	✓	✓	✓	✓
Adverse events				✓	✓		
Serious adverse events				✓	✓	✓	✓
BPI	✓					✓	✓

* Routinely collected NRS pain scores as close as possible to the following time points will be used: pre-randomisation, 1 hr, 4 hrs, 12 hrs post-surgery and twice daily post-surgery until discharge. NRS pain assessments will not be possible in intubated patients.
** Study medication given morning and evening for 2 days following extubation (where applicable).

6.8 Source data

The primary data source will be the participant's medical notes, alongside the data collection forms for the study. Pain scores reported using the NRS will be the primary data source for calculating pain scores. The completed patient questionnaires will be the primary data source for HRQoL and persistent post-surgical pain.

6.9 Planned recruitment rate

6.9.1 Phase 1

Phase 1 recruitment will take place over 12 months, starting at the first "site" (i.e. one specialty within a centre), with other sites following on (our recruitment estimates allow for staggered start dates by specialty within a centre). There will be a review of the progression criteria after 9 months.

6.9.2 Phase 2

If the progression criteria are met, recruitment into Phase 2 will continue for a further 40 months. All participants (including those recruited in Phase 1) will be followed up for 4 months.

6.9.3 Continuation/stopping rules to proceed to Phase 2

The trial will continue into Phase 2 if it is possible to demonstrate that, after 9 months of recruitment in Phase 1, sufficient numbers of patients referred for surgery are eligible for the trial and can be enrolled to complete the main trial over the remaining 51 months (a further 3 months recruiting to phase 1; 40 months recruiting to phase 2; 4 months follow up; 4 months analysis and reporting). Specifically:

(a) at least 60% of patients undergoing surgery are considered eligible for the trial (if necessary, by revising the eligibility criteria);

(b) at least 50% of eligible patients consent to randomisation by 6 months of recruitment (i.e. each specialty/centre achieves a 50% consent rate in their 6th month of recruitment).

Table 3 shows projected recruitment figures based on these assumptions and an increasing recruitment rate over time, if the trial recruits from the two phase 1 centres (Bristol and Southampton) only.

Table 3 Potential recruitment in participating surgical specialties and centres, assuming that 60% of patients undergoing surgery are eligible for the trial

Specialty/ Centre	No. of procedures/ year	No./year if 30% of eligible patients recruited	No./year if 50% of eligible patients recruited	Total if 30% of eligible patients recruited up to 6 months & 50% thereafter*		
				Phase 1 (12 mths)	Phase 2 (18 mths)	Total
**Thoracic**						
Bristol	450	81	135	90	169	259
Southampton	200	36	60	40	75	115
**Cardiac**						
Bristol	800	144	240	160	300	460
Southampton	900	162	270	180	338	518
**Abdominal**						
Bristol	570	103	171	114	214	328
Southampton	400	72	120	80	150	230
**Total**	**3320**	**598**	**996**	**664**	**1246**	**1910**

** These figures assume all specialities/centres start recruitment at the same time, at the start of the recruitment period; targets will be adjusted once the actual start date of each specialty/ centre is known. Figures also assume that for each 12-month period, the equivalent of 10-months recruitment is achieved (averaged over the period). They also assume the recruitment rate is 30% in months 1-6 and 50% thereafter.*

6.10 Participant recruitment

Patients undergoing non-emergency cardiac (surgery on the heart and great vessels carried out via a midline sternotomy); thoracic surgery (open or minimal access surgery on the lungs and surrounding tissues); abdominal surgery (open or minimal access surgery within the abdominal cavity) will be invited to participate. Potential trial participants will be identified by local teams.

All potential participants will be sent or given an invitation letter and PIL (approved by the local Research Ethics Committee, REC) describing the study. The patient will have time to read the PIL and to discuss their participation with others outside the research team (e.g. relatives or friends) if they wish. Most patients will have at least 24 hours to consider whether to participate, however as it will be important to include urgent non-emergency patients who may have had less than 24 hours to consider the study. In these circumstances, patients will only be enrolled if they confirm that they have had enough time to consider their participation.

Before their planned operation, patients will speak to a member of the local research team (e.g. study clinician/research nurse/trial co-ordinator) who will answer any questions, confirm the patient's eligibility and take written informed consent if the patient decides to participate. Consent can be obtained using several methods; either face to face (at a clinic appointment or when the patient attends hospital), or remotely by telephone/video call, or electronically using a purposely designed electronic database. Details of all patients approached for the trial and reason(s) for non-participation (e.g. reason for being ineligible or patient refusal) will be documented. The participants' General Practitioners will be informed of their enrolment in the study.

6.11 Discontinuation/withdrawal of participants

Each participant has the right to withdraw at any time. It is unlikely for this trial that there would be any reason for the investigator to withdraw the participant from their allocated treatment (especially as the investigator is blinded to participant allocation), unless subsequent to randomisation, a clinical reason for not performing the surgical procedure is discovered.

The study intervention may be stopped early if the patient experiences an SAE that the treating clinician thinks may be attributable to the study intervention or may get worse if study intervention is continued.

Participants withdrawn from their allocated intervention but willing to continue completing follow-up schedules will be encouraged to do so. All discontinuations and withdrawals will be documented. If a participant wishes to discontinue, data collected up until that point will be included in the analyses, unless the participant expresses a wish for their data to not be used.

6.12 Frequency and duration of follow up

Data for the primary outcome and most secondary outcomes will be collected during hospital stay. Patients will be followed up at approximately 4 weeks and at 4 months for information on pain, adverse events, resource use and quality of life. Patients will be offered an optional patient diary on discharge, which can be used as an aide memoire to record information during follow up if they wish.

6.13 Likely rate of loss to follow-up

Loss to follow-up for the primary and most secondary outcomes is likely to be low because data will be collected during the hospital stay. Established BTC(CTEU) methods will be used to maximise the proportion of participants for whom all outcome data are available and the proportion of participants who receive the intervention to which they were allocated. After discharge from hospital, the only losses to follow-up will be due to death or participant discontinuation. We expect loss to follow-up after discharge over the 4 months of follow-up to be less than 5%.

6.14 Expenses

There will be no ‘research only’ visits, as follow up data collection will occur via phone, online or postal questionnaires, therefore participant travel expenses are not required.

7. Statistical analyses

7.1 Plan of analysis

The data will be analysed according to intention to treat (ITT) and follow CONSORT reporting guidelines. Randomised participants who fail to complete the course of treatment (for example, due to unplanned early discharge) will be included in the primary analysis. Analyses will be adjusted/stratified (as appropriate) for centre and specialty.

Hospital stay will be compared using survival methods, allowing for censoring of any participant who dies prior to hospital discharge. HRQoL, pain scores and opioid use will be compared using a mixed regression model, adjusted for baseline measures where appropriate. Changes in treatment effect with time will be assessed by adding a treatment x time interaction to the model and comparing models again using a likelihood ratio test. Deaths will be accounted for by modelling HRQoL and survival jointly. Model fit will be assessed and alternative models and/or transformations (e.g. to induce normality) will be explored where appropriate. Treatment differences will be reported with 95% confidence intervals.

Frequencies of adverse events will be described, and the numbers of participants experiencing one or more SAE will be compared. A detailed analysis plan will be prepared. Interim analyses will be decided in discussion with the Data Monitoring and Safety Committee (DMSC). There is no intention to compare any outcomes between groups after the completion of Phase 1; the only analyses will be descriptive statistics to summarise eligibility and recruitment to decide whether the trial satisfies the progression criteria.

As the study is not evaluating the surgery per-se, surgical experience is not a criterion for participation (all participants will be under the care of a consultant surgeon). In the context of the GAP study, clustering by surgeon is not relevant to the sample size and can be ignored (on the basis that the intra-class correlation is negligible, personal communication with Prof D Altman for a previous trial).

7.2 Subgroup analyses

Two subgroup analyses are planned: by surgical specialty and by open/minimal access surgery. We will describe the primary and secondary outcomes in the subgroups and test for differences in outcomes between subgroups by including interaction terms in models and/or stratifying models, as appropriate.

7.3 Frequency of analyses

The primary analysis will take place when follow-up is complete for all recruited participants. Safety data will be reported to the DMSC at a frequency to be agreed, together with any additional analyses the committee requests. In these reports, the data will be presented by group but the allocation will remain masked.

7.4 Criteria for the termination of the trial

The trial may be terminated early on the recommendation of the DMSC or the results of another study supersede the necessity for completion of this study.

7.5 Economic evaluation

The economic evaluation will compare the costs and effects of gabapentin for managing post-operative pain and will follow established guidelines as set out by NICE [25]. The main outcome measure for the economic evaluation will be QALYs [26], estimated using the EQ-5D-5L. Resource use data will be collected, length of stay and any post-operative complications by adding questions to the trial CRFs, supplemented with bespoke resource use questions. Unit costs will be derived from nationally published sources and Trust finances and attached to the resource use data.

We will report the cost and quality of life data for each surgical specialty by trial group and the difference between the groups. Then we will calculate the average cost and outcome on a per patient basis to produce incremental cost-effectiveness ratios for the groups, producing an incremental cost per QALY [27].

8. Trial management

8.1 Trial Oversight

8.1.1 Trial Management Group

The trial will be managed by a trial management group (TMG), which will meet face to face or by teleconference approximately every 6 weeks for the duration of the study. The TMG will be chaired by the Lead applicant and will include the CI/clinical lead and representatives from BTC(CTEU). Other members of the research team will be invited to attend as required.

The TMG will be supported by BTC(CTEU), which is a UK Clinical Research Collaboration registered Clinical Trials Units. BTC(CTEU) will prepare all the trial documentation and data collection forms, specify the randomisation scheme, develop and maintain the study database, check data quality as the trial progresses, monitor recruitment and manage the trial on a day to day basis.

8.1.2 Investigator Meetings

Investigator meetings will be held approximately every 6-12 months to review study progress and address any issues that arise. All team members, including all study applicants, local PIs and lead research nurses will be invited to these meetings.

8.2 Day-to-day management

A research nurse in each centre will be responsible for identifying potential trial participants, ensuring clinician (or appropriately trained and qualified non-medical clinician) confirmation of eligibility and written informed participant consent, randomising participants, liaising with the theatre planning manager, collecting trial data and ensuring the trial protocol is adhered to.

8.3 Monitoring of sites

8.3.1 Initiation visit

Before the study commences training session(s) will be organised by the trial coordinating centre BTC(CTEU). These sessions will ensure that all personnel involved fully understand the protocol, CRFs and the practical procedures for the study.

8.3.2 Site monitoring

BTC(CTEU) will carry out central monitoring and audit of compliance of centres/surgical specialties with the principles of Good Clinical Practice (GCP) and data collection procedures. The study database will have extensive in-built validation and the TMG will review the completeness and consistency of the data throughout the trial. BTC(CTEU) will not check CRFs against the data entered or against source data, unless there are good reasons to visit the site to complete a monitoring visit (e.g. the central monitoring highlights a problem). As this is a blinded study any misclassification errors should have minimal impact on the study results.

8.4 Trial Steering Committee and Data Monitoring and Safety Committee

The Trial Steering Committee (TSC) is made up of representatives of GAP TMG, and independent members to be appointed by the funders.

The Data Monitoring and Safety Committee (DMSC) consists of medical statisticians and medical experts in this field. Independent members will be appointed by the funder. The lead statistician for GAP will facilitate these meetings. The Lead applicant and CI/clinical lead will be available as required.

9. Safety reporting

9.1 Definitions

An adverse event (AE) is any undesirable event in a subject receiving treatment according to the protocol, including occurrences which are not necessarily caused by or related to administration of the research procedures.

An adverse reaction (AR) is any undesirable experience that has happened a subject while taking a drug that is suspected to be caused by the drug or drugs.

A serious adverse event (SAE) is any event which result in death, is life threatening, requires hospitalisation or prolongs hospitalisation, results in persistent or significant disability or incapacity.

A suspected serious adverse reaction (SSAR) is any serious adverse event that is suspected to be related to the drug or drugs being taken.

Suspected unexpected serious adverse reaction (SUSAR) is an untoward medical occurrence suspected to be related to the drug or drugs being taken that is not consistent with the applicable product information and is serious.

9.2 Overview

Serious and other adverse events (AEs) will be recorded and reported in accordance with GCP guidelines and BTC(CTEU), Bristol's Serious Adverse Events and Safety Reporting Standard Operating Procedure (SOP-GE-012) (see Figure 2).

Figure 2 Serious adverse event reporting flow chart

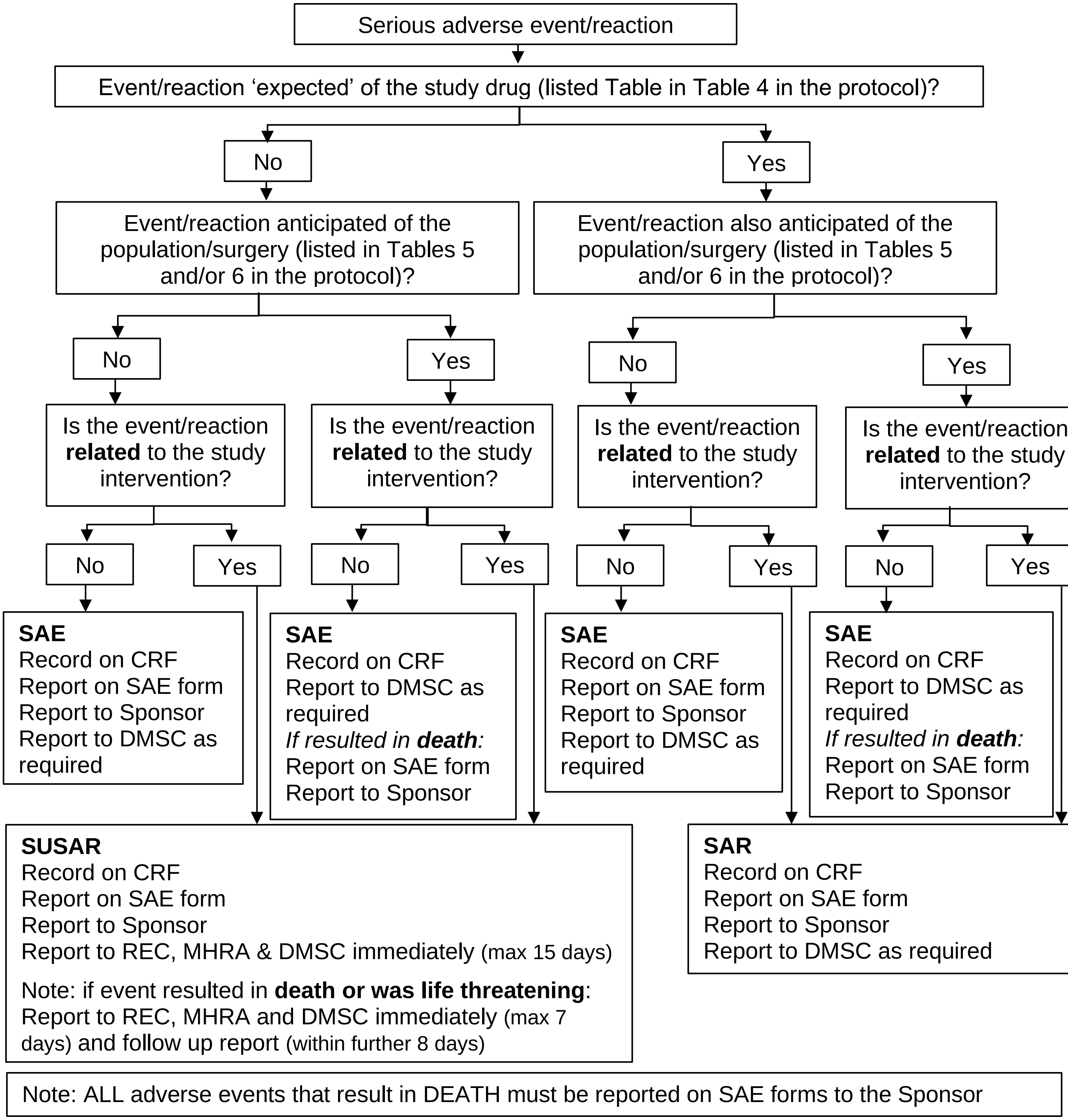

In surgery, post-operative complications are not unexpected and are not infrequent, often causing an extension of the patient's hospital admission. These complications are classified as 'anticipated' (see section 9.4, Table 5) and will not require expedited reporting to the Sponsor, MHRA or REC. It is also anticipated that a significant proportion of the study participants will go on to have adjuvant chemotherapy or radiotherapy after their surgery for cancer. Such

treatments are commonly associated with serious side effects and toxicities. A list of AEs that are considered ‘anticipated’ for patients undergoing chemotherapy and radiotherapy are listed in section 9.4, Table 6 and will not require expedited reporting to the Sponsor, MHRA or REC.

The research team will notify all SAEs to the Sponsor (and MHRA and REC as required), with the exception of the known complications of surgery and /or chemotherapy and radiotherapy (i.e. events listed as ‘anticipated’ events in Tables 5 and 6, Section 9.4).

Expected events are those associated with taking gabapentin, and are listed in Table 4, Section 9.3. Expected events of gabapentin which are also anticipated events of surgery and/or adjuvant chemotherapy and radiotherapy have been highlighted in grey in Table 4. These events will be treated as ‘anticipated’ events and will not require expedited reporting to the Sponsor, MHRA or REC **unless they are classified as being related to the study drug**.

The research team will notify the Sponsor of **any adverse event which results in death**.

All AEs during the participant’s hospital stay and SAEs after hospital discharge will be recorded in detail on a CRF. At the conclusion of the study, all AEs recorded during the study will be subject to statistical analysis, and the analysis and subsequent conclusions will be included in the final study report.

For all events that require expedited reporting an initial report will be completed within 24 hours of becoming aware of the event. The subject will be actively followed up, and the investigator (or delegated person) will provide information missing from the initial report within five working days of the initial report. The first follow-up report will be provided within 5 days of the initial report and subsequent information will be provided each time new information is available until the SAE has resolved, unless a different reporting period is agreed with the Sponsor on a case by case basis, or a decision for no further follow-up has been taken.

Note: Further elective surgery or intervention (e.g. chemotherapy or radiotherapy) during the follow-up period that was planned prior to recruitment to the trial will not be reported as an unexpected SAE.

9.3 Expected adverse events associated with the study medication

This section forms the RSI. The list shown in Table 4 has been adapted from the SmPC for gabapentin 300mg capsules.

The ARs observed during clinical studies conducted in epilepsy (adjunctive and monotherapy) and neuropathic pain have been provided in a single list (see below) by class and frequency (very common (> 1/10); common (>1/100, <1/10); uncommon (>1/1000, <1/100); rare (>1/10,000; <1/1,000); very rare (<1/10,000). Where an AR was seen at different frequencies in clinical studies, it was assigned to the highest frequency reported. Additional reactions reported from the post-marketing experience are included as frequency 'Not known' (cannot be estimated from the available data) in the list below. Within each frequency grouping, undesirable effects are presented in order of decreasing seriousness.

Table 4 Expected adverse events associated with the study medication

Body system	Adverse event	Frequency
Infections and infestations	Viral infection	Very Common
	Pneumonia, respiratory infection, urinary tract infection, infection	Common
	Otitis media	Common
Blood and the lymphatic system disorders	Leucopenia	Common
	Thrombocytopenia	Not known
Immune system disorders	Allergic reactions (e.g. urticaria)	Uncommon
	Anaphylaxis, hypersensitive syndrome (a systemic reaction with a variable presentation that can include fever, rash, hepatitis, lymphadenopathy, eosinophilia and sometimes other signs and symptoms)	Not known
Metabolism and nutrition disorders	Anorexia, increased appetite	Common
	Hyperglycaemia (most often observed in patients with diabetes)	Uncommon
	Hypoglycaemia (most often observed in patients with diabetes)	Rare
	Hyponatraemia	Not known
Psychiatric disorders	Hostility, confusion and emotional lability, depression, anxiety, nervousness, thinking abnormal	Common
	Agitation	Uncommon
	Hallucinations	Not known
Nervous system disorders	Somnolence, dizziness	Very common
	Ataxia	Very common
	Insomnia	Common
	Convulsions, hyperkinesia, dysarthria, amnesia, tremor, headache, sensations such as paresthesia, hypaesthesia, coordination abnormal, nystagmus, increased, decreased, or absent reflexes	Common
	Mental impairment	Uncommon
	Hypokinesia	Uncommon
	Loss of consciousness	Rare
	Other movement disorders (e.g. choreoathetosis, dyskinesia, dystonia),	Not known
Eye disorders	Visual disturbances such as amblyopia, diplopia	Common
Ear and Labyrinth disorders	Vertigo	Common
	Tinnitus	Not known
Cardiac disorders	Palpitations	Uncommon
Vascular disorder	Hypertension, vasodilatation	Common
Respiratory, thoracic and mediastinal disorders	Dyspnoea, cough	Common
	Bronchitis, pharyngitis, rhinitis	Common
	Respiratory depression	Rare
Gastrointestinal disorders	Vomiting, nausea, diarrhoea, abdominal pain,	Common

Body system	Adverse event	Frequency
	dyspepsia, constipation, dry mouth or throat, flatulence	
	Dental abnormalities, gingivitis	Common
	Dysphagia	Uncommon
	Pancreatitis	Not known
Hepatobiliary disorders	Hepatitis, jaundice	Not known
Skin and subcutaneous tissue disorders	Facial oedema, purpura most often described as bruises resulting from physical trauma, rash, pruritus, acne	Common
	Stevens-Johnson syndrome, angioedema, erythema multiforme, alopecia, drug rash with eosinophilia and systemic symptoms	Not known
Musculoskeletal, connective tissue and bone disorders	Arthralgia, myalgia, back pain, twitching	Common
	Rhabdomyolysis, myoclonus	Not known
Renal and urinary disorders	Acute renal failure, incontinence	Not known
Reproductive system and breast disorders	Impotence	Common
	Breast hypertrophy, gynaecomastia, sexual dysfunction (including changes in libido, ejaculation disorders and anorgasmia)	Not known
General disorders and administration site conditions	Fatigue, fever	Very Common
	Peripheral oedema, pain	Common
	Abnormal gait, asthenia, malaise, flu syndrome	Common
	Generalised oedema	Uncommon
	Chest pain	Not known
	Withdrawal reactions (mostly anxiety, insomnia, nausea, pains, sweating). Sudden unexplained deaths have been reported where a causal relationship to treatment with gabapentin has not been established	Not known
Investigations	WBC (white blood cell count) decreased, weight gain	Common
	Elevated liver function tests SGOT (AST), SGPT (ALT) and bilirubin	Uncommon
	Blood creatine phosphokinase increased	Not known
Injury and poisoning	Accidental injury, fracture (non-surgical), abrasion	Common
	Fall	Uncommon

Note: The events highlighted in grey are expected adverse reactions associated with gabapentin but are also known complications of surgery and/or chemotherapy/radiotherapy (also highlighted in grey in Tables 5 & 6).

Under treatment with gabapentin cases of acute pancreatitis were reported. Causality with gabapentin is unclear. In patients on haemodialysis due to end-stage renal failure, myopathy with elevated creatine kinase levels has been reported. Respiratory tract infections, otitis media, convulsions and bronchitis were reported only in clinical studies in children.

9.4 Anticipated events associated with surgery or adjuvant chemotherapy/radiotherapy

The AEs ‘anticipated’ in patients undergoing the operations included in this study are listed in Table 5. A list of AEs that are considered ‘anticipated’ for patients undergoing chemotherapy and radiotherapy, have been identified in Table 6.

9.5 Period for recording serious adverse events

Data on SAEs will be collected for each participant from the point at which they consent until the end of their follow-up period or withdrawal from the study.

Table 5 Anticipated adverse events of surgery

Body system	Anticipated event
Cardiovascular	Treatment of hypertension or need for vasodilators:<br>• Haemodynamic control (use of vasodilators)
	Haemodynamic support:<br>• Use of any inotropes<br>• Use of intra-aortic balloon pump (IABP)<br>• Use of a pulmonary artery catheter
	Arrhythmias / palpitations, including:<br>• Supraventricular tachycardia / atrial fibrillation / atrial flutter<br>• Ventricular tachycardia
	Cardiac pacing
	Myocardial Infarction
	Cardiac arrest, requiring:<br>• Resuscitation involving ventricular defibrillation / direct current (DC) shock<br>• Chest reopening<br>• External/internal cardiac massage
	Congestive heart failure requiring treatment
	Pericarditis requiring treatment
	Bleeding (needing re-operation or not)
	Blood clots
	Haematoma
	Deep vein thrombosis (DVT)
	Pulmonary embolus (PE)
	Peripheral thrombophlebitis
	Pericardial effusion
Blood & lymphatic system disorders	Thrombocytopenia
Pulmonary / respiratory	Respiratory depression
	Pneumonia
	Respiratory infection

Body system	Anticipated event
	Dyspnoea
	Cough
	Intra-thoracic collection/abscess (requiring drainage or not)
	Intubation / re-intubation and ventilation
	Tracheostomy
	Initiation of mask continuous positive airway pressure (CPAP) or non-invasive ventilation
	Acute respiratory distress syndrome (ARDS)
	Pneumothorax
	Acute respiratory failure
	Atelectasis / pulmonary collapse
	Surgical emphysema (requiring intervention)
	Bronchopleural fistula
	Prolonged air leak
	Chylothorax
	Pleural effusion
	Acute aspiration
	Tracheobronchial injury
Renal / Urology	Acute renal failure / Acute Kidney Injury
	Urinary tract infection
	Incontinence
	Urinary retention requiring reinsertion of urinary catheter, delaying discharge, or discharge with urinary catheter
Infective	Sepsis
	Wound infection
	Central intra-venous line infection requiring removal or antibiotics
	Infection (other, with or without antibiotics)
Gastrointestinal (GI)	Pancreatitis
	Vomiting
	Nausea
	Diarrhoea
	Abdominal pain
	Dyspepsia
	Constipation
	Dry mouth or throat
	Flatulence
	Intra-abdominal collection/abscess (requiring drainage or not)
	Dysphagia
	Peptic ulcer / GI bleed / perforation
	Diagnostic laparotomy / laparoscopy
	Leak from any anastomosis, staple line, or localised conduit necrosis
	Conduit necrosis / failure

Body system	Anticipated event
	Intestinal ischaemia
	Ileus (defined as small bowel dysfunction preventing or delaying enteral feeding)
	Intestinal obstruction
	Feeding J-tube complication
	Pyloromyotomy / pyloroplasty complication
	Delayed gastric emptying requiring intervention or delaying discharge or requiring maintenance of NG drainage >7days post-operatively
	Ascites
	Anastomotic stricture (requiring endoscopic intervention)
	New onset diabetes
Hepatobiliary disorders	Hepatitis
	Jaundice
Metabolism and nutrition disorders	Anorexia, increased appetite
	Hyperglycaemia (most often observed in patients with diabetes)
	Hypoglycaemia (most often observed in patients with diabetes)
	Hyponatraemia
Psychiatric disorders	Acute delirium (including hostility, confusion and emotional lability, depression, anxiety, nervousness, thinking abnormal)
	Agitation
	Hallucinations
	Acute psychosis
	Delirium tremens
Neurological / Nervous system disorders	Somnolence, dizziness
	Insomnia
	Mental impairment
	Permanent stroke
	Transient ischaemic attack (TIA)
	Recurrent laryngeal nerve damage
	Other neurological injury
Eye disorders	Visual disturbances such as amblyopia, diplopia
Musculoskeletal	Arthralgia, myalgia, back pain, twitching
General disorders & administration site conditions	Fatigue, fever
	Peripheral oedema
	Pain
	Generalised oedema
Investigations	Weight gain
	Elevated liver function tests SGOT (AST), SGPT (ALT) and bilirubin
Surgical / medical procedure	Re-operation due to any cause
	Bronchoscopy for any cause
Disease specific (oncology)	Disease recurrence / progression; includes local, regional and distant recurrence

Body system	Anticipated event
	New primary and secondary cancers
Injury, poisoning, procedural complication	Wound dehiscence
	Incisional hernia
	Acute diaphragmatic hernia
	Conversion from minimal access surgery to open surgery, for any reason
	Recurrent laryngeal nerve damage
	Genital/renal tract injury
	Chyle leak / chylous ascites
	Surgical complications, including anatomical/surgical damage (e.g. aortic rupture)

Note: The events highlighted in grey are known complications of surgery but are also expected adverse reactions associated with gabapentin (also highlighted in grey in Table 4).

Table 6 Anticipated adverse events for patients undergoing adjuvant chemo- and radiotherapy

Body system	Anticipated event
Blood & lymphatic complications	Thrombocytopenia
	Leucopenia
	Anaemia
	Myelosuppression
Gastrointestinal complications	Nausea
	Vomiting
	Diarrhoea
	Constipation
Infectious complications	Sepsis
	Wound infection
	Respiratory infection
Nervous system complications	Insomnia
	Peripheral sensory neuropathy
	Peripheral motor neuropathy
Muscular complications	Arthralgia
	Myalgia
	Elevated liver function tests SGOT (AST), SGPT (ALT) and bilirubin
	Elevated alkaline phosphatase

Note: The events highlighted in grey are known complications of adjuvant chemo- and radio-therapy but are also expected adverse reactions associated with gabapentin (also highlighted in grey in Table 4).

10. Ethical considerations

10.1 Review by an NHS Research Ethics Committee

The research will be performed subject to a favourable opinion from an NHS REC and Health Research Authority (HRA), including any provisions of Site Specific Assessment (SSA), and local site capacity and capability confirmation. Ethics review of the protocol for the trial and other trial related essential documents (e.g. PIL and consent form) will be carried out by a UK NHS REC. Any subsequent amendments to these documents will be submitted to the REC and HRA for approval prior to implementation.

10.2 Risks and anticipated benefits

Gabapentin is commonly prescribed peri-operatively by individual anaesthetists as part of local acute pain protocols. The main risk to participants will be related to the side effects of gabapentin. Potential risks and adverse events for gabapentin are listed in section 9.3. However, gabapentin is usually well tolerated and side effects are usually short lived and will stop on stopping of the medication.

The main benefit to participants is the potential for quicker recovery after surgery and fewer side effects from opioid medication.

Optimal analgesia is critical for both patients and healthcare systems. The main potential benefit to society is improved patient experience of surgery and post-operative recovery, which can lead to quicker discharge from hospital and therefore improve efficiency and flow through the healthcare system.

10.3 Informing potential study participants of possible benefits and known risks

Information about possible benefits and risks of participation will be described in the PIL.

10.4 Obtaining informed consent from participants

All participants will be required to give written informed consent. This process, including the information about the trial given to patients in advance of recruitment, is described above in section 6.10.

The research nurse/trial coordinator/PI/co-investigator/clinical research fellow will be responsible for the consent process, which will be described in detail in the Trial Manual/working instructions.

10.5 Co-enrolment

Participants will not be permitted to co-enrol in the GAP study if they are receiving active drug therapy as part of the interventional phase of another clinical trial of an investigational medicinal product (CTIMP). Participants will not be permitted to co-enrol in other CTIMP trials during the interventional phase of the GAP trial. Participants will be permitted to take part in other interventional or non-interventional studies (e.g. observational studies) as long as the burden placed on the patient is reasonable and the other trial protocol permits this (to be agreed on a trial by trial basis).

11. Research governance

This study will be conducted in accordance with:

- The Medicine for Human Use (Clinical Trial) Regulations 2004
- GCP guidelines
- Research Governance Framework for Health and Social Care
- European Union Directive 2001/20/EC on clinical trials

11.1 Sponsor approval

Any amendments to the study documents must be approved by the Sponsor, TSC and funder prior to submission to the HRA/REC/MHRA.

11.2 NHS approval

Agreement from the local NHS Trust is required prior to the start of the study at each site.

Any amendments to the study documents approved by the HRA/REC/MHRA will be submitted to the Trust for information or approval as required.

11.3 Investigators' responsibilities

Investigators will be required to ensure that local research approvals have been obtained and that any contractual agreements required have been signed off by all parties before recruiting any participant. Investigators will be required to ensure compliance to the protocol and study manual and with completion of the CRFs. Investigators will be required to allow access to study documentation or source data on request for monitoring visits and audits performed by the Sponsor or BTC(CTEU) or any regulatory authorities.

Investigators will be required to read, acknowledge and inform their study team of any amendments to the study documents approved by the HRA/REC/MHRA that they receive and ensure that the changes are complied with.

11.4 Monitoring by sponsor

The study will be monitored and audited in accordance with UHBW's Monitoring and Oversight of Research Activity SOP, which is consistent with the Research Governance Framework and the Medicines for Human Use (Clinical Trials) Regulations 2004. All study related documents will be made available on request for monitoring and audit by the sponsor (or BTC(CTEU) if they have been delegated to monitor see section 8.3.2), the relevant REC and for inspection by the MHRA or other licensing bodies. Some elements of monitoring will be delegated to BTC(CTEU) and a monitoring plan will be agreed.

11.5 Indemnity

This is an NHS-sponsored research study. For NHS sponsored research if there is negligent harm during the clinical trial when the NHS body owes a duty of care to the person harmed, NHS Indemnity covers NHS staff, medical academic staff with honorary contracts, and those conducting the trial. NHS indemnity does not offer no-fault compensation and is unable to agree

in advance to pay compensation for non-negligent harm. Ex-gratia payments may be considered in the case of a claim.

11.6 Clinical Trial Authorisation

Gabapentin is classed as an investigational medicinal product and a Clinical Trial Authorisation (CTA) from the MHRA will be in place before starting the trial.

12. Data protection and participant confidentiality

12.1 Data protection

Data will be collected and retained in accordance with the UK Data Protection Act 2018.

12.2 Data handling and storage for participant electronic consent (e-consent) data

Electronic consent will be available through a Research Electronic Data Capture (REDCap) e-consent module for participants who choose to use this method of consent. Access to the REDCap e-consent module will be granted to authorised members of the local research team and coordinating centre. Participant email addresses and completed consent forms will be stored in the REDCap e-consent module on a University of Bristol server. A log of all email invitations will be retained in the REDCap system which can only be accessed by BTC(CTEU) staff. No data will be transferred out of the REDCap e-consent module. Data will be archived for 15 years after the end of the trial.

A copy of the final PDF detailing the patient's consent and member of the local research team's confirmation of obtaining consent will be uploaded to the main study database held on the NHS server, where all other participant study data will be held (see section 12.3).

12.3 Data handling, storage and sharing of main study data

12.3.1 Data handling

All participant data (with the exception of e-consent data as outlined above 12.2) will be entered into a purpose-designed server database hosted on the NHS network. Information capable of identifying individuals and the nature of treatment received will be held in the database with passwords restricted to GAP study staff at the participating site and the co-ordinating centre. Information capable of identifying participants will not be made available in any form to those outside the study.

Access to the database will be via a secure password-protected web-interface . Study data transferred electronically to the University of Bristol network for statistical analyses will be pseudonymised and transferred via a secure network. The participants will be identified using their name and unique study identifier on the secure NHS hosted database.

Data will be entered promptly and data validation and cleaning will be carried out throughout the trial. The trial manual will cover database use, data validation and data cleaning. The manual will be available and regularly maintained.

12.3.2 Data storage

All study documentation will be retained in a secure location during the conduct of the study and 15 years after the end of the study, when all patient identifiable paper records will be destroyed by confidential means. Where trial related information is documented in the medical records, these records will be identified by a label bearing the name and duration of the trial in accordance to UHBW NHS Foundation Trust policy. In compliance with the MRC Policy on Data Sharing, relevant 'meta'-data about the trial and the full dataset, but without any participant identifiers other than the unique participant identifier, will be held indefinitely (University server). A secure electronic 'key' with a unique participant identifier, and key personal identifiers (e.g. name, date of birth, address and NHS number) will also be held indefinitely, but in a separate file and in a physically different location (NHS hospital server). These will be retained because of the potential for the raw data to be used subsequently for secondary research.

12.3.3 Data sharing

Data will not be made available for sharing until after publication of the main results of the study. Thereafter, anonymised individual patient data will be made available for secondary research, conditional on assurance from the secondary researcher that the proposed use of the data is compliant with the MRC Policy on Data Sharing regarding scientific quality, ethical requirements and value for money. A minimum requirement with respect to scientific quality will be a publicly available pre-specified protocol describing the purpose, methods and analysis of the secondary research, e.g. a protocol for a Cochrane systematic review. The second file containing patient identifiers would be retained for record linkage or a similar purpose, subject to confirmation that the secondary research protocol has been approved by a UK REC or other similar, approved ethics review body. Patient identifiers would not be passed on to any third party.

13. Dissemination of findings

The findings will be disseminated by usual academic channels, i.e. presentation at international meetings, as well as by peer-reviewed publications (including a full report to the NIHR-HTA programme) and through patient organisations and newsletters to patients, where available.

14. References

1. Dumville JC, Gray TA, Walter CJ, Sharp CA, Page T, Macefield R, Blencowe N, Milne TK, Reeves BC, Blazeby J: **Dressings for the prevention of surgical site infection**. *The Cochrane database of systematic reviews* 2016, **12**:Cd003091.
2. Apfelbaum JL, Chen C, Mehta SS, Gan TJ: **Postoperative pain experience: results from a national survey suggest postoperative pain continues to be undermanaged**. *Anesthesia and analgesia* 2003, **97**(2):534-540, table of contents.
3. Gan TJ, Habib AS, Miller TE, White W, Apfelbaum JL: **Incidence, patient satisfaction, and perceptions of post-surgical pain: results from a US national survey**. *Current medical research and opinion* 2014, **30**(1):149-160.
4. Dolin SJ, Cashman JN, Bland JM: **Effectiveness of acute postoperative pain management: I. Evidence from published data**. *British journal of anaesthesia* 2002, **89**(3):409-423.
5. Katz J, Seltzer Z: **Transition from acute to chronic postsurgical pain: risk factors and protective factors**. *Expert review of neurotherapeutics* 2009, **9**(5):723-744.
6. Chapman CR, Vierck CJ: **The Transition of Acute Postoperative Pain to Chronic Pain: An Integrative Overview of Research on Mechanisms**. *The journal of pain : official journal of the American Pain Society* 2017, **18**(4):359.e351-359.e338.
7. Kinney MA, Hooten WM, Cassivi SD, Allen MS, Passe MA, Hanson AC, Schroeder DR, Mantilla CB: **Chronic postthoracotomy pain and health-related quality of life**. *The Annals of thoracic surgery* 2012, **93**(4):1242-1247.
8. Alam A, Gomes T, Zheng H, Mamdani MM, Juurlink DN, Bell CM: **Long-term analgesic use after low-risk surgery: a retrospective cohort study**. *Archives of internal medicine* 2012, **172**(5):425-430.
9. Clarke H, Soneji N, Ko DT, Yun L, Wijeysundera DN: **Rates and risk factors for prolonged opioid use after major surgery: population based cohort study**. *BMJ (Clinical research ed)* 2014, **348**:g1251.
10. Straube S, Derry S, Moore RA, Wiffen PJ, McQuay HJ: **Single dose oral gabapentin for established acute postoperative pain in adults**. *The Cochrane database of systematic reviews* 2010(5):Cd008183.
11. Alayed N, Alghanaim N, Tan X, Tulandi T: **Preemptive use of gabapentin in abdominal hysterectomy: a systematic review and meta-analysis**. *Obstetrics and gynecology* 2014, **123**(6):1221-1229.
12. Yu L, Ran B, Li M, Shi Z: **Gabapentin and pregabalin in the management of postoperative pain after lumbar spinal surgery: a systematic review and meta-analysis**. *Spine* 2013, **38**(22):1947-1952.
13. Han C, Li XD, Jiang HQ, Ma JX, Ma XL: **The use of gabapentin in the management of postoperative pain after total knee arthroplasty: A PRISMA-compliant meta-analysis of randomized controlled trials**. *Medicine* 2016, **95**(23):e3883.
14. Hwang SH, Park IJ, Cho YJ, Jeong YM, Kang JM: **The efficacy of gabapentin/pregabalin in improving pain after tonsillectomy: A meta-analysis**. *The Laryngoscope* 2016, **126**(2):357-366.
15. Maitra S, Baidya DK, Bhattacharjee S, Som A: **[Perioperative gabapentin and pregabalin in cardiac surgery: a systematic review and meta-analysis]**. *Revista brasileira de anestesiologia* 2017.
16. Sanders JG, Dawes PJ: **Gabapentin for Perioperative Analgesia in Otorhinolaryngology-Head and Neck Surgery: Systematic Review**. *Otolaryngology--head and neck surgery : official journal of American Academy of Otolaryngology-Head and Neck Surgery* 2016, **155**(6):893-903.

17. Zhai L, Song Z, Liu K: **The Effect of Gabapentin on Acute Postoperative Pain in Patients Undergoing Total Knee Arthroplasty: A Meta-Analysis**. *Medicine* 2016, **95**(20):e3673.
18. Felder L, Saccone G, Scuotto S, Monks DT, Carvalho JCA, Zullo F, Berghella V: **Perioperative gabapentin and post cesarean pain control: A systematic review and meta-analysis of randomized controlled trials**. *European Journal of Obstetrics, Gynecology, & Reproductive Biology* 2019, **233**:98-106.
19. Toma O, Persoons B, Pogatzki-Zahn E, Van de Velde M, Joshi GP, collaborators PWG: **PROSPECT guideline for rotator cuff repair surgery: systematic review and procedure-specific postoperative pain management recommendations**. *Anaesthesia* 2019, **74**(10):1320-1331.
20. Verret M, Lauzier F, Zarychanski R, Perron C, Savard X, Pinard AM, Leblanc G, Cossi MJ, Neveu X, Turgeon AF *et al*: **Perioperative Use of Gabapentinoids for the Management of Postoperative Acute Pain: A Systematic Review and Meta-analysis**. *Anesthesiology* 2020, **133**(2):265-279.
21. Nicholson A, Lowe MC, Parker J, Lewis SR, Alderson P, Smith AF: **Systematic review and meta-analysis of enhanced recovery programmes in surgical patients**. *The British journal of surgery* 2014, **101**(3):172-188.
22. Rees JR, Rees M, McNair AG, Odondi L, Metcalfe C, John T, Welsh FK, Blazeby JM: **The Prognostic Value of Patient-Reported Outcome Data in Patients With Colorectal Hepatic Metastases Who Underwent Surgery**. *Clinical colorectal cancer* 2016, **15**(1):74-81.e71.
23. Doleman B, Heinink TP, Read DJ, Faleiro RJ, Lund JN, Williams JP: **A systematic review and meta-regression analysis of prophylactic gabapentin for postoperative pain**. *Anaesthesia* 2015, **70**(10):1186-1204.
24. Chaparro LE, Smith SA, Moore RA, Wiffen PJ, Gilron I: **Pharmacotherapy for the prevention of chronic pain after surgery in adults**. *The Cochrane database of systematic reviews* 2013, **7**:Cd008307.
25. Candotto V, Scapoli L, Gaudio RM, Gianni AB, Bolzoni A, Racco P, Lauritano D, Cura F: **Gabapentin affects the expression of inflammatory mediators on healthy gingival cells**. *International Journal of Immunopathology & Pharmacology*, **33**:2058738419827765.
26. Dolan P: **Modeling valuations for EuroQol health states.** *Medical care* 1997 **35**(11):1095-1108.
27. Gray A CP, Wolstenholme J, Wordsworth S. Applied Methods of Cost-Effectiveness Analysis in Health Care. In: Gray A, Briggs S, editors. Handbooks in Health Economic Evaluation Series. Oxford: Oxford University Press; 2011.

15. Amendments to protocol

Amendment number (i.e. REC and/or MHRA amendment number)	Previous version	Previous date	New version	New date	Brief summary of change	Date of ethical approval (or NA if non-substantial)
0 - Initial application (response to REC/HRA)	1.0	10/10/17	2.0	16/11/17	- Team contact details updated.<br>- Correction of typos (specialty) in sections 5.3, 5.4 and 6.1.<br>- Addition of the word 'address' to section 12.2.2 for consistency with PIL (in response to HRA review).	REC approval on 23/11/2017.
0 - Initial application (response to MHRA)	2.0	16/11/17	3.0	30/11/17	All updates are in response to MHRA requests:<br>- Clarification about updates to the SMPC and their impact on the RSI (protocol) in section 5.6.4<br>- Clarification about general level of care provided to patients in the study (see section 5.6.9).<br>- Clarification in sections 9.2 and 9.4 that all SAEs will be reported to the Sponsor with the exception of SAEs that are known complications of surgery and chemo/radiotherapy (i.e. events listed in the protocol as anticipated events in Tables 5 and 6).	MHRA approval on 06/12/2017.<br><br>Protocol v3.0 submitted as substantial amendment 1 to REC/HRA.
Non-substantial amendment 1.1	3.0	30/11/17	4.0	19/12/17	- The inclusion criterion 'Expected to be able to swallow during the time of the study intervention' has been added for clarity (see section 5.5.1).	HRA approval on 09/01/2018
Substantial amendment 2	4.0	19/12/17	5.0	15/01/18	- Update to sections 5.6 and 5.6.1. Briefly, study medication packs will contain 6 capsules instead of 8 to minimise the chance of participants accidentally receiving more doses of study medication. If surgery is cancelled/postponed a new study medication pack containing 6 capsules will be allocated and any remaining capsules from the first pack will be returned to pharmacy for accountability/destruction.	REC/HRA approval on 29/01/2018.<br><br>MHRA approval on 02/02/2018.

					- Update to section 9.4 (Tables 5 and 6); removal of events of from list of anticipated events of surgery and chemo-/ radiotherapy as they are expected events of gabapentin and clarification about these changes below the tables. Reporting of all expected events of gabapentin was requested by the MHRA previously but removal of these events was missed in error.<br>- Minor clarifications/typos (e.g. 'anticipated' updated to 'expected' in the title of table 4; addition of 'requirement for' to 'haemodynamic support' event in Table 5).	
Substantial amendment 3	5.0	15/01/18	6.0	14/12/18	- Section 5.6.6 and Table 4: Incorporation of respiratory depression from updated SMPC<br>- Sections 6.7 & 8.2: Update stating that appropriately trained/qualified non-medical clinicians are able to confirm/sign study eligibility<br>- Section 6.12: Inclusion of optional patient diary<br>- Section 9.2 (including Figure 2 and Tables 4, 5 & 6): Amended to reflect the updated reporting procedures, namely that expected events of gabapentin which are also anticipated of surgery and/or chemo/ radiotherapy (highlighted in grey in Tables 4-6) will not require expedited reporting to the Sponsor/MHRA/REC unless they are considered related to the study drug; Update to tables 5 & 6 with additional anticipated events of surgery & chemo/radiotherapy<br>- Section 9.2: Update to SAE reporting timeframes in accordance with Sponsor safety reporting guidance	REC approval on 16/01/2019.<br><br>MHRA approval on 15/01/2019.<br><br>HRA approval on 14/03/2019.
Substantial amendment 4	6.0	14/12/18	7.0	18/06/19	- Sections 5.2, 5.3 and 6.9: Update from 2 centres to multiple centres<br>- Section 9.2: Amendment to clarify that all adverse events which result in death will be reported to the Sponsor	REC approval on 30/07/2019.<br><br>MHRA approval on 28/06/2019.

					- Tables 4, 5 and 6: Incorporation of events which had been missed in error and clarification of wording of events and body system classification<br>- Section 12.1: Update to Data Protection Act reference (2018)	HRA approval on 13/08/2019.
Substantial amendment 5	7.0	18/06/19	8.0	03/12/19	- Research team contact details: Updated as two study members (Casali, Abu-Hilal) have left<br>- Section 5.6: Updated to clarify that the first post op dose is due when patients are 'clinically able to swallow' following extubation<br>- Tables 4 and 5: Updated to incorporate dysphagia (in line with the updated SMPC for gabapentin)	REC approval on 17/01/2020.<br><br>MHRA approval on 08/01/2020.<br><br>HRA approval on 17/01/2020.
Substantial amendment 6	8.0	03/12/19	9.0	18/07/21	- Updated Sponsor name from UHBristol to UHBW throughout<br>- Research team contact details updated (new email addresses and title)<br>- Updated trials centre name from CTEU Bristol to BTC(CTEU) throughout<br>- Sections 1, 5.1 & 5.8: Updated sample size<br>- Section 2.2: Update to relevant literature<br>- Section 5.2 & 6.9: Update to length of recruitment period<br>- Section 6.7, 6.10, 10.4 & 12: Update to include remote approach/consent options<br>- Section 7.5 clarification of heading section	MHRA approval on 12/07/2021<br><br>REC and HRA approval on 15/07/2021
