# Supplementary material for: Gabapentin for Pain Management after Major Surgery: A Placebo-controlled, Double-blinded, Randomized Clinical Trial (the GAP Study)
Source: Anesthesiology. 2025 Jul 15;143(4):851–61. doi: 10.1097/ALN.0000000000005655 (PMC12416896; doi:10.1097/ALN.0000000000005655)
Supplement: Supplementary file 2 [file aln-143-851-s002.pdf]

GABAPENTIN IN POST SURGERY PAIN

# Statistical Analysis Plan

| Role | Name | Signature | Date |
|---|---|---|---|
| **Study Statistician** | **Mandy Lui** | | **04/01/2023** |
| **Authorising Statistician** | **Chris Rogers** | | **19/12/2022** |
| **Chief Investigator** | **Ben Gibbison** | | **04/01/2023** |

## Table of contents

**1. Introduction ....3**
1.1 Summary of document ....3
1.1.1 Scope ....3
1.1.2 Planned analyses and dissemination ....3
1.2 Background of study ....3
1.2.1 Peri-operative pain management ....3
1.2.2 Evidence for use of gabapentin in the peri-operative setting ....4
1.2.3 Study rationale ....4
1.2.4 Aims and objectives ....4
**2. Study methods ....5**
2.1 Design ....5
2.2 Randomisation ....5
2.3 Framework ....5
2.4 Sample size ....5
2.5 Blinding ....6
**3. Populations ....6**
3.1 Study populations ....6
3.1.1 Inclusion criteria ....6
3.1.2 Exclusion criteria ....6
3.2 Data sources ....7
3.3 Analysis populations ....7
3.3.1 Intention-to-treat population ....7
3.3.2 Safety population ....7
3.4 Withdrawals ....7
**4. Statistical analyses and report content ....8**
4.1 General content and calculations ....8
4.2 Outcomes ....8
4.2.1 Primary outcome ....8
4.2.2 Secondary outcomes ....8
4.3 Definition and derivation of the outcomes ....9
4.3.1 Primary outcome ....9
4.3.2 Secondary outcomes ....9
4.4 Analysis of the outcomes ....11
4.4.1 Primary outcome ....11
4.4.2 Secondary outcomes ....11
4.5 General content ....13
4.5.1 Participant flow ....13
4.5.2 Baseline data ....14
4.5.3 Trial medication data ....15
4.5.4 Operation and other treatment data ....15
4.5.5 Bang-blinding index ....16
4.5.6 Sensitivity analyses ....16
4.5.7 Subgroup analyses ....16
4.5.8 Further exploratory analyses ....16

4.6 Missing data and outliers ....17
5. References ....17
6. Glossary ....18
7. Revision history ....19

# 1. Introduction

## 1.1 Summary of document

### 1.1.1 Scope

The statistical analysis plan for the GAP study has been written in accordance with Bristol Trials Centre (BTC) standard operating procedures, the CONSORT statement, and International Conference on Harmonisation (ICH) Statistical Principles for Clinical Trials E9. The first version was prepared by Laura Collett, Medical Statistician and Senior Research Associate at BTC, University of Bristol, under the supervision of Professor Chris Rogers, non-clinical lead and senior statistician for the GAP study, and covers all final statistical analyses to be performed, outlined in the study protocol.

### 1.1.2 Planned analyses and dissemination

The end of study statistical report will first include all outcomes based on data collected up to discharge, to be disseminated to the TMG as soon as possible. This report will then be updated to include all remaining outcomes based on data collected up until 4 months post-surgery, and will be disseminated to the TMG when all pre-specified final analyses have been performed[1]. An independent data monitoring and safety committee (DMSC) will review the safety and ethics of the study. Blinded study update reports by intervention will be produced and disseminated on at least an annual basis. The DMSC, in light of the interim reports and of any advice or evidence they wish to request, will if necessary report to the Trial Steering Committee (TSC) if there are any concerns regarding the safety of the intervention or ethics of the study. The DMSC have the authority to recommend that the study stops if deemed necessary based on the observed data.

The final health economic analyses will be documented in a separate health economic analysis plan.

## 1.2 Background of study

### 1.2.1 Peri-operative pain management

In the UK each year, about 4.7 million patients undergo surgery. Many of these patients experience pain after surgery and about 10% experience severe pain. Inadequate pain management increases length of hospital stay, and contributes to the development of chronic or persistent post-surgical pain, which impacts on quality of life. Current multimodal analgesic regimens include paracetamol, non-steroidal anti-inflammatory drugs (NSAIDS) and opioids.

[1] The impact of the COVID-19 pandemic will be assessed in the analyses, including an exploratory analysis on whether the study population/demographics, overall outcomes, or follow-up rates will be affected longitudinally by participants on the study pathway during this time.

Opioids are the key analgesic agents for managing moderate to severe pain. However, they have poor efficacy in movement-associated pain and side-effects including confusion, nausea, vomiting, itching, constipation and respiratory depression. Opioid side effects increase the length of hospital stay, delay overall recovery and impact on quality of life. Reliance on opioids after surgery also increases the risk of opioid dependence and long-term use.

### 1.2.2 Evidence for use of gabapentin in the peri-operative setting

Doctors add gabapentin to multimodal analgesic regimens to try and reduce opioid use while still controlling pain efficiently after surgery, although there is large variation in practice across the UK. Gabapentin is used “off label” in the peri-operative setting, since it is currently only licensed to treat epileptic convulsions and neuropathic pain. Gabapentin is thought to work by binding to calcium channels and reducing calcium influx into nerve cells; this mode of action is responsible for its anti-epileptic, anxiolytic, sedative and analgesic effects.

There are over 130 RCTs that have investigated gabapentin versus placebo in different surgical populations. Most of these RCTs are small (<200 patients, median 80) and highly heterogeneous (statistically and clinically). None has assessed the impact on length of hospital stay or quality of life. These RCTs have been included in 15 systematic reviews that aimed to assess the effectiveness of gabapentin vs. placebo in the peri-operative period. Eight of these reviews included all surgical populations (one of these was restricted to studies administering a single dose of gabapentin) and seven reviews included only single surgical populations (2 head and neck, 2 total knee arthroplasty, 1 spinal lumbar surgery, 1 hysterectomy, 1 cardiac). All reviews included RCTs irrespective of when gabapentin was administered (before surgery, after surgery, or both). All reviews reached the same conclusions – that gabapentin reduced opioid consumption and post-operative pain scores at 24 hours ($P<0.001$). It is difficult to reach conclusions about the optimal dose and duration of treatment because of the heterogeneity of the trials, although the systematic reviews suggest at least 600mg as a starting dose pre-operatively and no less than 300mg/d post-operatively to show a reduction in opioid use. There are no new or ongoing RCTs investigating gabapentin and post-surgery pain that we are aware of.

### 1.2.3 Study rationale

Optimal analgesia is critical for both patients and healthcare systems. Optimal analgesia improves patient experience and allows patients to get out of bed faster. This leads to more rapid discharge and thus improved efficiency and flow to the healthcare system. Reducing opioid use after surgery is a priority for both doctors and patients and is one of the central tenets of enhanced recovery. However, the current evidence base is not robust enough to allow for definitive evidence-based national guidelines on the use of gabapentin in the peri-operative setting. The study research team consulted several patient groups (including patients with different conditions and types of surgery) to get feedback about the importance of the proposed study. There was unanimous support for the study; most patients had experienced negative side effects from opioid use (some severe) and all welcomed any pain medication that would reduce the amount of morphine patients need after surgery. Furthermore, the study team conducted a UK-wide survey of acute pain units and individual consultant anaesthetists, which showed large variation in the prescribing practice of gabapentin-type drugs.

### 1.2.4 Aims and objectives

The trial will assess the effectiveness, cost-effectiveness and safety of gabapentin as an adjunct to standard multimodal analgesia. The trial will aim to assess the following:

A. The difference between groups in the average length of hospital stay following surgery.

B. The difference between groups with respect to a range of secondary outcomes including assessment of efficacy (total opioid use, pain), measures of safety (adverse health events) and health-related quality of life (HRQoL) in the four months following randomisation.
C. The cost effectiveness of gabapentin compared to usual care.

# 2. Study methods

## 2.1 Design

GAP is a phase IV, multi-centre, randomised, double-blind, placebo-controlled, parallel group trial for patients undergoing three types of major surgery: cardiac, thoracic and abdominal.

Participants will be randomised on a 1:1 basis to receive either gabapentin or a placebo, which will be given as: 600 mg gabapentin (or placebo) approximately an hour before surgery; then 600 mg/day (300 mg in the morning and 300 mg in the evening) gabapentin (or placebo) postoperatively for 2 days.

## 2.2 Randomisation

Randomisation will be carried out as close to the planned operation as possible, after eligibility has been confirmed and consent given. Randomisation will be performed by an authorised member of the local research team using a secure internet-based randomisation system ensuring allocation concealment. Participants will be allocated in a 1:1 ratio to either gabapentin or placebo. The allocation will be according to permuted block randomisation, with blocks of varying size. The random allocation to gabapentin or placebo will be stratified by centre and specialty, so that each specialty at each centre will have approximately equal numbers of participants allocated to placebo and gabapentin. Only a unique pack number will be revealed by the randomisation system to maintain blinding.

## 2.3 Framework

The difference between the intervention groups will be compared according to a superiority framework.

## 2.4 Sample size

1180 participants will be randomised to gabapentin or a placebo. This sample size was revised in May 2021 following minimal recruitment during 2020/2021 due to the COVID-19 pandemic; the original sample size was 1500 participants.

Based on the current estimate for length of hospital stay (5 days for cardiac and abdominal surgery, 3 days for thoracic surgery), it is hypothesised that receipt of gabapentin will result in a shorter hospital stay. The sample size has been calculated in order to test the null hypothesis of no difference in the length of hospital stay between the intervention groups.

The target difference in length of hospital stay has been expressed in terms of the change in the proportion of participants discharged at the current median time to discharge. These differences have been chosen to reflect the effect size required in order to change practice.

Based on varying assumptions of: 80% and 90% power; 5% 2-sided type I error rate; 15%, 12.5% and 10% effect sizes; 5% censoring; and constant hazard: sample size calculations have been presented in Table 1. In order to have at least 90% to detect a difference of 12.5% in each specialty, and 80% power to detect a difference of 10% in any specialty, the study would need to

recruit at least 430 participants. Based on these calculations 1500 participants were due to be recruited, across all sites and specialties.

Table 1 Sample size calculations

| Proportion being discharged by current median time to discharge | | Hazard ratio | Power | |
|---|---|---|---|---|
| | | | 80% | 90% |
| Placebo | Gabapentin | | Sample size per specialty | |
| 0.50 | 0.65 | 1.51 | 196 | 262 |
| 0.50 | 0.625 | 1.41 | 280 | **376** |
| 0.50 | 0.60 | 1.32 | 430 | 574 |

As the trial is primarily comparing gabapentin and placebo, in terms of surgical pain and length of hospital stay, surgical experience is not a criterion for participation, and therefore clustering by surgeon is not considered in the sample size calculation (1).

Following minimal recruitment due to the COVID-19 pandemic in 2020/2021 the sample size was reviewed by the Trial Steering Committee and the funder. At this stage in the trial recruitment to the cardiac stratum was complete (500 participants randomised) but the thoracic and abdominal strata were incomplete. It was decided that the power of the study should be reduced to 80%, i.e. a minimum of 280 participants per stratum, which after adjusting for the observed non-compliance rate of 27% in the thoracic and abdominal specialties, was increased to 340 participants, giving a revised total sample of 1180 participants (500 cardiac, 340 thoracic, 340 abdominal).

### 2.5 Blinding

All participants, their clinical care team, their research nurse(s) responsible for follow-up, site pharmacies, and all BTC, with the exception of the unblinded/safety statistician and manufacturing pharmacy, will be unaware of the allocation.

## 3. Populations

### 3.1 Study populations

#### 3.1.1 Inclusion criteria

- Over 18 years of age;
- Undergoing non-emergency surgery:
- ▪ Cardiac (surgery on the heart and great vessels carried out via midline sternotomy);
- ▪ Thoracic surgery (open or minimal access surgery on the lungs and surrounding tissues);
- ▪ Abdominal (open or minimal access surgery within the abdominal cavity);
- Expected to stay in hospital at least until day 2 after surgery (day 0 is day of surgery);
- Expected to be able to swallow during the time of the study intervention.

#### 3.1.2 Exclusion criteria

- Taking anti-epileptic medication(s);

- Allergy to gabapentin;
- Already taking gabapentin or gabapentanoids;
- Rare hereditary problems of galactose intolerance, the Lapp lactase deficiency or glucose galactose malabsorption;
- Planned epidural analgesia;
- Intended use of any gabapentanoids in the peri-operative analgesic protocol other than the study medication (this includes but is not restricted to: pregabalin, enacarbil gabapentin, 4-methylpregabalin and phenibut);
- Known renal impairment (for such patients, estimated glomerular filtration rate (eGFR) <30ml/min/1.73$^2$);
- Weight <50kg;
- Inability to provide written informed consent to participate in the trial;
- Unwilling to participate in follow-up;
- Prisoners;
- Enrolled in another clinical trial and: a) the patient is currently taking an investigational medicinal product as part of the other trial; or b) co-enrolment is not permitted by the other trial; or c) co-enrolment would be burdensome for the patient.

## 3.2 Data sources

Study data will be collected on case report forms (CRFs) and participant completed questionnaires.

## 3.3 Analysis populations

### 3.3.1 Intention-to-treat population

All summaries and analyses of the primary and secondary outcomes (with the exception of the safety outcome) will be conducted on the intention-to-treat (ITT) population. The ITT population will consist of all participants, included according to the intervention group into which they were randomised, regardless of whether they are ineligible, prematurely discontinue treatment or are otherwise protocol deviators. The only exclusions will be participants who have withdrawn consent for their data to be used. Data from these participants will only be used in the CONSORT flowchart and withdrawal summaries.

No per-protocol or CACE analysis will be carried out, as this is a pragmatic study where there is no benefit of assessing the effect of gabapentin in comparison with placebo within ideal conditions, as these conditions are not reflected in practice.

### 3.3.2 Safety population

The safety outcome will be conducted on the safety population, which will consist of all randomised participants, included according to whether they received at least one dose of gabapentin.

## 3.4 Withdrawals

Participants can withdraw/be withdrawn from treatment and/or follow-up at any stage of the study at their own discretion or that of the treating clinician. All participants withdrawn from the study, in addition to those found to be ineligible post-randomisation, will continue to be followed up until discharge, unless they withdraw full consent for further follow-up data to be collected. All data previously collected by the participant will be used in the analysis unless they withdraw consent for previously collected data to be used.

# 4. Statistical analyses and report content

## 4.1 General content and calculations

Statistical analysis is the responsibility of the BTC study statistician. Where interventions are being compared formally using statistical modelling, the placebo group will act as the reference category for interpretations to be in the form: the effect of gabapentin in comparison with placebo. All applicable statistical tests will be 2-sided and will be performed using a 5% significance level, with the exception of tests for interactions that will be performed using a 10% significance level, and confidence interval will be 95% unless otherwise stated. Where data will be summarised descriptively, continuous variables will be summarised using the mean and standard deviation (SD) or median and inter-quartile range (IQR) (depending on the distribution), and categorical data will be summarised as a number and percentage, where all percentages will be calculated using the total number of participants with data available for that variable. Missing or unobtainable data will be detailed in footnotes. Percentages will be rounded to 1 decimal place, and for continuous measures, these will be summarised to one more decimal place than the data is collected. P-values >0.001 will be summarised to 2 significant figures, and those <0.001 will be reported as <0.001. Charts will also be produced to visualise the data, by intervention group, specialty and time where appropriate.

No formal adjustment will be made for multiple testing, but consideration will be taken in interpretation of results to reflect the number of statistical tests performed and the consistency, magnitude and direction of treatment estimates for different outcomes.

## 4.2 Outcomes

### 4.2.1 Primary outcome

- Time-to-discharge

### 4.2.2 Secondary outcomes

Secondary outcome measures include:

- Opioid consumption in the period from surgery until hospital discharge; and from discharge until 4 months
- Acute post-operative pain assessed using the numerical rating scale (NRS) completed at 1 hr, 4 hr, 12 hr post-surgery and then twice daily to discharge
- Adverse health events from randomisation to discharge and serious adverse events up to 4 months
- Health-related quality of life (HRQoL) measured using the EQ-5D 5 level questionnaire and short-form (SF) 12 completed at baseline and at follow-up at approximately 4 weeks and 4 months
- Health economic analysis: Resource use to 4 months (measured during the hospital stay, at 4 weeks and 4 months)
- Pain measured at baseline, at 4 weeks and at 4 months using the brief pain inventory (BPI).

## 4.3 Definition and derivation of the outcomes

### 4.3.1 Primary outcome

Time-to-discharge (TTD) is defined as the time from date and time (datetime) of start of surgery (defined as 'knife to skin') to the date and time of discharge. If a participant is discharged to another hospital / other ward within the hospital after surgery, the final discharge date collected at follow-up will be used.

### 4.3.2 Secondary outcomes

**Opioid consumption**

Participants analgesia is collected at the following timepoints:

- Prior to surgery (pre-operative analgesia: reported on CRFs B2 and D1)
- From surgery until discharge (intra-operative analgesia, post-operative analgesia for each 24 hour period post-operation: reported on CRFs D2-D11 until discharge)
- From discharge until 4 months (analgesia prescribed at discharge, previously reported / ongoing analgesia (as appropriate) at 4 weeks, new analgesia at 4 weeks, previously reported / ongoing analgesia (as appropriate) at 4 months, new analgesia at 4 months: reported on CRFs D16, E2, E3, F2 and F3).

Opioid consumption is defined as the total amount of analgesia each participant receives over the time period of interest (i.e. from surgery to hospital discharge and from hospital discharge to 4 months). Opioids will be expressed as morphine equivalence.

Morphine equivalence will be calculated according to the Gloucestershire Hospitals NHS Trust Opioid Equivalence Chart (2). Opioids not listed on these guidelines will be calculated for morphine equivalence using the Faculty of Pain Medicine of the Royal College of Anaesthetists guidelines (3) where available.

Opioids will include, but is not limited to, the following:

- Codeine
- Dihydrocodeine
- Tramadol
- Fentanyl
- Alfentanil
- Diamorphine
- Morphine (short and long acting)
- Oxycodone (short and long acting)
- Buprenorphine

Remifentanil will be excluded due to insufficient information from the study for calculating opioid consumption.

Other analgesia (e.g. simple non-opioid analgesia and adjuvant medication) will be described separately.

Medications after hospital discharge will be assumed to be taken as prescribed. If an opioid medication is to be taken as needed (PRN) then it will be assumed that it was taken at half the maximum dose per day. If >5% of participants are prescribed PRN opioid medication at or after discharge from hospital the sensitivity of the findings to this assumption will be considered. Where a medication dose is reported on the CRF as a range, the lowest dose in the range will be assumed. (4)

### NRS acute post-operative pain assessment

NRS score will be assessed at rest and on movement at the following timepoints:

- Baseline
- 1 hour post-surgery
- 4 hours post-surgery
- 12 hours post-surgery
- Twice daily until discharge

The scores at these multiple timepoints will be collected on the following CRFs: B4 (baseline), D12 (post-operative timepoints) and D12x if necessary.

### Safety

Safety will be assessed by any reported adverse events (AEs), serious adverse events (SAEs), suspected unexpected serious adverse reactions (SUSARs), and gabapentin-related deaths, determined by routine clinical assessment or reported by the participant. MedDRA System Organ Classes and Preferred Terms will be derived for all events using the most recent version of MedDRA at the time of analysis, and verified by clinical members of the TMG. Events will be reported on the following CRFs: D14, E5, F6 and S1-3 (serious events only) from time to consent until the end of follow-up or withdrawal from the trial.

### HR QoL

Participants will be assessed for their HR QoL using the EQ-5D and SF-12 questionnaires at the following timepoints:

- Baseline
- 4 week follow-up
- 4 month follow-up

The EQ-5D will be scored according to guidelines set out in the EQ-5D user guide (5) where crosswalk index scores will be derived, and the SF-12 will be scored according to Ware et al. (6) for the physical and mental health domains.

### BPI pain assessment

BPI score will be assessed at the following timepoints:

- Baseline
- 4 week follow-up
- 4 month follow-up

The BPI will be scored according to guidelines set out in BPI user guide (7), where: question 1 will form an item on ‘today’s pain’; questions 3-6 on pain severity will form a Pain Severity Index (PSI, the sum of these four items); questions 7-8 form free-text medication and pain relief item and will be relevant to those participants who are, at the time of completion, receiving treatments or medications for pain. As medication data is collected on the study CRFs in a structured manner responses to these questions will not be used. Question 9 sub-questions will form a Pain Interference Index (PII, the sum of these seven items, if the participant has answered at least four out of the seven sub-questions).  If a participant answers ‘no’ to the first question and leaves the remainder of the questionnaire blank, ‘no’ responses will be assumed.

## 4.4 Analysis of the outcomes

For all formal analyses, models will compare the intervention groups, adjusting for site, specialty, and an intervention group by specialty interaction as fixed effects, to estimate the effect of gabapentin compared to placebo for each specialty separately. The overall treatment effect for each outcome will be estimated by fitting a model without the intervention group by specialty interaction term.

### 4.4.1 Primary outcome

**Time-to-discharge**

The primary outcome of time-to-discharge (TTD) will aim to answer objective A (see section 1.2.4). The analysis of whether there is a difference between gabapentin and placebo with respect to time spent in hospital post-surgery will be carried out using a Cox proportional hazards model (8) to compare the intervention groups by calculating the hazard ratios and associated 95% confidence intervals (CIs). Those participants who die before discharge will be censored at the longest recorded length of stay for that specialty, as this is computationally equivalent to competing risk methodology in this setting (9).

The assumptions of the Cox model include non-informative censoring, and proportional hazards. Non-informative censoring should not be an issue due to participants' withdrawal not expected to be related to the probability of being discharged from hospital. The proportional hazards assumption will be assessed for its validity using Schoenfeld residuals and model fit will be assessed using martingale residuals (10) and if the assumption is not met, alternative methods will be explored.

In addition, median time-to-discharge estimates and Kaplan-Meier curves (11) will be produced by intervention group, specialty and overall.

### 4.4.2 Secondary outcomes

The secondary outcomes (with the exception of the health economic outcome: resource use) will aim to answer objective B (see section 1.2.4).

**Opioid consumption**

Opioid consumption will be analysed by calculating the intervention group ratio of total analgesia use over the time period of interest. Ratios between the intervention groups, with 95% CIs calculated using bootstrap estimation with 10,000 replications to estimate the standard error of the mean ratio, will be calculated for each specialty separately and overall. Analgesia will be split into the two time periods of interest and treatment ratios will be compared separately. Forest plots will be used to visually compare the treatment effect for each specialty separately and overall.

All analgesia use per day will also be summarised descriptively by intervention group, specialty and overall.

**NRS acute post-operative pain assessment**

NRS acute post-operative pain over time, at rest and on movement, will be analysed using mixed linear regression models where participants will be classed as random effects, with intervention group, site, specialty and timepoint as fixed effects, with fixed effect interactions between intervention group, specialty and timepoint, where appropriate, to assess the specialty-specific treatment effects and to see if these treatment effects vary over time.

Time as a random intercept will be considered and nested models will be compared using the likelihood ratio test. Model assumptions will be tested using standard methods, including residual

plots etc. If these assumptions do not appear to be valid then alternative methods will be explored, in particular if NRS does not appear to be normally distributed then models with more appropriate outcome distributions will be considered.

NRS pain scores at rest and on movement will also be summarised descriptively by intervention group, specialty, timepoint and overall.

### Safety

The proportion of participants reporting an SAE will be analysed using a logistic regression model to assess the effect of gabapentin in comparison with placebo, adjusted for site, specialty, and an interaction between intervention group and specialty.

The number of adverse events (AEs), serious adverse events (SAEs), serious adverse reactions (SARs), suspected unexpected serious adverse reactions (SUSARs), and deaths, reported and occurring from randomisation until hospital discharge will also be summarised descriptively by intervention received, specialty and overall.

Summaries of all events will also include: expectedness (whether anticipated of surgery, expected of gabapentin, or both); and associated MedDRA system organ class and preferred term, and for serious events only: relatedness; duration (if resolved or died); classification (seriousness); outcome; maximum intensity; and unblinding information.

The number of deaths and causes of death for all participants will be summarised descriptively by intervention received, specialty and overall.

### HR QoL

EQ-5D-5L index score will be analysed longitudinally using a mixed linear regression model. Participants will be classed as random effects, and the intervention groups will be compared adjusting for site, specialty and timepoint as fixed effects, incorporating interactions between intervention group, specialty and timepoint to assess the specialty-specific treatment effects and to compare intervention groups.

SF-12, for both physical and mental, will also be analysed in the same way, depending on the outcome distribution and model fit.

For all HR QoL models, nested models will be compared using the likelihood ratio test and model assumptions will be tested using standard methods, including residual plots etc. If these assumptions do not appear to be valid then alternative methods will be explored.

EQ-5D-5L and SF-12 ordinal scores for all domains of the questionnaires will also be summarised descriptively at each timepoint by intervention group, specialty and overall.

Visual analogue score (VAS) score will also be summarised descriptively at each timepoint by intervention group, specialty and overall.

### BPI pain assessment

BPI pain assessment using the PSI and PII (defined in section 4.3.2) will both be analysed longitudinally using mixed linear regression models where participants will be classed as random effects, and the intervention groups will be compared adjusting for site, specialty and timepoint as fixed effects, incorporating interactions between intervention group, specialty and timepoint to assess the specialty-specific treatment effects and to compare intervention groups in terms of pain measured by the BPI on these two domains. Nested models will be compared using the likelihood ratio test and model assumptions will be tested using standard methods, including residual plots etc. If these assumptions do not appear to be valid then alternative methods will be

explored, in particular as with NRS, if either PSI or PII do not appear to be normally distributed then models with more appropriate outcome distributions will be considered.

Each remaining item of the BPI questionnaire will also be summarised descriptively at each timepoint by intervention group, specialty and overall.

## 4.5 General content

### 4.5.1 Participant flow

The CONSORT (12) flow diagram will be used to summarise the course of participants through screening until follow-up throughout the course of the study[2], this will include the number of:

- Patients undergoing cardiac, thoracic and abdominal surgery
- Patients fulfilling the inclusion criteria
- Eligible patients
- Patients given/sent a patient information leaflet (PIL) and patients approached (if necessary)
- Patients giving consent to participate
- Participants randomised
- Participants allocated to each intervention group
- Participants who receive at least one dose of study drug
- Participants who receive all study doses as per-protocol
- Participants who respond at 4 week follow-up
- Participants who respond at 4 month follow-up
- Participants who withdraw and timing of withdrawal

Protocol deviations and confirmed serious breaches of good clinical practice (GCP) will be summarised descriptively by intervention group, specialty and overall. Protocol deviations are defined as the following:

- Participant does not meet the study eligibility criteria but is randomised into the study (regardless of whether or not any study treatment was received)
- Participant does not meet the study eligibility criteria, but receives at least one dose of study treatment
- Participants who receive too many doses of treatment (including use of stock gabapentin)
- Participant who does not receive all pre-op and post-op trial treatment or receives any dose of trial treatment outside of specified time windows:
- ▪ Pre-op dose: between 12 hours pre-surgery and prior to induction of anaesthesia
- ▪ Post-op dose 1: for intubated participants, between being clinically able to swallow following extubation and 48 hours post-induction of anaesthesia, and for non-intubated participants, between 6 hours after pre-op dose and 48 hours post-surgery end
- ▪ Post-op dose 2: between 6 and 18 hours after post-op dose 1
- ▪ Post-op dose 3: between 18 and 30 hours after post-op dose 1
- ▪ Post-op dose 4: between 30 and 42 hours after post-op dose 1

The reasons for doses not given as per protocol will be presented. The number of withdrawals of consent to the study will be presented, along with reasons for withdrawal. Any information

[2] As mentioned in section 1.1.2, the impact of the COVID-19 pandemic will be assessed including whether the study population/demographics, overall outcomes, or follow-up rates will be affected longitudinally by participants on the study pathway during this time.

referring to participant pathway taken from notes to file reported for each participant will not be presented, but taken into account where appropriate.

### 4.5.2 Baseline data

Baseline characteristics will be summarised descriptively and will include:

- Age
- Sex
- Body mass index (BMI) (derived from weight (kg) and height (cm))
- American Society of Anesthesiologists (ASA) score (I, II, III, IV, V)
- Ethnicity
- History of the following:
    - Diabetes
    - Smoking
    - Congestive heart failure
    - Peripheral vascular disease
    - Cerebrovascular disease
    - Dementia
    - Hypertension requiring treatment
    - Myocardial infarction
    - Chronic pain syndrome
    - Neurological dysfunction
    - Chronic pulmonary disease
    - Rheumatological disease
    - Liver disease
    - Hemiplegia or paraplegia
    - AIDS/HIV infection
    - Renal disease (including eGFR values)
    - Cancer/malignancy (including treatment for such)

Baseline medication history (prior to hospital admission), including dose unit and frequency of drugs received, will be summarised descriptively by intervention group, specialty and overall and will include the proportion of participants receiving:

- Simple analgesia
    - Paracetamol
    - Aspirin
    - Ibuprofen
- Opioids
    - Codeine
    - Tramadol
    - Fentanyl
    - Morphine (short-acting)
    - Morphine (prolonged release)
    - Oxycodone (short-acting)
    - Oxycodone (prolonged release)
    - Buprenorphine
- Adjuvants
    - Pregabalin
    - Gabapentin
- Other analgesia
- Anti-depressants
    - Amitriptyline

    - Nortriptyline
    - Selective serotonin reuptake inhibitors (SSRI)
    - Benzodiazepines
    - Monoamine oxidase inhibitors (MAOI)
    - Duloxetine
    - Flupentixol
    - Mirtazapine
    - Reboxetine
    - Venlafaxine
- Other anti-depressants

### 4.5.3 Trial medication data

Number of capsules received by each participant, at each timepoint (with reference to the ideal administration time window) and in total will be summarised descriptively, along with details of, and reasons for, any dose reductions, omissions, or errors, by intervention group, specialty and overall.

### 4.5.4 Operation and other treatment data

Operation and other treatment data, including operation details, analgesia and anaesthetic received, will be summarised descriptively by intervention group, specialty and overall and will include:

- Operation received (defined by OPCS code and associated text)
- Time from randomisation to operation start time

Analgesia use per day will be summarised descriptively as is described in section 4.3.2 (Opioid consumption), with summaries to include all analgesia listed, in addition to the following adjuvants:

- Magnesium
- Clonidine
- Ketamine
- Lignocaine
- Pregabalin
- Gabapentin (excluding study medication)

Local anaesthetic administered per day from the day of operation until discharge, including route of administration, specific drug, volume, concentration, and start time and end time (where relevant), will be summarised descriptively by intervention group, specialty and overall and will include:

- Spinal
- Paravertebral
- Epidural
- Wound infiltration
- Nerve / fascial block
- Intercostal block
- Other local anaesthetic

Analgesia prescribed at discharge, including dose, units and frequency, will be summarised descriptively by intervention group, specialty and overall and will include the analgesic medications specified in section 4.3.2 and adjuvants listed above.

Participants who undergo mechanical ventilation will be summarised, and participants who move wards will be summarised, including level of care moved to.

Any analgesia or adjuvant treatment received during follow-up, including dose, units and frequency, will be summarised descriptively by intervention group, specialty and overall.

### 4.5.5 Bang-blinding index

Patient and clinical responses to the bang blinding questions at discharge, and for the patient again at 4 month follow-up, will be summarised descriptively by intervention group, specialty and overall in terms of percentage of correct guesses.

### 4.5.6 Sensitivity analyses

The primary outcome analysis will be repeated excluding

a) participants who were randomised but did not meet the eligibility criteria
b) participants recruited at the Liverpool site where a potential serious breach of GCP was identified.

The results of these sensitivity analyses will be sent to the Sponsor upon completion together with the results of the primary analysis.

### 4.5.7 Subgroup analyses

Exploratory subgroup analyses will be carried out to explore the primary outcome in different groups of participants. The analysis will be carried out using the ITT population. The following subgroups will be assessed:

- Sex (Male, Female)
- Type of surgery (Open, minimal access, defined by OPCS code and associated text)

Subgroup analyses may, by chance, generate false negative or positive results. Those carried out will be interpreted with caution and treated as hypothesis-generating.

### 4.5.8 Further exploratory analyses

**Magnesium use**

Due to suggestions of magnesium use in practice as a form of pain relief in this patient population, the use of magnesium will be analysed in terms of patients' NRS acute post-operative pain assessment in order to determine whether there is an interaction between gabapentin and magnesium in patients' perception of pain.

The statistical analysis methods for this exploratory analysis of magnesium will be identical to that used in the main trial outcomes, except that total magnesium use in mmol (any magnesium use reported in g will be converted to mmol) from baseline to discharge will be included as a variable in the model and total magnesium use will be included as a time-varying covariable in the model. The interaction between intervention group and magnesium use will also be included in the models and these models will be compared using the likelihood ratio test for both outcomes. Similar model testing will be used for both models, as stated in the main outcome analyses.

Further exploratory analyses may be carried out should they be deemed necessary; this will be at the discretion of the TMG. These will be added to the analysis plan as an amendment (see section 7) along with justification, where appropriate.

**Impact of COVID-19**

The impact of the COVID-19 pandemic on the study population and follow-up rates will be described. The impact on the primary and secondary outcomes will be explored by adding pre-COVID-19 as a fixed effect to study models (cf. sub-group analyses). Participants randomised

before 23 March 2020 (the first day of restrictions to prevent COVID-19 in the UK) will be considered to be in the pre-COVID-19 era.

## 4.6 Missing data and outliers

A thorough data cleaning process will be carried out and attempts will be made to obtain any missing data by chasing until it is either received, confirmed as not available, or the trial is at the analysis stage. Were data is unobtainable, all summaries will indicate how many missing results there are, by intervention group, where any imbalances can be explored. Where data points are identified as possible outliers both statistically and clinically, and are considerable in number, sensitivity analyses may be considered for all formal outcomes.

### Primary outcome

Dates and times recorded for start of surgery and time of discharge are highly unlikely to be missing or incomplete. In the rare case that any dates and times are missing or incomplete, the missing information will be accessed from surgical and hospital database sources. As time-to-discharge is being measured from start of surgery, participants who do not undergo surgery, will be excluded from the analysis, but given participants are randomised as close to the start of surgery as possible, this eventuality is expected to be rare.

### Opioid consumption

As opioid consumption will be assessed by taking the sum of all doses over time, then missing data may be underestimated. If the proportion of participants who have missing data for opioid consumption in more than one timepoint is >5% then sensitivity analyses including multiple imputation methods will be considered. Multiple imputation models will include participant demographics, treatment allocation, stratification variables, and variables predictive of missing data (e.g. medication at earlier time points and pain score and QoL at earlier time points).

The sensitivity of the findings to the assumptions around PRN dosing and where ranges are given will also be explored by assuming a) participants in the placebo group took maximum doses/maximum range and participants in the placebo group took no PRN medication/minimum range and b) vice versa.

### NRS acute post-operative pain assessment, HR QoL and BPI pain assessment

As the above outcomes are measured on multiple timepoints, the impact of any missing values will be reduced due to the use of longitudinal mixed effects methodology, therefore allowing participants with at least one non-missing value to be included in the analysis. If the proportion of participants that do not have at least one observation is >5% then again a sensitivity analyses including multiple imputation methods will be considered.

# 5. References

1. Altman D. Personal communication during a previous trial, on the basis of the intra-class correlation being negligible for trials where surgery is not the intervention being evaluated.
2. Medicines Information, Cheltenham General Hospital. Gloucestershire Hospitals NHS Trust Opioid Equivalence Chart. 2011 [Available from: https://www.gloshospitals.nhs.uk/gps/treatment-guidelines/opioid-equivalence-chart/.
3. Faculty of Pain Medicine of the Royal College of Anaesthetists: Dose equivalents and changing opioids.
4. Bicket MC, Long JJ, Pronovost PJ, Alexander GC, Wu CL. Prescription Opioid Analgesics Commonly Unused After Surgery: A Systematic Review. JAMA Surg. 2017;152(11):1066-71.

5. EQ-5D-5L User Guide [Available from: https://euroqol.org/wp-content/uploads/2016/09/EQ-5D-5L_UserGuide_2015.pdf.
6. Ware JAK, M & D. Keller, S. SF-12: How to Score the SF-12 Physical and Mental Health Summary Scales. 1998.
7. BPI User Guide [Available from: https://www.mdanderson.org/documents/Departments-and-Divisions/Symptom-Research/BPI_UserGuide.pdf.
8. Cox D. Regression models and life= tables [with discussion] JR Stat Soc. 1972; 34: 187–220. Series B. 1972.
9. Brock GN, Barnes C, Ramirez JA, Myers J. How to handle mortality when investigating length of hospital stay and time to clinical stability. BMC Medical Research Methodology. 2011;11(1):144.
10. Lin DY, Wei LJ, Ying Z. Checking the Cox Model with Cumulative Sums of Martingale-Based Residuals. Biometrika. 1993;80(3):557-72.
11. Kaplan EL, Meier P. Nonparametric Estimation from Incomplete Observations. Journal of the American Statistical Association. 1958;53(282):457-81.
12. Schulz KF, Altman DG, Moher D, Group C. Consort 2010 statement: Updated guidelines for reporting parallel group randomized trials. Annals of Internal Medicine. 2010;152(11):726-32.

# 6. Glossary

| | |
|---|---|
| AE | Adverse Event |
| AR | Adverse Reaction |
| ASA | American Society of Anesthesiologists |
| BPI | Brief Pain Inventory |
| CI | Chief Investigator |
| CRF | Case Report Form |
| CTEU | Clinical Trials and Evaluation Unit |
| CTIMP | Clinical Trial of an Investigational Medicinal Product |
| DMSC | Data Monitoring and Safety Committee |
| eGFR | Estimated Glomerular Filtration Rate: Derived from gender, age, ethnicity and serum creatinine |
| EQ-5D-5L | EuroQol 5 Dimension 5 Level questionnaire |
| HRQoL | Health-Related Quality of Life |
| GCP | Good Clinical Practice |
| ITT | Intention-To-Treat |
| NRS | Numerical Rating Score |
| NSAIDS | Non-Steroidal Anti-Inflammatory Drugs |
| PI | Principal Investigator |
| PIL | Patient Information Leaflet |
| RCT | Randomised Controlled Trial |

| | |
|---|---|
| SAE | Serious Adverse Event |
| SF-12 | Short-Form-12 |
| SOP | Standard Operating Procedure |
| SUSAR | Suspected Unexpected Serious Adverse Reaction |
| TMG | Trial Management Group |
| TSC | Trial Steering Committee |

# 7. Revision history

Version 1 of the SAP should be signed off by relevant personnel before any data analysis is carried out. If changes need to be made to v1.0 before this time, possibly due to emerging methodologies, these changes should be documented in Table 2 below, with new version number, date and a summary of the changes with justification(s). If any changes to the methodologies are required after data analysis has begun, these should be documented in the final analysis report in a chronological manner, documenting all decisions made and their justification(s).

**Table 2 SAP revision history**

| Version number | Revision date | Justification for revision |
|---|---|---|
| 3.0 | 19/12/2022 | Section 4.3.1:<br>Clarified derivation of primary outcome.<br>Section 4.3.2: Source of opioid equivalence chart updated to include all the listed opioids. Remifentanil excluded from list due to insufficient information for calculating opioid consumption<br>Section 4.5.1: Updated logic for identifying study drug administered out of window. Added the reasons of dose not given as per protocol will be presented.<br>Addition of a further sensitivity analysis excluding participants recruited in Liverpool, due to serious breach of GCP at this site.<br>Addition of exploratory analyses investigating the impact of COVID-19 on the study participants and outcomes.<br>Clarified that opioid consumption will be compared separately for the period from surgery to discharge and from discharge to 4 months and that non-opioid analgesia will be described but not compared between groups.<br>Clarified how medications given as a range or as PRN will be handled. Expanded sensitivity analyses to include alternative assumptions re handling of PRN medication. Provided additional information on multiple imputation strategy. |
| 2.0 | 21/07/2021 | Sample size updated – due to minimal recruitment during the 2020/2021 COVID-19 pandemic, the target sample size was reduced from 1500 to 1180. |

| | | |
|---|---|---|
| | | Addition of protocol deviation defined as participant ineligible but randomised into the study (participant may or may not have received any study treatment).<br>Addition of section on sensitivity analyses to include a sensitivity analysis of the primary outcome excluding participants who were randomised but did not meet the eligibility criteria. Added following identification of a number of instances where ineligible patients had been recruited. |
