# Supplementary material for: Gabapentin for Pain Management after Major Surgery: A Placebo-controlled, Double-blinded, Randomized Clinical Trial (the GAP Study)
Source: Anesthesiology. 2025 Jul 15;143(4):851–61. doi: 10.1097/ALN.0000000000005655 (PMC12416896; doi:10.1097/ALN.0000000000005655)
Supplement: Supplementary file 3 [file aln-143-851-s003.pdf]

The GAP Study: Supplementary Material

Collaborators

In addition to the authors, the trial could not have been completed successfully without the support of the following people.

Investigation (research sites)

University Hospitals Bristol and Weston NHS Trust: Louise Flintoff, Rebecca Houlihan, Laura Beacham, Joanna Nicklin, Sofia Athanatou, Karen Bobruk, Katy Tucker, Catherine O'Donovan, Heather Hudson, Danielle Davis, Amelia Lowe, Vivienne Lee, Toni Farmery, Mairead Osborn, Victoria Taylor, Rosina Jarvis, Chiaki Shioi, Brenda Chivima, Maryam Hamdollah-Zadeh, Elysia Gower, Mary Trevelyan, Caroline Horrobin, Phoebe Syme, Mat Molyneux, Alex Middleditch, Charlotte Steeds, Ben Howes, Neil Machatuta, Sam Howell, Shelley Barnes, Kim Wright, Rachel Wyatt, Melanie Ridout, Emma Hopkins, Katrina Spielman, Linta Joseph, Penny Lambert, Siby Varghese, Jessy George, Fran Gill, Sue Plummer, Amy Pagett, Mae Hazel

University Hospital Southampton NHS Foundation Trust: Clare Bolger, Rachel Brown, Charlotte Allan, Kim Golder, Maggie Johns, Fadoua Aarab-Hamidan, Karen Salmon, Rachel Burnish, Anna Walton, Paul Fernandes, Matthew Garrod, Oliver Harrison, Paul Diprose, Pauline Bartlett, Kirstin Wilkinson, Charlotte Merry, Ravi Gill, Omar Al-Azzawi, Oliver Jones, Stephen Sandys, Adnan Raza, Jonathan Huber, Andrew Cumpstey, Efe Evbuomwan, Eva Stina Eriksson, Maria Filippaki, Andy Curry, Jonathan Bliss, Hannah Wardall, Norma Diaper, Alexander Jackson, Lewis Matthews, Mirela Petrova, Kirsty Prager, Michelle Male, Janet Andrews, Holly burton, Laura Broadbent, Sitara Bali, David Harvie, Belinda Roberts, Sue Jackson, Alice Baker, Sophia Beeby, Michael Carter

Somerset NHS Foundation Trust: Richard Birwell, Corinne Pawley, Patricia Doble, Jo Hutter, Benjamin Plumb, Pat Walker, Tessa Dean, Mark Abou-Samra, Jo Morrison, Ilinca Dragusin, Moira Tait, Jasmine Youens, Corinne Pawley, Abdelrahman Alhmooze, Lynn Leat, Richard Innes, Luke Rutter, Tim Richards, Chetan Pataki

Blackpool Teaching Hospitals NHS Foundation Trust: Stacey Donaldson, Andrew Potter, Joanna Brown, Vasanthi Vasudevan, Deepa Sebastian, James Glen, Palanikumar Saravanan, Julie Frudd, Janette Brown, Victoria Godfrey, Karen Pollard, Erik Cartain Farish, Sam Remnant, Sharn Akehurst, Stephanie Plank, Zena Bradshaw, Emma Hatton

Royal United Hospital Bath NHS Foundation Trust: Sarah Hierons, Lidia Ramos, Lucy Howie, Sarah Mitchard, Carrie Demetriou, Emira Kursumovic, Peter Steed, Andrew Shrimpton, Alfredo Serrano, Fiona Kelly, Gabrielle Evans

Liverpool University Hospitals NHS Foundation Trust: Francesca Westwell, Francesco Ferraro, Joanne Doolan, Julie Haydock, Graham Sweeney, Laura Cureton, Hannah Murphy, Kera Hainey, Hema Thomas, Richard Ramsaran, Sarah Skinner, Karen Kavanagh, Rebecca Tangney, Ann Marron, Heather Michael, Jane Parker, Shirley Pringle

Basildon and Thurrock University Hospitals NHS Foundation Trust: Anil Uston, Anne Nicholson, Ishan Pankhania, Felix Lo, James Bartlett-Day, Joanne Riches, Mark Vertue, Miranda Forsey, Modupe Ibayemei, Premalatha Saravanan, Wayne Chicken

Project administration

Barbara Warnes, Rachel Brophy, Surinder Kaur, Manuela Antognozzi, Jade Salter-Hewitt

Software

D Hutton, S Bellani

Trial Steering Committee

Professor Catherine Hewitt (Chair), Deputy Director of York Trials Unit and Senior Statistician, University of York

Dr Gudrun Kunst, Consultant Cardiac Anaesthetist, King's College Hospital

Dr Mark Rockett, Consultant in Anaesthesia and Pain Medicine, Plymouth Hospital

Professor Dyfrig Hughes, Professor of Pharmaco-economics & Co-director of the Centre for Health Economics & Medicines, Bangor University

Mr Alan Osborne, Independent Consultant Upper Gastrointestinal and Bariatric Surgery, North Bristol Trust (to October 2020)

Mr Jim Tiernan, Consultant Colorectal Surgeon, St James's University Hospital (from November 2021)

Ms Hannah Elizabeth Drummond, Patient Representative

Mrs Annie Berry, Patient Representative

Data Monitoring and Safety Committee

Professor Linda Sharples (Chair) Professor of Statistics, London School of Hygiene & Tropical Medicine, London

Mr Raaj Praseedom, Consultant Hepato-Pancreato-Biliary & Transplant Surgeon, Addenbrooke's Hospital, Cambridge

Dr Nick Kennedy, Consultant Anaesthetist and Intensivist, Taunton and Somerset NHS Trust, Taunton (to July 2019)

Dr Ben Shelley, Consultant in Cardiothoracic Anaesthesia and Intensive Care, Golden Jubilee National Hospital, Glasgow (from March 2020)

Statistical methods – additional information

The data are reported in line with the Consolidated Standards of Reporting Trials (CONSORT) reporting guidelines for superiority trials. All analyses were directed by a pre-specified statistical analysis plan, which was finalised before data lock. Participants were grouped according to the randomised allocation (intention to treat), unless indicated otherwise.

Derivation of outcomes

Health-related quality of life (HRQoL) questionnaires were scored according to the developer's scoring instructions, summary scales derived from the questionnaires are reported. Adverse events were coded using the Medical Dictionary for Regulatory Activities (MedDRA) and are reported by system organ class and preferred term. Opioid consumption was derived by converting the amount (e.g. mg) of each opioid taken to an intravenous (IV) morphine equivalent and summing these give a total IV morphine equivalent dose. If the dose and/or the frequency were given as a range, the lowest dose and highest frequency was assumed. For example, for tramadol given orally (per os, PO) 50-100mg is equivalent to 10mg of oral morphine, giving a ratio of oral morphine:tramadol in the range 0.2 (10/50) to 0.1 (10/100). In calculations the smaller ratio (0.1) was used. 10mg of oral morphine was taken to be equivalent to 3.33mg of intravenous (IV) morphine. So, if Ymg of tramadol was prescribed, it was taken to be equivalent to ($Y \times 0.1 \times 0.333$) mg of IV morphine. If the frequency was given as 'when required' (pro re nata, PRN) half the maximum daily dose was assumed. Morphine equivalents were taken from https://www.gloshospitals.nhs.uk/gps/treatment-guidelines/opioid-equivalence-chart/.

Summary statistics and analysis population

Data are described using summary statistics; mean and standard deviation for continuous variables (or median and interquartile range if distributions were skewed) and number and percentage for categorical variables. The analysis population consisted of all randomised participants, excluding those who withdrew and were unwilling for data already collected to be used. Data from any participant who withdrew and was unwilling for their data to be used were included in the study flowchart but not in any subsequent data tables or figures. Participants who did not undergo surgery were excluded from analyses of the primary outcome.

Modelling strategy

Specialty, treatment group and the specialty by treatment group interaction were included in models as fixed effects. If the interaction term was statistically significant at the 10% level or less,

treatment effects for the whole cohort are omitted, otherwise treatment estimates for the trial cohort as a whole are presented alongside the specialty-specific estimates.

For longitudinal outcomes if the interactions with time were statistically significant at the 10% level results are presented for each time period separately otherwise overall estimates are given. Alternative covariance structures were considered and structure giving the lowest Akaike information criterion was chosen. Model fit was assessed graphically (e.g. plots of predicted values versus residuals and normal probability plots). If the model was a poor fit, transformations (e.g. log transformation for data following a log normal distribution) were considered. Missing baseline values were imputed using the specialty-specific median value.

Primary outcome – time from surgery to hospital discharge (in hours)

Withdrawals on the day of surgery, were censored at half a day. Any time spent in another hospital following discharge from the surgical unit but before discharge home was included; if the date of discharge from this other hospital was not known the time to discharge was censored at discharge from the surgical unit) The Efron method was used to account for tied times.

Secondary outcomes - Opioid consumption

The log-linear mixed-effects model of opioid consumption in the period to hospital discharge included site and participant fitted as random effects, and indicators for each post-operative day (up to day 10) and daily opioid use at baseline as fixed effects. The model of opioid consumption during follow-up included clustering by site and daily opioid use at baseline (pre-randomisation) fitted as a fixed effect.

NRS scores at rest and on movement

A two-dimensional fractional polynomial was used for NRS at rest and a three-dimensional fractional polynomial was used for NRS on movement. Site, participant and time (at the participant level) were included as random effects. An unstructured covariance structure was used.

One or more SAE adverse event

These models included clustering by site.

Total dose of each non-opioid analgesic

Mean ratios were derived for each analgesic group and 95% confidence intervals were estimated using bootstrapping (10,000 replications).

Missing data

Missing data are described in footnotes to all tables. Rules for imputing missing data outlined in the statistical analysis plan were dependent on the level of missing data. HRQoL scores (EQ-5D-5L, SF-12, BPI) met the threshold for multiple imputation. For other outcomes participants with missing data were excluded. For HRQoL analyses, each subscale was imputed separately. Multivariable imputation by chained equations using predictive mean matching (of 10 nearest neighbours) was used to generate multiple complete datasets and results were combined using Rubin's rules. Factors included in the matching were baseline HRQoL score, treatment allocation, site, and specialty. Results after imputation were consistent with the analyses without imputation; results without imputation are presented.

Significance levels and adjustment for multiplicity

For hypothesis tests of treatment effects, two-tailed p-values of <0.05 were considered statistically significant. Likelihood ratio tests were used in preference to Wald tests. No formal adjustment for multiplicity was made and the number of statistical tests performed should be considered when interpreting results.

Assessment of the validity of the proportional hazards assumption and model fit for the primary outcome – post-operative length of hospital stay

Global test of proportional hazards assumption: p=0.13

Cardiac specialty

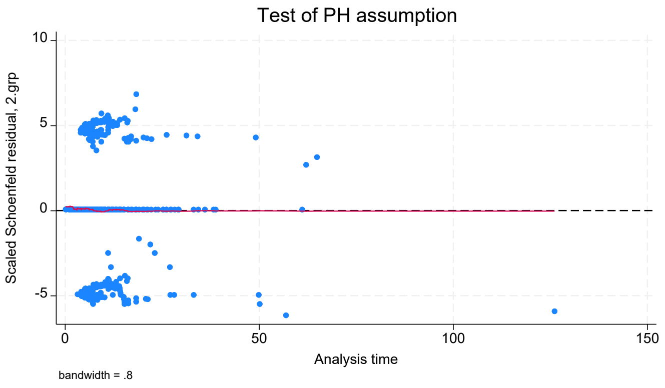

Thoracic specialty

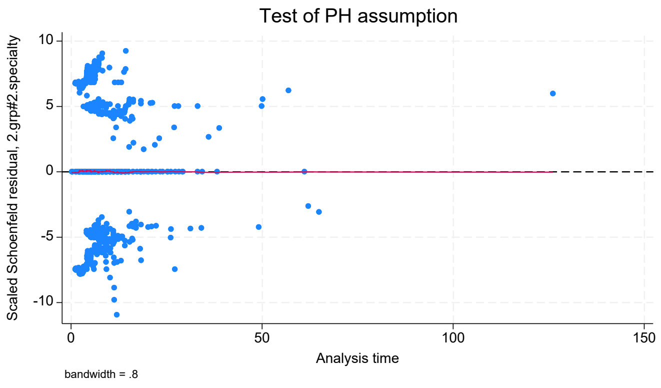

Abdominal specialty

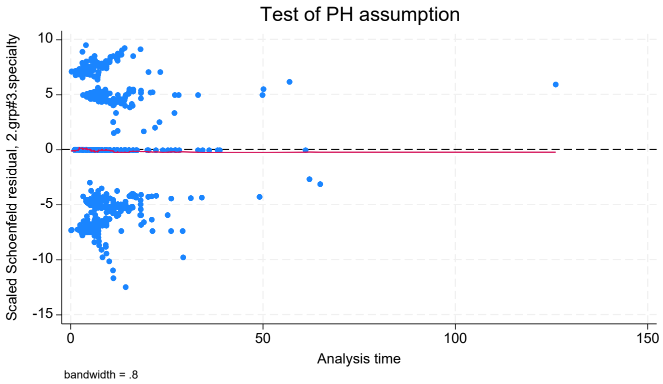

The scaled Schoenfeld residuals plots shown here suggest that the proportional hazards (PH) assumption holds. The smoothed red line remains relatively flat around zero, indicating no clear time-dependent effect.

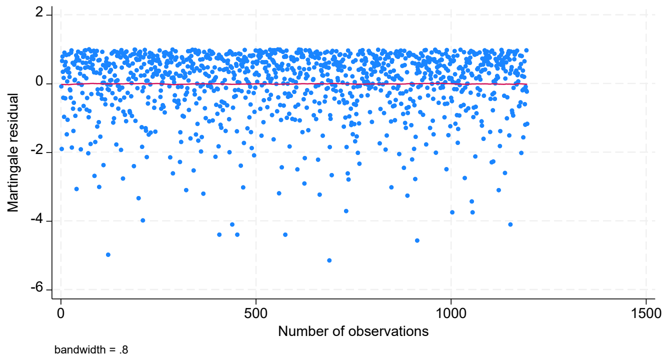

The Martingale residuals plot shown here suggests that the Cox model fits well, as the residuals are mostly centered around zero and the smoothed line remains flat.

Reasons for screened patients being excluded from the trial

Of the 2209 screened patients excluded, 533 (24%) were found to be ineligible at screening, 776 (35%) were not given study information and/or approached by the local team, primarily because they indicated they were not interested in taking part in research, 806 (36%) were approached but declined to take part, and 94 (4%) agreed to take part but did not proceed to randomisation.

Additional Tables

Table 1 Recipient characteristics – additional information

	Cardiac (n=499)		Thoracic (n=346)		Abdominal (n=350)		Overall (n=1195)	
Characteristic	Randomized to Placebo (n=249)	Randomized to Gabapentin (n=250)	Randomized to Placebo (n=172)	Randomized to Gabapentin (n=174)	Randomized to Placebo (n=175)	Randomized to Gabapentin (n=175)	Randomized to Placebo (n=596)	Randomized to Gabapentin (n=599)
**Medical history**								
Congestive cardiac failure	15/248 (6.0%)	23/249 (9.2%)	4/174 (2.3%)	4/174 (2.3%)	2/175 (1.1%)	4/175 (2.3%)	23/595 (3.9%)	31/598 (5.2%)
Peripheral vascular disease	11/248 (4.4%)	10/249 (4.0%)	3/172 (1.7%)	13/174 (7.5%)	1/175 (0.6%)	1/175 (0.6%)	15/595 (2.5%)	24/598 (4.0%)
Cerebrovascular disease	22/248 (8.9%)	18/249 (7.2%)	9/172 (5.2%)	5/174 (2.9%)	8/175 (4.6%)	6/175 (3.4%)	39/595 (6.6%)	29/598 (4.8%)
Hypertension requiring treatment	154/248 (62.1%)	172/249 (69.1%)	68/172 (39.5%)	60/174 (34.5%)	49/175 (28.0%)	49/175 (28.0%)	271/595 (45.5%)	281/598 (47%)
Myocardial infarction	40/248 (6.1%)	48/249 (19.3%)	11/172 (6.4%)	14/174 (8.0%)	7/175 (4.0%)	4/175 (2.3%)	58/595 (9.7%)	66/598 (11%)
Chronic pain syndrome	18/248 (7.3%)	19/249 (7.6%)	30/172 (17.4%)	17/174 (9.8%)	12/175 (6.9%)	10/175 (5.7%)	60/595 (10.1%)	46/598 (7.7%)
Neurological dysfunction	5/248 (2.0%)	9/249 (3.6%)	6/172 (3.5%)	3/174 (1.7%)	2/175 (1.1%)	5/175 (2.9%)	13/595 (2.2%)	17/598 (2.8%)
Chronic pulmonary disease	17/248 (6.9%)	20/249 (8.0%)	34/172 (19.8%)	40/174 (23.0%)	10/175 (5.7%)	14/175 (8.0%)	61/595 (10.3%)	74/598 (12.4%)

	Cardiac (n=499)		Thoracic (n=346)		Abdominal (n=350)		Overall (n=1195)	
**Characteristic**	**Randomized to Placebo (n=249)**	**Randomized to Gabapentin (n=250)**	**Randomized to Placebo (n=172)**	**Randomized to Gabapentin (n=174)**	**Randomized to Placebo (n=175)**	**Randomized to Gabapentin (n=175)**	**Randomized to Placebo (n=596)**	**Randomized to Gabapentin (n=599)**
Rheumatological disease	7/248 (2.8%)	13/249 (5.2%)	21/172 (12.2%)	26/174 (14.9%)	14/175 (8.0%)	15/175 (8.6%)	42/595 (7.1%)	54/598 (9.0%)
Liver disease	2/248 (0.8%)	3/249 (1.2%)	5/172 (2.9%)	3/174 (1.7%)	12/175 (6.9%)	20/175 (11.4%)	19/595 (3.2%)	26/598 (4.3%)
Hemiplegia or paraplegia	0/248 (0.0%)	3/249 (1.2%)	0/172 (0.0%)	1/174 (0.6%)	0/175 (0.0%)	0/175 (0.0%)	0/595 (0.0%)	4/598 (0.7%)
Renal disease	11/248 (4.4%)	7/249 (2.8%)	6/172 (3.5%)	3/174 (1.7%)	7/175 (4%)	2/175 (1.1%)	24/595 (4%)	12/598 (2%)
Malignancies (other than squamous skin cancer)	35/248 (14.1%)	31/249 (12.4%)	120/172 (69.8%)	117/174 (67.2%)	141/175 (80.6%)	144/175 (82.3%)	296/595 (49.7%)	292/598 (48.8%)
**Simple analgesia**								
Paracetamol	44/120 (36.7%)	54/132 (40.9%)	52/65 (80.0%)	44/70 (62.9%)	49/65 (75.4%)	36/45 (80.0%)	145/250 (58.0%)	134/247 (54.3%)
Aspirin	88/120 (73.3%)	98/132 (74.2%)	12/65 (18.5%)	18/70 (25.7%)	11/65 (16.9%)	8/45 (17.8%)	111/250 (44.4%)	124/247 (50.2%)
Ibuprofen	6/120 (5.0%)	9/132 (6.8%)	8/65 (12.3%)	14/70 (20.0%)	10/65 (15.4%)	4/45 (8.9%)	24/250 (9.6%)	27/247 (10.9%)

Table 2 Primary outcome: sensitivity analyses

	Excluding ineligible participants (n=20)		Excluding participants from site with data quality concerns (n=10)	
**Time from surgery to hospital discharge (days)**	**Hazard Ratio (95% CI)**	**P value**	**Hazard Ratio (95% CI)**	**P value**
All participants	1.06 (0.95 – 1.20)		1.07 (0.96 – 1.21)	
Cardiac	1.06 (0.89 – 1.27)		1.07 (0.90 – 1.28)	
Thoracic	1.09 (0.88 – 1.36)		1.09 (0.88 – 1.36)	
Abdominal	1.03 (0.84 – 1.29)		1.05 (0.85 – 1.31)	
Treatment by specialty interaction		0.95		0.97

Table 3 Primary outcome: sub-group analyses

Subgroup	Hazard Ratio (95% CI)	P value	Subgroup	Hazard Ratio (95% CI)	P value	Subgroup	Hazard Ratio (95% CI)	P value
All participants			All participants			All participants		
Minimal access (N =371)	1.05 (0.85 – 1.30)		Female (n=401)	1.19 (0.98 – 1.46)		Pre-COVID-19 (N=441)	1.08 (0.94 – 1.24)	
Open surgery (N=813)	1.07 (0.93 – 1.23)		Male (N=794)	1.01 (0.88 – 1.17)		Post COVID-19 (N=354)	1.04 (0.84 – 1.28)	
Cardiac			Cardiac			Cardiac		
Minimal access (N=0)	-		Female (N=113)	1.12 (0.77 – 1.64)		Pre-COVID-19 (N=468)	1.08 (0.90 – 1.29)	
Open surgery (N=495)	0.71 (0.49 – 1.01)		Male (N=386)	1.17 (0.86 – 1.57)		Post COVID-19 (N=31)	0.97 (0.58 – 1.62)	
Thoracic			Thoracic			Thoracic		
Minimal access (N=218)	1.13 (0.86 – 1.48)		Female (N=157)	1.04 (0.76 – 1.44)		Pre-COVID-19 (N=221)	1.14 (0.87 – 1.49)	
Open surgery (N=124)	0.81 (0.56 – 1.18)		Male (N=189)	0.89 (0.65 – 1.21)		Post COVID-19 (N=125)	0.23 (0.89 – 1.70)	
Abdominal			Abdominal			Abdominal		
Minimal access (N=153)	0.96 (0.69 – 1.32)		Female (N=131)	1.48 (1.04 – 2.12)		Pre-COVID-19 (N=152)	1.01 (0.73 – 1.40)	
Open surgery (N=194)	0.73 (0.54 – 1.00)		Male (N=219)	0.99 (0.73 – 1.34)		Post COVID-19 (N=198)	1.12 (0.81 – 1.54)	
Treatment by specialty by surgery type interaction		0.42	Treatment by specialty by sex interaction		0.13	Treatment by specialty by COVID-19 interaction		0.88
Treatment by specialty interaction		0.96	Treatment by specialty interaction		0.97	Treatment by specialty interaction		0.96
Treatment by surgery type interaction		0.88	Treatment by sex interaction		0.19	Treatment by COVID-19 interaction		0.79
Surgery type by specialty interaction		0.71	Sex by specialty interaction		0.21	COVID-19 by specialty interaction		0.63

CI = confidence interval

Table 4 Secondary outcome: Opioid Use

Outcome	Randomized to placebo	Randomized to gabapentin	Geometric mean ratio (95% CI)	P value
**Surgery to hospital discharge***				
All participants				
Day 0	7.6 (10.6, 4.9-20.9)	6.0 (9.0, 4.0-17.2)		
Day 1	13.0 (16.5, 8.1-33.0)	9.9 (13.2, 5.3-27.3)		
Day 2	4.5 (6.6, 2.0-17.0)	3.3 (6.6, 1.7-13.2)		
Day 3	1.4 (2.2, 0-9.9)	1.4 (3.0, 0-7.9)		
Day 4	1.0 (1.7, 0-6.6)	0.9 (1.4, 0-6.6)		
Day 5	0.8 (0, 0-6.6)	0.6 (0, 0-5.0)		
Cardiac				
Day 0	4.6 (7.0, 4.0-13.0)	5.0 (7.0, 2.6-12.0)	0.92 (0.71, 1.19)	
Day 1	12.3 (14.3, 7.3-27.7)	11.4 (13.7, 6-27.0)	0.88 (0.68, 1.14)	
Day 2	3.0 (5.0, 1.7-12.6)	2.3 (4.3, 0-9.9)	0.85 (0.65, 1.10)	
Day 3	0.8 (0, 0-5.3)	0.9 (1.0, 0-5.0)	1.14 (0.88, 1.49)	
Day 4	0.5 (0, 0-3.0)	0.6 (0, 0-3.6)	0.97 (0.74, 1.27)	
Day 5	0.4 (0, 0-2.0)	0.4 (0, 0-3.3)	0.91 (0.69, 1.21)	
Thoracic				
Day 0	12.1 (15.4, 8.3-24.3)	8.9 (13.2, 6.6-23.2)	0.76 (0.56, 1.02)	
Day 1	13.4 (16.5, 9.1-26.4)	9.4 (12.7, 5.3-21.5)	0.73 (0.54, 0.99)	
Day 2	4.8 (6.8, 3.0-17.2)	4.4 (6.6, 3.0-15.4)	0.70 (0.52, 0.95)	
Day 3	2.6 (4.0, 1.0-13.2)	2.0 (3.3, 0-9.9)	0.95 (0.69, 1.29)	
Day 4	2.9 (4.2, 1.7-10.7)	2.4 (3.6, 1.0-10.9)	0.80 (0.58, 1.11)	
Day 5	2.8 (4.0, 1.0-11.6)	2.9 (3.6, 1.7-9.9)	0.76 (0.54, 1.07)	
Abdominal				
Day 0	10.1 (16.2, 7.3-24.6)	5.4 (9.6, 3.3-18.5)	0.70 (0.52, 0.93)	
Day 1	13.8 (21.8, 9.9-40.3)	8.5 (14.5, 4.4-32.1)	0.67 (0.50, 0.90)	
Day 2	7.3 (11.8, 5.0-25.6)	3.9 (6.9, 1.7-19.8)	0.64 (0.48, 0.86)	
Day 3	2.4 (6.6, 0-13.2)	2.2 (3.3, 0-13.2)	0.87 (0.65, 1.17)	
Day 4	1.9 (4.0, 0-10.0)	1.2 (2.1, 0-10.0)	0.74 (0.54, 1.00)	
Day 5	1.6 (3.3, 0-12.8)	0.9 (0, 0-7.8)	0.69 (0.50, 0.96)	
Treatment by time by specialty interaction				0.80
Treatment by time interaction				0.010
Treatment by specialty interaction				0.001
Time by specialty interaction				<0.001
**During follow-up⁺**				

Outcome	Randomized to placebo	Randomized to gabapentin	Geometric mean ratio (95% CI)	P value
All participants	3.4 (4.0, 0-87.1)	2.8 (3.3, 0-72.6)	0.85 (0.60, 1.21)	
Cardiac	1.7 (0, 0-59.4)	2.0 (0, 0-55.4)	1.12 (0.94, 1.34)	
Thoracic	16.4 (53.5, 3.96-153.9)	10.2 (32.1, 0-178.2)	0.66 (0.27, 1.62)	
Abdominal[#]	2.1 (0, 0-79.2)	1.3 (0, 0-41.6)	0.72 (0.39, 1.35)	
Treatment by specialty interaction				0.22

Summary data are geometric mean and interquartile range

* Unable to calculate due to missing data (placebo, gabapentin): cardiac (2, 1), thoracic (4, 3), abdominal (2,1)

[+] Unable to calculate due to missing data (placebo, gabapentin): cardiac (3, 1), thoracic (4, 4), abdominal (3,1)

[#] Two extreme outliers were excluded from the analysis

CI = confidence interval

Table 5 Secondary outcome: Pain: Numerical Rating Scale

Outcome	Randomized to placebo	Randomized to gabapentin	Mean difference (95% CI)	P value
**Pain at rest**				
All participants				
Baseline[&]	0.66 (0, 0-0)	0.50 (0, 0-0)		
1 hour[+]	3.99 (4, 1-6)	3.48 (4, 1-5)	-0.81 (-1.12, -0.51)	
24 hours[$]	2.87 (3, 1-5)	2.51 (2, 0-4)	-0.25 (-0.42, -0.080)	
48 hours[**]	2.43 (2, 0-4)	2.22 (2, 0-3)	-0.12 (-0.28, 0.043)	
72 hours[++]	1.72 (1, 0-3)	1.66 (1, 0-3)	-0.065 (-0.22, 0.087)	
96 hours[$$]	1.33 (1, 0-2)	1.53 (1, 0-2)	-0.046 (-0.19, 0.10)	
120 hours[***]	1.32 (0, 0-2)	1.20 (0,0-2)	-0.040 (-0.19, 0.11)	
Cardiac				
Baseline				
1 hour[*]	0.22 (0, 0-0)	0.29 (0, 0-0)		
24 hours	2.51 (2, 0-4)	2.28 (2, 0-4)	-0.082 (-0.31, 0.14)	
48 hours	2.02 (2. 0-3)	1.94 (1, 0-3)	-0.031 (-0.25, 0.18)	
72 hours	1.48 (1, 0-2)	1.34 (0, 0-2)	-0.013 (-0.22, 1.94)	
96 hours	1.01 (0, 0-2)	1.39 (0, 0-2)	-0.0097 (-0.22, 0.20)	
120 hours	0.88 (0, 0-1)	1.02 (0, 0-2)	-0.21 (-0.45, 0.027)	
Thoracic				
Baseline	0.95 (0, 0-1)	0.65 (0, 0-0)		
1 hour	3.96 (4, 0-6)	3.25 (3, 1-5)	-0.88 (-1.26, -0.50)	
24 hours	3.37 (3.5, 2-5)	2.88 (2, 1-5)	-0.33 (-0.62, -0.035)	

Outcome	Randomized to placebo	Randomized to gabapentin	Mean difference (95% CI)	P value
48 hours	2.77 (2, 1-5)	2.49 (2, 0-4)	-0.20 (-0.49, 0.10)	
72 hours	2.02 (1, 0-4)	1.91 (2, 0-3)	-0.14 (-0.44, 0.15)	
96 hours	1.88 (1, 0-3)	1.55 (1, 0-2)	-0.13 (-0.42, 0.17)	
120 hours	2.17 (1.5, 0-4)	1.84 (2, 0-3)	-0.12 (-0.42, 0.17)	
Abdominal				
Baseline	1.00 (0, 0-1)	0.64 (0, 0-0)		
1 hour	4.04 (4, 1-7)	3.88 (4, 2-6)	-0.79 (-1.17, -0.42)	
24 hours	2.85 (2, 1-5)	2.45 (2, 0-4)	-0.23 (-0.51, 0.042)	
48 hours	2.73 (2, 1-4)	2.35 (2, 0-3)	-0.10 (-0.37, 0.17)	
72 hours	1.90 (1, 0-3)	1.95 (1, 0-3)	-0.05 (-0.31, 0.21)	
96 hours	1.53 (1, 0-2)	1.76 (1, 0-3)	-0.033 (-0.29, 0.23)	
120 hours	1.73 (1, 0-3)	1.30 (1, 0-2)	-0.030 (-0.29, 0.23)	
Treatment by time by specialty interaction				0.67
Treatment by time interaction				<0.001
Treatment by specialty interaction				0.81
Time by specialty interaction				<0.001
**Pain on movement**				
All participants				
Baseline&&	1.11 (0, 0-1)	0.94 (0, 0-1)		
1 hour#	4.76 (5, 3-7)	4.21 (5,2-6)	-0.82 (-1.20, -0.44)	
24 hours~	4.86 (5, 3-7)	4.46 (5, 2-6)	-0.25 (-0.44, -0.069)	
48 hours^	4.32 (4, 2-6)	4.08 (4, 2-6)	-0.13 (-0.30, 0.041)	
72 hours##	3.20 (3, 1-5)	3.22 (3, 1-5)	-0.059 (-0.23, 0.12)	
96 hours~~	2.67 (2, 0-4)	2.98 (3, 1-5)	-0.009 (-0.19, 0.17)	
120 hours^^	2.28 (2, 0-4)	2.40 (2, 0-4)	0.032 (-0.15, 0.22)	
Cardiac				
Baseline	0.47 (0, 0-0)	0.56 (0, 0-0)		
1 hour*				
24 hours	4.63 (5, 3-7)	4.40 (5. 2-6)	-0.26 (-0.54, 0.016)	
48 hours	4.20 (4, 2-6)	4.15 (4,2-6)	-0.14 (-0.40, 0.12)	
72 hours	3.17 (3, 1-5)	2.99 (3, 1-4)	-0.06 (-0.32, 0.19)	
96 hours	2.29 (2, 0-4)	2.92 (3, 1-5)	-0.013 (-0.27, 0.25)	
120 hours	1.82 (1, 0-3)	2.17 (2, 0-4)	0.029 (-0.23, 0.29)	
Thoracic				
Baseline	1.61 (0, 0-3)	1.23 (0, 0-1)		

Outcome	Randomized to placebo	Randomized to gabapentin	Mean difference (95% CI)	P value
1 hour	4.79 (5, 3-7)	3.97 (4, 1-6)	0.78 (-1.23, -0.33)	
24 hours	5.14 (5, 4-7)	4.71 (5, 3-7)	-0.21 (-0.55, 0.13)	
48 hours	4.27 (4, 2-7)	4.13 (4, 2-6)	-0.086 (-0.43, 0.26)	
72 hours	3.25 (3, 0-5)	3.70 (3.5, 2-5)	-0.012 (-0.36, 0.34)	
96 hours	3.69 (3, 0-6)	3.23 (3, 1-5)	0.038 (-0.32, 0.39)	
120 hours	3.46 (3, 1-5.5)	3.34 (3, 1-5.50)	0.080 (-0.28, 0.44)	
Abdominal				
Baseline	1.52 (0, 0-2)	1.17 (0, 0-1)		
1 hour	4.71 (5, 2-7)	4.63 (5, 3-7)	-0.86 (-1.32, -0.40)	
24 hours	4.89 (5, 3-7)	4.29 (4, 2-6)	-0.29 (-0.61, 0.035)	
48 hours	4.56 (4, 3-7)	3.93 (4, 2-6)	-0.16 (-0.48, 0.15)	
72 hours	3.22 (3, 1-5)	3.23 (3, 1-5)	-0.09 (-0.41, .023)	
96 hours	2.71 (2.5, 1-4)	2.95 (2.5, 0-5)	-0.040 (-0.36, 0.28)	
120 hours	2.55 (2, 0-4)	2.47 (2, 0-4)	0.0013 (-0.32, 0.33)	
Treatment by time by specialty interaction				0.79
Treatment by time interaction				<0.001
Treatment by specialty interaction				0.94
Time by specialty interaction				<0.001

Data are mean (median, IQR)

Differences are gabapentin minus placebo

SD = standard deviation, CI = confidence interval, NRS = numerical rating scale

* Not captured as participants were ventilated after surgery

Not recorded (placebo, gabapentin): & thoracic (3, 3), abdominal (1, 2), cardiac (2, 4), + thoracic (74, 69), abdominal (123, 116), $ cardiac (31, 37), thoracic (13, 20), abdominal (14, 18), ** cardiac (28,39), thoracic (39, 32), abdominal (21, 20), ++ cardiac (39,56), thoracic (84, 85), abdominal (31, 42), $$ cardiac (62, 56), thoracic (107, 121), abdominal (53, 62), *** cardiac (76, 86), thoracic (124, 142), abdominal (86, 87), && thoracic (3, 3), abdominal (1, 2), cardiac (2, 4), # thoracic (78, 72), abdominal (124, 118), ~ cardiac (36, 38), thoracic (14, 22), abdominal (15, 17), ^ cardiac (30, 41), thoracic (40, 34), abdominal (24, 21), ## cardiac (40, 57), thoracic (84, 86), abdominal (33, 43), ~~ cardiac (64, 58), thoracic (107, 121), abdominal (53, 65), ^^ cardiac (77, 87), thoracic (124, 142), abdominal (87, 90)

SD = standard deviation, CI = confidence interval, NRS = numerical rating scale

Note: statistical models used to obtain treatment estimates use all NRS assessments (median 14 assessments), not just those summarised here

Table 6 Analgesia and adjuvants received during trial participation

	Cardiac (n=499)		Thoracic (n=346)		Abdominal (n=350)		Overall (n=1195)	
	Placebo (n=249)	Gabapentin (n=250)	Placebo (n=172)	Gabapentin (n=174)	Placebo (n=175)	Gabapentin (n=175)	Placebo (n=596)	Gabapentin (n=599)
**Per day in hospital**								
**Paracetamol (mg)**								
n/N (%)	246/247 (>99%)	249/249 (100%)	167/168 (99%)	171/171 (100%)	173/173 (100%)	173/174 (99%)	586/588 (>99%)	593/594 (>99%)
Mean (median, IQR)	4553 (4655, 4000-5159)	4713 (4733, 4167-5250)	3858 (3750, 3333-4333)	3808 (3833, 3333-4300)	4240 (4300, 3600-4917)	4177 (4300, 3500-4909)	4263 (4286, 3611-4900)	4296 (4321, 3625-5000)
**Aspirin (mg)**								
n/N (%)	191/247 (77%)	203/249 (82%)	23/168 (14%)	29/171 (17%)	17/173 (10%)	14/174 (8%)	231/588 (39%)	246/594 (41%)
Mean (median, IQR)	99 (75, 75-107)	97 (75, 75-107)	127 (75, 75-75)	83.1 (75, 75-75)	89 (75, 75-75)	102 (75, 75-75)	101 (75, 75-107)	96 (75, 75-107)
**Ibuprofen (mg)**								
n/N (%)	6/247 (2%)	5/249 (2%)	41/168 (24%)	54/171 (32%)	17/173 (10%)	12/174 (7%)	64/588 (11%)	71/594 (12%)
Mean (median, IQR)	714 (767, 400-933)	573 (600, 400-933)	728 (800, 400-914)	806 (900, 600-1000)	781 (946, 400-1000)	734 (800, 400-920)	741 (800, 400-966)	777 (800, 600-1000)
**Codeine (mg)**								
n/N (%)	45/247 (18%)	46/249 (18%)	29/168 (17%)	26/171 (15%)	22/173 (13%)	20/174 (11%)	96/588 (16%)	92/594 (15%)
Mean (median, IQR)	90 (77, 60-120)	86 (90, 60-108)	113 (100, 60-165)	95 (100, 60-120)	111 (94, 60-173)	95 (79, 56-120)	102 (90, 60-150)	90 (90, 60-120)
**Dihydrocodeine (mg)**								
n/N (%)	53/247 (21%)	48/249 (19%)	53/168 (32%)	51/171 (30%)	9/173 (5%)	8/174 (5%)	115/588 (20%)	107/594 (18%)
Mean (median, IQR)	59 (60, 47-70)	61 (59, 40-76)	72 (75, 60-90)	69 (70, 45-90)	65 (70, 51-75)	56 (60, 40-65)	65 (60, 50-80)	64 (60, 45-84)
**Alfentanil (mg)**								
n/N (%)	40/247 (16%)	54/249 (22%)	3/168 (2%)	1/171 (1%)	7/173 (4%)	9/174 (5%)	50/588 (9%)	64/594 (11%)
Mean (median, IQR)	9 (5, 3-14)	8 (7, 3-11)					9 (5, 2-14)	8 (7, 3-12)

	Cardiac (n=499)		Thoracic (n=346)		Abdominal (n=350)		Overall (n=1195)	
	**Placebo (n=249)**	**Gabapentin (n=250)**	**Placebo (n=172)**	**Gabapentin (n=174)**	**Placebo (n=175)**	**Gabapentin (n=175)**	**Placebo (n=596)**	**Gabapentin (n=599)**
**Tramadol (oral and IV) (mg)**								
n/N (%)	98/247 (40%)	112/249 (45%)	97/168 (58%)	99/171 (58%)	108/173 (62%)	100/174 (57%)	303/588 (52%)	311/594 (52%)
Mean (median, IQR)	134 (100, 59-183)	128 (100, 75-175)	265 (250, 150-338)	239 (200, 125-350)	262 (243, 150-379)	253 (250, 150-339)	221 (200, 100-310)	203 (175, 100-288)
**Fentanyl (IV, intrathecal and epidural) (mg)**								
n/N (%)	229/247 (93%)	217/249 (87%)	126/168 (75%)	134/171 (78%)	126/173 (73%)	132/174 (76%)	481/588 (82%)	483/594 (81%)
Mean (median, IQR)	1.3 (1.0, 0.8-1.5)	1.3 (1.3, 0.8-1.5)	0.4 (0.4, 0.3-0.5)	0.4 (0.4, 0.3-0.4)	1.1 (0.3, 0.2-0.5)	0.3 (0.3, 0.2-0.4)	1.0 (0.5, 0.3-1.0)	0.7 (0.5, 0.3-1.0)
**Diamorphine (IV and intrathecal) (mg)**								
n/N (%)	3/247 (1%)	3/249 (1%)	0/168 (0%)	1/171 (1%)	12/173 (7%)	13/174 (7%)	15/588 (3%)	17/594 (3%)
Mean (median, IQR)					0.5 (0.5, 0.5-0.5)	1.0 (0.5, 0.4-0.5)	3.9 (0.5, 0.5-0.7)	1.9 (0.5, 0.5-2.5)
**Morphine (oral and IV) (mg)**								
n/N (%)	234/247 (95%)	239/249 (96%)	144/168 (86%)	136/171 (80%)	134/173 (77%)	127/174 (73%)	512/588 (87%)	502/594 (85%)
Mean (median, IQR)	23 (18, 10-31)	21 (17, 10-30)	34 (30, 16-45)	30 (23, 15-40)	32 (23, 13-43)	26 (23, 11-34)	29 (22, 11-38)	25 (20, 11-33)
**Oxycodone (oral and IV) (mg)**								
n/N (%)	40/247 (16%)	36/249 (14.5%)	69/168 (41%)	69/171 (40%)	66/173 (38%)	66/174 (38%)	175/588 (30%)	171/594 (29%)
Mean (median, IQR)	20 (15, 7-24)	14 (10, 5-20)	17 (10, 6-15)	17 (10, 7-16)	25 (17, 10-33)	21 (17, 10-27)	21 (12, 7-26)	18 (10, 8-21)
**Buprenorphine (sublingual and patch)^ (mg)**								
n/N (%)	1/247 (<1%)	1/249 (<1%)	0/168 (0%)	0/171 (0%)	3/173 (2%)	0/174 (0%)	4/588 (1%)	1/594 (<1%)
**Pethidine# (mg)**								
n/N (%)	4/247 (2%)	1/249 (<1%)	0/168 (0%)	0/171 (0%)	0/173 (0%)	1/174 (1%)	4/588 (1%)	2/594 (<1%)
**Per day during follow-up**								
**Paracetamol (mg)**								

	Cardiac (n=499)		Thoracic (n=346)		Abdominal (n=350)		Overall (n=1195)	
	**Placebo (n=249)**	**Gabapentin (n=250)**	**Placebo (n=172)**	**Gabapentin (n=174)**	**Placebo (n=175)**	**Gabapentin (n=175)**	**Placebo (n=596)**	**Gabapentin (n=599)**
n/N (%)	239/246 (97%)	236/246 (96%)	166/169 (98%)	161/169 (95%)	153/172 (89%)	154/174 (89%)	558/587 (95%)	551/589 (94%)
Mean (median, IQR)	2678 (2027, 2000-4000)	2570 (2000, 2000-4000)	3085 (3603, 2027-4000)	3316 (4000, 2373-4000)	3122 (4000, 2000-4000)	3083 (4000, 2000-4000)	2919 (2618, 2000-4000)	2923 (2560, 2000-4000)
**Aspirin (mg)**								
n/N (%)	180/246 (73%)	190/246 (77%)	21/169 (12%)	32/169 (19%)	15/172 (9%)	14/174 (8%)	216/587 (37%)	236/589 (40%)
Mean (median, IQR)	80 (75, 75-75)	82 (75, 75-75)	75 (75, 75-75)	75 (75, 75-75)	75 (75, 75-75)	87 (75, 75-75)	79 (75, 75-75)	81 (75, 75-75)
**Ibuprofen (mg)**								
n/N (%)	5/246 (2%)	7/246 (3%)	35/169 (21%)	47/169 (28%)	21/172 (12%)	13/174 (7%)	61/587 (10%)	67/589 (11%)
Mean (median, IQR)			943 (1200, 600-1200)	1029 (1200, 848-1200)	720 (600, 600-1200)	1008 (1200, 600-1200)	872 (800, 600-1200)	1005 (1200, 604-1200)
**Codeine (mg)**								
n/N (%)	71/246 (29%)	76/246 (31%)	45/169 (27%)	49/169 (29%)	29/172 (17%)	31/174 (18%)	145/587 (25%)	156/589 (26%)
Mean (median, IQR)	119 (120, 60-120)	106 (120, 60-120)	93 (70, 50-120)	77 (60, 32-120)	79 (60, 60-113)	88 (60, 30-120)	103 (98, 60-120)	94 (101, 60-120)
**Dihydrocodeine (mg)**								
n/N (%)	39/246 (16%)	39/246 (16%)	57/169 (34%)	49/169 (29%)	7/172 (4%)	8/174 (5%)	103/587 (18%)	96/589 (16%)
Mean (median, IQR)	89 (60, 60-120)	86 (60, 60-120)	112 (120, 120-120)	113 (120, 120-120)			103 (120, 60-120)	104 (120, 60-120)
**Alfentanil**								
n/N (%)	1/246 (<1%)	0/246 (0%)	0/169 (0%)	0/169 (0%)	0/172 (0%)	0/174 (0%)	1/587 (<1%)	0/589 (0%)
**Tramadol (mg)**								
n/N (%)	26/246 (11%)	23/246 (9%)	59/169 (35%)	51/169 (30%)	47/172 (27%)	48/174 (28%)	132/587 (22%)	122/589 (21%)
Mean (median, IQR)	180 (200, 100-200)	154 (200, 100-200)	173 (199, 100-200)	203 (200, 109-200)	160 (100, 100-200)	162 (100, 100-200)	170 (150, 100-200)	178 (200, 100-200)

	Cardiac (n=499)		Thoracic (n=346)		Abdominal (n=350)		Overall (n=1195)	
	Placebo (n=249)	Gabapentin (n=250)	Placebo (n=172)	Gabapentin (n=174)	Placebo (n=175)	Gabapentin (n=175)	Placebo (n=596)	Gabapentin (n=599)
**Fentanyl (mg)**								
n/N (%)	1/246 (<1%)	1/246 (<1%)	0/169 (0%)	1/169 (1%)	2/172 (1%)	0/174 (0%)	3/587 (1%)	2/589 (<1%)
**Morphine (short acting) (mg)**								
n/N (%)	10/246 (4%)	3/246 (1%)	34/169 (20%)	27/169 (16%)	11/172 (6%)	12/174 (7%)	55/587 (9%)	42/589 (7%)
Mean (median, IQR)	15 (13, 5-20)	30 (30, 20-40)	29 (20, 20-40)	34 (20, 20-40)	17 (10, 10-20)	18 (20, 20-20)	24 (20, 10-40)	31 (20, 20-40)
**Morphine (prolonged release) (mg)**								
n/N (%)	0/246 (0%)	1/246 (0.4%)	2/169 (1.2%)	0/169 (0%)	3/172 (1.7%)	0/174 (0%)	5/587 (0.9%)	1/589 (0.2%)
**Oxycodone (Short acting) (mg)**								
n/N (%)	2/246 (1%)	1/246 (<1%)	6/169 (4%)	9/169 (5%)	6/172 (3%)	3/174 (2%)	14/587 (2%)	13/589 (2%)
Mean (median, IQR)							31 (19, 15-30)	21 (15, 9-30)
**Oxycodone (Prolonged release) (mg)**								
n/N (%)	1/246 (<1%)	0/246 (0%)	3/169 (2%)	4/169 (2%)	1/172 (1%)	1/174 (1%)	5/587 (1%)	5/589 (1%)
**Buprenorphine (mg)**								
n/N (%)	1/246 (<1%)	1/246 (<1%)	1/169 (1%)	0/169 (0%)	3/172 (2%)	0/174 (0%)	5/587 (1%)	1/589 (<1%)
**Pregabalin (mg)**								
n/N (%)	1/246 (<1%)	1/246 (<1%)	5/169 (3%)	2/169 (1%)	1/172 (1%)	1/174 (1%)	7/587 (1%)	4/589 (1%)
**Gabapentin (mg)**								
n/N (%)	6/246 (2%)	1/246 (<1%)	7/169 (4%)	9/169 (5%)	3/172 (2%)	1/174 (1%)	16/587 (3%)	11/589 (2%)
Mean (median, IQR)							976 (525, 300-628)	811 (721, 300-900)

IV = intravenous

Doses are given where at least 10 participants were taking the medication

Table 7 Secondary outcome: Pain: Brief Pain Inventory

Outcome	Randomized to placebo		Randomized to gabapentin		Odds ratio for pain present (95% CI)	Geometric mean ratio where present (95% CI)	P value
	Present	Score if present	Present	Score if present			
**Pain severity index**							
All participants							
Baseline	216/571 (37.8%)	12 (7-19)	205/585 (35.0%)	10 (6-16)			
4 weeks+	295/506 (53.3%)	11 (7-18)	319/503 (63.4%)	11 (7-18)	1.42 (1.15 – 1.75)	0.99 (0.90 – 1.08)	
4 months$	182/505 (33.2%)	12.5 (7–20)	211/485 (40.6%)	11 (6-19)			
Cardiac							
Baseline	82/233 (35.2%)	10 (6-16)	87/243 (35.8%)	10 (6-14)			
4 weeks	131/215 (60.9%)	11 (7-16)	148/222 (66.7%)	11 (7-16)	1.35 (0.98 – 1.86)	0.98 (0.86 – 1.12)	
4 months	74/223 (33.2%)	12 (7-19)	88/217 (40.6%)	10 (6-15)			
Thoracic							
Baseline	57/166 (34.3%)	14 (9-20)	53/173 (30.6%)	10 (6-19)			
4 weeks	94/150 (62.7%)	14 (8-19)	90/132 (68.2%)	15 (9-20)	1.63 (1.10 – 2.42)	1.08 (0.93 – 1.26)	
4 months	50/133 (37.6%)	14 (7-20)	66/128 (51.6%)	13 (7-21)			
Abdominal							
Baseline	77/172 (44.8%)	12 (8-20)	65/169 (38.5%)	12 (5-17)			
4 weeks	70/141 (49.6%)	10.5 (7-19)	81/149 (54.4%)	9 (5-16)	1.34 (0.91 – 1.97)	0.90 (0.73 – 1.10)	
4 months	58/149 (38.9%)	12 (8-20)	57/140 (40.7%)	11 (4-20)			
Treatment by time by specialty interaction							0.34
Treatment by time interaction							0.77
Treatment by specialty interaction							0.24
Time by specialty interaction							0.003

Outcome	Randomized to placebo		Randomized to gabapentin		Odds ratio for pain present (95% CI)	Geometric mean ratio where present (95% CI)	P value
	Present	Score if present	Present	Score if present			
**Pain interference index**							
All participants							
Baseline	214/584 (36.6%)	19.5 (9-34)	185/592 (31.3%)	19 (8-33)			
4 weeks[++]	299/525 (57.0%)	23 (10-37)	320/516 (62.0%)	22 (11-36)	1.38 (1.12 – 1.70)	1.07 (0.94 – 1.22)	
4 months[$$]	186/331 (36.0%)	21.5 (7-36)	206/390 (41.5%)	21 (8-35)			
Cardiac							
Baseline	86/242 (35.5%)	16.5 (9-33)	86/247 (34.8%)	17 (8-34)			
4 weeks	135/227 (59.5%)	21 (9 -36)	148/228 (64.9%)	22.5 (12-36)	1.31 (0.94 – 1.82)	1.14 (0.93 – 1.39)	
4 months	77/229 (33.6%)	19 (5-35)	87/223 (39.0%)	18 (7-30)			
Thoracic							
Baseline	56/169 (33.1%)	25 (8.5-34)	45/174 (25.9%)	19 (7-34)			
4 weeks	93/152 (61.2%)	25 (11-38)	92/136 (67.6%)	23.5 (14.5-37)	1.70 (1.15 – 2.51)	1.14 (0.93 – 1.40)	
4 months	51/138 (37.0%)	26 (9-39)	65/130 (50.0%)	23 (12-36)			
Abdominal							
Baseline	72/173 (41.6%)	18.5 (9-35)	54/171 (31.6%)	18 (8-28)			
4 weeks	71/146 (48.6%)	22 (11-39)	80/152 (52.6%)	16 (9-33.5)	1.22 (0.84 – 1.76)	0.90 (0.69 – 1.16)	
4 months	58/150 (38.7%)	22.5 (8-36)	54/143 (37.8%)	18 (7-35)			
Treatment by time by specialty interaction							0.44
Treatment by time interaction							0.92
Treatment by specialty interaction							0.28
Time by specialty interaction							0.002

Data are n/N (%) and median (interquartile range)

Ratios are gabapentin/placebo

CI = confidence interval

Missing (placebo, gabapentin): [+] cardiac (24, 21), thoracic (23, 35), abdominal (27, 22), [$] cardiac (20, 26), thoracic (35, 43), abdominal (31, 35), [++] cardiac (23, 21), thoracic (22, 36), abdominal (27, 22), [$$] cardiac (20, 29), thoracic (41, 49), abdominal (30, 38)

Table 8 Secondary outcome: Quality of Life -EQ-5D-5L and SF-12

Outcome	Randomized to placebo	Randomized to gabapentin	Mean difference (95% CI)	P value
**EQ-5D-5L utility score**				
All participants				
Baseline	0.77 (0.20)	0.77 (0.19)		
4 weeks+	0.71 (0.20)	0.69 (0.20)	-0.014 (-0.033 –+0.005)	
4 months$	0.78 (0.22)	0.77 (0.22)		
Cardiac				
Baseline	0.74 (0.21)	0.73 (0.19)		
4 weeks	0.68 (0.21)	0.66 (0.21)	-0.015 (-0.043 – +0.014)	
4 months	0.81 (0.20)	0.79 (0.21)		
Thoracic				
Baseline	0.80 (0.19)	0.82 (0.16)		
4 weeks	0.71 (0.18)	0.67 (0.20)	-0.024 (-0.060 - +0.012)	
4 months	0.71 (0.25)	0.72 (0.23)		
Abdominal				
Baseline	0.79 (0.19)	0.79 (0.20)		
4 weeks	0.75 (0.18)	0.74 (0.19)	-0.009 (-0.044 - +0.026)	
4 months	0.79 (0.21)	0.79 (0.22)		
Treatment by time by specialty interaction				0.35
Treatment by time interaction				0.39
Treatment by specialty interaction				0.83
Time by specialty interaction				<0.001
**SF-12 physical component score**				
All participants				
Baseline	46.0 (10.2)	46.3 (10.6)		
4 weeks++	39.1 (8.83)	38.5 (9.63)	-0.87 (-1.71 – -0.04)	
4 months$$	47.3 (9.54)	46.3 (9.98)		
Cardiac				
Baseline	42.9 (9.66)	42.0 (9.73)		
4 weeks	37.2 (7.97)	36.6 (8.94)	-0.63 (-1.89 – +0.63)	
4 months	48.3 (8.82)	47.0 (10.1)		
Thoracic				
Baseline	48.0 (10.0)	48.0 (10.7)		
4 weeks	39.7 (8.59)	37.8 (9.45)	-1.73 (-3.33 – -0.12)	
4 months	45.2 (9.24)	43.1 (9.99)		
Abdominal				

Outcome	Randomized to placebo	Randomized to gabapentin	Mean difference (95% CI)	P value
Baseline	48.4 (10.1)	50.8 (9.17)		
4 weeks	41.4 (9.71)	42.0 (9.92)	-0.45 (-2.00 – +1.10)	
4 months	47.6 (10.6)	47.9 (9.21)		
Treatment by time by specialty interaction				0.98
Treatment by time interaction				0.53
Treatment by specialty interaction				0.47
Time by specialty interaction				<0.001
**SF-12 mental component score**				
All participants				
Baseline	50.5 (9.45)	50.1 (9.74)		
4 weeks++	48.7 (10.3)	49.2 (10.4)	0.74 (-0.39, 1.87)	
4 months$$	51.9 (9.61)	51.2 (9.75)	-0.55 (-1.61, 0.51)	
Cardiac				
Baseline	50.1 (9.44)	49.0 (9.84)		
4 weeks	48.7 (10.5)	49.0 (10.7)	-0.0006 (-1.51, 1.51)	
4 months	53.3 (8.58)	51.2 (10.1)	-1.28 (-2.72, 0.16)	
Thoracic				
Baseline	50.8 (10.2)	51.5 (8.79)		
4 weeks	47.7 (10.7)	48.2 (10.3)	1.45 (-0.37, 3.28)	
4 months	49.8 (11.3)	50.9 (9.79)	0.17 (-1.61, 1.96)	
Abdominal				
Baseline	50.6 (8.68)	50.4 (10.3)		
4 weeks	49.7 (9.49)	50.4 (9.87)	1.18 (-0.60, 2.95)	
4 months	51.7 (9.10)	51.5 (9.21)	-0.10 (-1.83, 1.62)	
Treatment by time by specialty interaction				0.20
Treatment by time interaction				0.056
Treatment by specialty interaction				0.35
Time by specialty interaction				0.070

Data are mean (standard deviation)

Differences are gabapentin minus placebo

+ Missing (placebo, gabapentin): + cardiac (24, 21), thoracic (23, 35), abdominal (27, 22), $ cardiac (20, 26), thoracic (35, 43), abdominal (31, 35), ++ cardiac (24, 23), thoracic (25, 40), abdominal (29, 23), $$ cardiac (20, 26), thoracic (34, 43), abdominal (26, 33)

CI = confidence interval, SF-12 = short-form 12

Table 9 Adverse events experienced during trial participation

	Cardiac (n=499)				Thoracic (n=346)				Abdominal (n=350)				Overall (n=1195)			
**Event (MedDRA system organ class)**	**Randomized to Placebo (n=249)**		**Randomized to Gabapentin (n=250)**		**Randomized to Placebo (n=172)**		**Randomized to Gabapentin (n=174)**		**Randomized to Placebo (n=175)**		**Randomized to Gabapentin (n=175)**		**Randomized to Placebo (n=596)**		**Randomized to Gabapentin (n=599)**	
	**AE**	**SAE**	**AE**	**SAE**	**AE**	**SAE**	**AE**	**SAE**	**AE**	**SAE**	**AE**	**SAE**	**AE**	**SAE**	**AE**	**SAE**
1 or more event	243/249 (97.6%)	78/249 (31.3%)	245/250 (97.7%)	82/250 (32.7%)	91/172 (53.0%)	44/172 (25.6%)	75/346 (43.2%)	55/174 (31.6%)	99/175 (56.6%)	67/175 (38.3%)	100 (57.2%)	58/175 (33.1%)	433/596 (72.2%)	189/599 (31.7%)	420/599 (70.0%)	195/599 (32.5%)
Number of events	1082	187	1335	216	164	83	133	111	207	144	220	178	1453	414	1488	505
	**Events/participants (% of participants)**		**Events/participants (% of participants)**		**Events/participants (% of participants)**		**Events/participants (% of participants)**		**Events/participants (% of participants)**		**Events/participants (% of participants)**		**Events/participants (% of participants)**		**Events/participants (% of participants)**	
Blood & lymphatic system disorders	14/14 (5.6%)	2/1 (0.4%)	6/6 (2.4%)	1/1 (0.4%)	1/1 (0.6%)		1/1 (0.6%)	1/1 (0.6%)	4/4 (2.3%)	1/1 (0.6%)	3/3 (1.7%)	2/1 (0.6%)	19/19 (3.2%)	3/2 (0.3%)	10/10 (1.7%)	4/3 (0.5%)
Cardiac disorders	149/131 (52.6%)	43/33 (13.3%)	139/116 (46.2%)	40/30 (12.0%)	19/19 (11.0%)	2/2 (1.2%)	2/2 (1.1%)	2/2 (1.1%)	3/3 (1.7%)	2/2 (1.1%)	6/6 (3.4%)	7/6 (3.4%)	171/153 (25.7%)	47/37 (6.2%)	147/124 (20.7%)	49/38 (6.3%)
Arrhythmia	17/16 (6.4%)	6/5 (2.0%)	19/19 (7.6%)	4/3 (1.2%)	8/8 (4.7%)	1/1 (0.6%)							26/25 (4.2%)	7/6 (1.0%)	23/23 (3.8%)	7/6 (1.0%)
Atrial fibrillation	80/80 (32.1%)	10/10 (4.0%)	74/74 (29.5%)	12/11 (4.4%)	10/10 (5.8%)								92/92 (15.4%)	10/10 (1.7%)	76/76 (12.7%)	15/14 (2.3%)
Pericardial effusion	20/20 (8.0%)	16/15 (6.0%)	14/14 (5.6%)	10/8 (3.2%)									20/20 (3.4%)	17/16 (2.7%)	14/14 (2.3%)	10/8 (1.3%)
Gastrointestinal disorders	82/59 (23.7%)	11/8 (3.2%)	90/64 (25.5%)	12/11 (4.4%)	28/22 (12.8%)	3/3 (1.7%)	27/22 (12.6%)	6/6 (3.4%)	98/62 (35.4%)	43/30 (17.1%)	102/62 (35.4%)	79/42 (24.0%)	208/143 (24.0%)	57/41 (6.9%)	219/148 (24.7%)	97/59 (9.8%)

	Cardiac (n=499)				Thoracic (n=346)				Abdominal (n=350)				Overall (n=1195)			
Event (MedDRA system organ class)	Randomized to Placebo (n=249)		Randomized to Gabapentin (n=250)		Randomized to Placebo (n=172)		Randomized to Gabapentin (n=174)		Randomized to Placebo (n=175)		Randomized to Gabapentin (n=175)		Randomized to Placebo (n=596)		Randomized to Gabapentin (n=599)	
	AE	SAE	AE	SAE	AE	SAE	AE	SAE	AE	SAE	AE	SAE	AE	SAE	AE	SAE
	Events/participants (% of participants)		Events/participants (% of participants)		Events/participants (% of participants)		Events/participants (% of participants)		Events/participants (% of participants)		Events/participants (% of participants)		Events/participants (% of participants)		Events/participants (% of participants)	
Abdominal pain									8/8 (4.6%)	1/1 (0.6%)			12/12 (2.0%)	4/4 (0.7%)	7/7 (1.2%)	8/8 (1.3%)
Constipation	16/16 (6.4%)	2/2 (0.8%)	26/26 (10.4%)	-	8/8 (4.7%)	2/2 (1.1%)	10/10 (5.7%)	1/1 (0.6%)					28/28 (4.7%)	4/4 (0.7%)	39/39 (6.5%)	1/1 (0.2%)
Diarrhea			9/9 (3.6%)	5/5 (2.0%)			1/1 (0.6%)	12/10 (5.7%)					10/10 (1.7%)	8/7 (1.2%)	14/14 (2.3%)	18/16 (2.7%)
Ileus					-	11/10 (5.7%)	-	20/20 (11.4%)	15/15 (8.6%)	2/2 (1.2%)	13/13 (7.4%)	-	16/16 (2.7%)	14/13 (2.2%)	16/16 (2.7%)	20/20 (3.3%)
Nausea	27/26 (10.4%)	1/1 (0.4%)	20/20 (8.0%)	-	12/12 (7.0%)	2/2 (1.1%)	13/13 (7.5%)	8/8 (4.6%)	35/35 (20.0%)	-	36/36 (20.6%)	-	74/73 (12.2%)	3/3 (0.5%)	69/69 (11.5%)	8/8 (1.3%)
Vomiting	20/20 (8.0%)	1/1 (0.4%)	21/21 (8.4%)	-	6/6 (3.5%)	4/4 (2.3%)	3/3 (1.7%)	12/12 (6.9%)	29/29 (16.6%)	-	39/38 (21.7%)	1/1 (0.6%)	55/55 (9.2%)	5/5 (0.8%)	63/62 (10.3%)	13/13 (2.2%)
General disorders & administration site conditions	69/59 (23.7%)	7/7 (2.8%)	85/72 (28.7%)	8/6 (2.4%)	10/10 (5.8%)	2/1 (0.6%)	10/9 (5.2%)	5/5 (2.9%)	15/15 (8.6%)	14/13 (7.4%)	15/14 (8.0%)	4/4 (2.3%)	94/84 (14.1%)	23/21 (3.5%)	110/95 (15.8%)	17/15 (2.5%)
Oedema peripheral	12/12 (4.8%)	1/1 (0.4%)	13/13 (5.2%)	1/1 (0.4%)									14/14 (2.3%)	10/9 (1.5%)	15/15 (2.5%)	5/5 (0.8%)

	Cardiac (n=499)				Thoracic (n=346)				Abdominal (n=350)				Overall (n=1195)			
Event (MedDRA system organ class)	Randomized to Placebo (n=249)		Randomized to Gabapentin (n=250)		Randomized to Placebo (n=172)		Randomized to Gabapentin (n=174)		Randomized to Placebo (n=175)		Randomized to Gabapentin (n=175)		Randomized to Placebo (n=596)		Randomized to Gabapentin (n=599)	
	AE	SAE	AE	SAE	AE	SAE	AE	SAE	AE	SAE	AE	SAE	AE	SAE	AE	SAE
	Events/participants (% of participants)		Events/participants (% of participants)		Events/participants (% of participants)		Events/participants (% of participants)		Events/participants (% of participants)		Events/participants (% of participants)		Events/participants (% of participants)		Events/participants (% of participants)	
Pain	14/14 (5.6%)	2/2 (0.8%)	14/14 (5.6%)	-	6/6 (3.5%)	8/7 (4.0%)			10/10 (5.7%)		7/7 (4.0%)	4/4 (2.3%)	30/30 (5.0%)	10/9 (1.5%)	27/27 (4.5%)	5/5 (0.8%)
Post procedural fever	34/31 (12.4%)	1/1 (0.4%)	46/46 (18.3%)	5/3 (1.2%)									37/34 (5.7%)	5/5 (0.8%)	51/51 (8.5%)	5/3 (0.5%)
Infections & infestations	47/41 (16.5%)	36/25 (10.0%)	58/52 (20.7%)	35/27 (10.8%)	9/8 (4.7%)	27/18 (10.5%)	10/9 (5.2%)	15/14 (8.0%)	10/9 (5.1%)	24/21 (12.0%)	19/15 (8.6%)	22/20 (11.4%)	66/58 (9.7%)	87/64 (10.7%)	87/76 (12.7%)	72/61 (10.2%)
Infection					1/1 (0.6%)	10/10 (5.7%)							6/6 (1.0%)	19/17 (2.9%)	14/14 (2.3%)	13/13 (2.2%)
Pneumonia			15/15 (6.0%)	5/5 (2.0%)			5/5 (2.9%)	6/6 (3.4%)	1/1 (0.6%)	11/11 (6.4%)			9/9 (1.5%)	18/17 (2.9%)	24/24 (4.0%)	16/16 (2.7%)
Respiratory tract infection	28/28 (11.2%)	14/13 (5.2%)	24/24 (9.6%)	16/14 (5.6%)					4/4 (2.3%)	8/6 (3.5%)			36/36 (6.0%)	24/21 (3.5%)	33/33 (5.5%)	20/17 (2.8%)
Injury, poisoning & procedural complications	82/67 (26.9%)	10/7 (2.8%)	73/60 (23.9%)	20/13 (5.2%)	14/14 (8.1%)	18/14 (8.1%)	7/7 (4.0%)	16/13 (7.5%)	12/11 (6.3%)	40/30 (17.1%)	12/12 (6.9%)	34/21 (12.0%)	108/92 (15.4%)	68/51 (8.6%)	92/79 (13.2%)	70/47 (7.8%)
Post procedural hemorrhage	69/62 (24.9%)	1/1 (0.4%)	55/47 (18.7%)	3/2 (0.8%)									71/64 (10.7%)	8/8 (1.3%)	56/48 (8.0%)	5/4 (0.7%)

	Cardiac (n=499)				Thoracic (n=346)				Abdominal (n=350)				Overall (n=1195)			
Event (MedDRA system organ class)	Randomized to Placebo (n=249)		Randomized to Gabapentin (n=250)		Randomized to Placebo (n=172)		Randomized to Gabapentin (n=174)		Randomized to Placebo (n=175)		Randomized to Gabapentin (n=175)		Randomized to Placebo (n=596)		Randomized to Gabapentin (n=599)	
	AE	SAE	AE	SAE	AE	SAE	AE	SAE	AE	SAE	AE	SAE	AE	SAE	AE	SAE
	Events/participants (% of participants)		Events/participants (% of participants)		Events/participants (% of participants)		Events/participants (% of participants)		Events/participants (% of participants)		Events/participants (% of participants)		Events/participants (% of participants)		Events/participants (% of participants)	
Surgical procedure repeated					2/2 (1.2%)	11/11 (6.3%)	-	22/14 (8.0%)	1/1 (0.6%)	9/9 (5.2%)			5/5 (0.8%)	22/22 (3.7%)	3/3 (0.5%)	34/25 (4.2%)
Wound infection					-	11/10 (5.7%)							10/10 (1.7%)	16/14 (2.3%)	9/9 (1.5%)	15/13 (2.2%)
Investigations	48/46 (18.5%)	4/3 (1.2%)	46/44 (17.5%)	4/4 (1.6%)	2/2 (1.2%)		1/1 (0.6%)	2/2 (1.1%)	2/2 (1.1%)	1/1 (0.6%)	6/6 (3.4%)	2/1 (0.6%)	52/50 (8.4%)	5/4 (0.7%)	53/51 (8.5%)	8/7 (1.2%)
Weight increased	39/39 (15.7%)	1/1 (0.4%)	32/32 (12.7%)	-									39/39 (6.5%)	1/1 (0.4%)	32/32 (5.3%)	-
Liver function test increased			14/13 (5.2%)	4/4 (1.6%)									9/7 (1.2%)	4/3 (0.5%)	20/19 (3.2%)	5/5 (0.8%)
Metabolism & nutrition disorders	37/35 (14.1%)	3/3 (1.2%)	31/31 (12.4%)	5/4 (1.6%)	3/3 (1.7%)		5/5 (2.9%)	1/1 (0.6%)	4/4 (2.3%)	1/1 (0.6%)	1/1 (0.6%)		44/42 (7.0%)	4/4 (0.7%)	37/37 (6.2%)	6/5 (0.8%)
Hyperglycemia	22/22 (8.8%)	-	24/24 (9.6%)	-									24/24 (4.0%)	1/1 (0.2%)	24/24 (4.0%)	-

	Cardiac (n=499)				Thoracic (n=346)				Abdominal (n=350)				Overall (n=1195)			
**Event (MedDRA system organ class)**	**Randomized to Placebo (n=249)**		**Randomized to Gabapentin (n=250)**		**Randomized to Placebo (n=172)**		**Randomized to Gabapentin (n=174)**		**Randomized to Placebo (n=175)**		**Randomized to Gabapentin (n=175)**		**Randomized to Placebo (n=596)**		**Randomized to Gabapentin (n=599)**	
	**AE**	**SAE**	**AE**	**SAE**	**AE**	**SAE**	**AE**	**SAE**	**AE**	**SAE**	**AE**	**SAE**	**AE**	**SAE**	**AE**	**SAE**
	**Events/participants (% of participants)**		**Events/participants (% of participants)**		**Events/participants (% of participants)**		**Events/participants (% of participants)**		**Events/participants (% of participants)**		**Events/participants (% of participants)**		**Events/participants (% of participants)**		**Events/participants (% of participants)**	
Nervous system disorders	30/25 (10.0%)	6/6 (2.4%)	32/27 (10.8%)	5/5 (2.0%)	8/7 (4.1%)	1/1 (0.6%)	10/10 (5.7%)	5/5 (2.9%)	12/12 (6.9%)		16/14 (8.0%)	5/5 (2.9%)	50/44 (7.4%)	7/7 (1.2%)	58/51 (8.5%)	15/15 (2.5%)
Somnolence	3/3 (1.2%)		7/7 (2.8%)		1/1 (0.6%)		2/2 (1.1%)		-		2/2 (1.1%)		4/4 (0.7%)		11/11 (1.8%)	
Psychiatric disorders	29/26 (10.4%)	4/4 (1.6%)	27/25 (10.0%)	9/7 (2.8%)	8/6 (3.5%)	3/1 (0.6%)	9/8 (4.6%)	3/2 (1.1%)	12/10 (5.7%)		9/9 (5.1%)	1/1 (0.6%)	49/42 (7.0%)	7/5 (0.8%)	45/42 (7.0%)	13/10 (1.7%)
Delirium	13/13 (5.2%)	-	7/7 (1.4%)	6/5 (2.0%)	1/1 (2.9%)	-	-	2/2 (1.1%)	3/3 (1.7%)	-	1/1 (0.6)	1/1 (0.6)	17/17 (2.9%)	-	8/8 (1.3%)	9/8 (1.3%)
Renal & urinary disorders	22/22 (8.8%)	8/6 (2.4%)	35/31 (12.4%)	5/5 (2.0%)	17/15 (8.7%)	1/1 (0.6%)	22/21 (12.1%)		12/12 (6.9%)	6/6 (3.4%)	9/9 (5.1%)	6/6 (3.4%)	51/49 (8.2%)	15/13 (2.2%)	66/61 (10.2%)	11/11 (1.8%)
Urinary retention					14/13 (7.6%)	6/6 (3.4%)	21/21 (12.1%)	3/3 (1.7%)					27/26 (4.4%)	8/8 (1.3%)	37/37 (6.2%)	4/4 (0.7%)
Acute kidney injury	17/17 (6.8%)	6/4 (1.6%)	25/25 (10.0%)	4/4 (1.6%)									22/22 (3.7%)	7/5 (0.8%)	29/29 (4.8%)	7/7 (1.2%)
Respiratory, thoracic & mediastinal disorders	106/85 (34.1%)	24/15 (6.0%)	105/89 (35.5%)	31/22 (8.8%)	38/35 (20.3%)	11/10 (5.8%)	22/20 (11.5%)	33/23 (13.2%)	8/7 (4.0%)	7/5 (2.9%)	12/12 (6.9%)	7/5 (2.9%)	152/127 (21.3%)	42/30 (5.0%)	139/121 (20.2%)	71/50 (8.3%)

	Cardiac (n=499)				Thoracic (n=346)				Abdominal (n=350)				Overall (n=1195)			
**Event (MedDRA system organ class)**	**Randomized to Placebo (n=249)**		**Randomized to Gabapentin (n=250)**		**Randomized to Placebo (n=172)**		**Randomized to Gabapentin (n=174)**		**Randomized to Placebo (n=175)**		**Randomized to Gabapentin (n=175)**		**Randomized to Placebo (n=596)**		**Randomized to Gabapentin (n=599)**	
	**AE**	**SAE**	**AE**	**SAE**	**AE**	**SAE**	**AE**	**SAE**	**AE**	**SAE**	**AE**	**SAE**	**AE**	**SAE**	**AE**	**SAE**
	**Events/participants (% of participants)**		**Events/participants (% of participants)**		**Events/participants (% of participants)**		**Events/participants (% of participants)**		**Events/participants (% of participants)**		**Events/participants (% of participants)**		**Events/participants (% of participants)**		**Events/participants (% of participants)**	
Atelectasis	34/34 (13.7%)	3/3 (1.2%)	33/33 (13.1%)	4/4 (1.6%)									40/40 (6.7%)	5/5 (0.8%)	38/38 (6.3%)	4/4 (0.7%)
Respiratory depression	-		1/1 (0.4%)		1/1 (0.6%)		-		-		1/1 (0.6%)		1/1 (0.2%)		2/2 (0.3%)	
Pleural effusion	50/50 (20.1%)	17/12 (4.8%)	63/62 (24.7%)	18/16 (6.4%)									55/55 (9.2%)	21/15 (2.5%)	68/67 (11.2%)	24/22 (3.7%)
Pneumothorax	15/14 (5.6%)	-			13/13 (7.6%)	1/1 (0.6%)							28/27 (4.5%)	5/5 (0.8%)	11/11 (1.8%)	8/7 (1.2%)
Bronchopleural fistula					15/15 (8.7%)	-	9/9 (5.2%)	-			-	10/9 (5.2%)	18/18 (3.0%)	2/2 (0.3%)	10/10 (1.7%)	10/9 (1.5%)
Surgical & medical procedures	126/117 (47.0%)	18/11 (4.4%)	126/112 (44.6%)	24/18 (7.2%)	3/3 (1.7%)	1/1 (0.6%)		9/5 (2.9%)	2/2 (1.1%)	1/1 (0.6%)		5/5 (2.9%)	131/122 (20.5%)	20/13 (2.2%)	126/112 (18.7%)	38/28 (4.7%)
Continuous positive airway pressure	20/17 (6.8%)	2/2 (0.8%)	18/18 (7.2%)	1/1 (0.4%)									21/18 (3.0%)	2/2 (0.3%)	18/18 (3.0%)	3/3 (0.5%)

	Cardiac (n=499)				Thoracic (n=346)				Abdominal (n=350)				Overall (n=1195)			
Event (MedDRA system organ class)	Randomized to Placebo (n=249)		Randomized to Gabapentin (n=250)		Randomized to Placebo (n=172)		Randomized to Gabapentin (n=174)		Randomized to Placebo (n=175)		Randomized to Gabapentin (n=175)		Randomized to Placebo (n=596)		Randomized to Gabapentin (n=599)	
	AE	SAE	AE	SAE	AE	SAE	AE	SAE	AE	SAE	AE	SAE	AE	SAE	AE	SAE
	Events/participants (% of participants)		Events/participants (% of participants)		Events/participants (% of participants)		Events/participants (% of participants)		Events/participants (% of participants)		Events/participants (% of participants)		Events/participants (% of participants)		Events/participants (% of participants)	
Pacemaker generated rhythm	104/103 (41.4%)	8/8 (3.2%)	100/99 (39.4%)	10/10 (4.0%)									104/103 (17.3%)	8/8 (1.3%)	100/99 (16.5%)	10/10 (1.7%)
Vascular disorders	224/186 (74.7%)	9/7 (2.8%)	264/201 (80.1%)	13/10 (4.0%)	3/3 (1.7%)	4/4 (2.3%)	3/3 (1.7%)	7/5 (2.9%)	6/4 (2.3%)	4/4 (2.3%)	3/3 (1.7%)	2/1 (0.6%)	233/193 (32.4%)	17/15 (2.5%)	270/207 (34.5%)	22/16 (2.7%)
Hypertension	64/61 (24.5%)	-	59/56 (22.3%)	-									64/61 (10.2%)	-	59/56 (9.3%)	-
Vasodilatation	155/141 (56.6%)	4/4 (1.6%)	201/178 (70.9%)	5/4 (1.6%)									158/144 (24.2%)	4/4 (0.7%)	205/182 (30.3%)	7/6 (1.0%)
Death		1/249		3/250		7/172		5/174		0/175		2/175		8/596		10/599

System organ classes with fewer than 30 events in total across all groups are omitted

Within each system organ class data are given (shaded rows) where at least 5% of participants experienced the event in the surgical specialty (i.e. AE or SAE) along with the totals for the study as a whole. Details of clinicial important events with lower frequency, i.e. somnolence and respiratory depression, are also given.

Data are number of events in number of participants (% of participants), for example 233 adverse events classified as vascular disorders occurred in 193 participants in the placebo group, 32.4% of all participants in that group, in addition 17 serious adverse events occurred in 15 participants, 2.5% of participants in the placebo group

AE = adverse event, SAE = serious adverse event

Table 10 Secondary outcome: one or more serious adverse events

Serious adverse events (SAE)	Randomized to placebo		Randomized to gabapentin		Risk difference (95% CI)	P value	Risk ratio (95% CI)	P value
All participants								
One or more SAE	189/596	31.7%	195/599	32.6%				
Cardiac								
One or more SAE	78/249	31.3%	82/250	32.8%	+0.015 (-0.021 – +0.050)		1.05 (0.92 – 1.20)	
Thoracic								
One or more SAE	44/172	25.6%	55/174	31.6%	+0.060 (+-0.013 – +0.107)		1.23 (1.04 – 1.47)	
Abdominal								
One or more SAE	67/175	38.3%	58/175	33.2%	-0.051 (-0.095 – -0.008)		0.86 (0.76 – 0.98)	
Treatment by specialty interaction						0.0006		0.0027

Data are n/N (%)

Differences are gabapentin minus placebo
